# The Amadori Rearrangement for Carbohydrate Conjugation: Scope and Limitations

**DOI:** 10.1002/ejoc.201600458

**Published:** 2016-06-27

**Authors:** Cornelia Hojnik, Anne Müller, Tobias‐Elias Gloe, Thisbe K. Lindhorst, Tanja M. Wrodnigg

**Affiliations:** ^1^ Institute of Organic ChemistryGraz University of TechnologyStremayrgasse 98010GrazAustria; ^2^ Otto Diels Institute of Organic ChemistryChristiana Albertina University of KielOtto‐Hahn‐Platz24118KielGermany

**Keywords:** Rearrangement, Glycoconjugates, Carbohydrates, Glycopeptides, Synthetic methods

## Abstract

The Amadori rearrangement was investigated for the synthesis of *C*‐glycosyl‐type neoglycoconjugates. Various amines including diamines, amino‐functionalized glycosides, lysine derivatives, and peptides were conjugated with two different heptoses to generate non‐natural *C*‐glycosyl‐type glycoconjugates of the d‐*gluco* and d‐*manno* series. With these studies, the scope and limitations of the Amadori rearrangement as a conjugation method have been exemplified with respect to the carbohydrate substrate, as well as the amino components.

## Introduction

Carbohydrates constitute one of the most important classes of biomolecules in nature, where they have manifold functions. They serve as a source of energy and as structural material in plants, and they also play a key role in cell–cell recognition and communication,^1^ cellular differentiation,^2^ and immune response,^3^ both in healthy and disease states of living organisms.

Carbohydrates, which serve functions in cell–cell interactions, are typically covalently bound to biomolecules such as proteins, peptides and lipids to form various glycoconjugates. Glycopeptides, in particular, are well known for their biological functions including antibacterial and antibiotic activity.^4^ They are formed by the anomeric conjugation of carbohydrates with the side chains of specific amino acids. For the investigation of their multi‐facetted biological functions, synthetic access to glycopeptides and mimetics thereof is desired. Thus, suitable conjugation methods have been widely explored in recent years,^5^ including biorthogonal reactions such as native chemical ligation,^6^ Staudinger ligation,^7^ Diels–Alder ligation,^8^ tetrazine ligation,^9^ as well as different “click chemistry” approaches.^10^

**Scheme 1:**
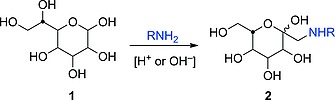
Amadori rearrangement furnishes *C*‐glycosyl‐type neoglycoconjugates (1‐amino‐1‐deoxy ketoses **2**) from aldoses **1**.

We have demonstrated earlier that the Amadori rearrangement can also be considered as a versatile synthetic method for the preparation of glycoconjugates. This reaction allows the conjugation of aldoses **1** to amino components achieving *C*‐glycosyl‐type neoglycoconjugates such as **2** (Scheme 1).^11^

The conjugation through a *C*‐glycosidic linkage is of particular interest for biological investigations, because this type of linkage is not sensitive towards enzymatic hydrolysis, in contrast to the more common *O*‐ and *N*‐glycosidic bonds. In spite of this, the Amadori rearrangement has not been extensively employed in the past because of several challenges accompanying this reaction. For example, the reversibility of several steps including the introduction of the amine and the subsequent isomerization to the aminodeoxy ketose product often leads to low yields. Furthermore, the rearrangement product can enter the Maillard reaction cascade,^12^ leading to a range of side and degradation products. In addition, the formation of anomeric mixtures of both furanoside and pyranoside Amadori products causes difficulties during the isolation of one desired isomer. Nevertheless, we have earlier shown the versatility of the Amadori rearrangement in various applications;[11b], [11c], 13 therefore, we commenced a program to further investigate the scope and limitations of this rearrangement as a new glycoconjugation method. Herein, we focus on the use of (i) diamines, (ii) more complex amino‐functionalized glycosides, and (iii) amino acids, as amino components for the Amadori rearrangement to achieve more complex *C*‐glycosyl‐type glycoconjugates.

## Results and Discussion

### Amadori Rearrangement with Diamines

**Scheme 2:**
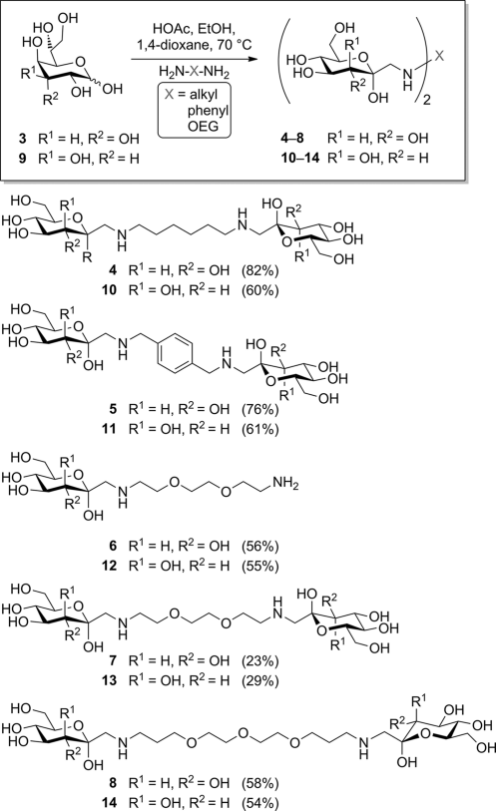
Double Amadori rearrangement with different diamines employing heptoses **3** and **9** of the d‐*gluco* and d‐*manno* series, respectively. OEG = oligo(ethylene glycol).

To optimize the reaction conditions for a double Amadori rearrangement, which has not been investigated as yet, commercially available d‐*glycero*‐d‐*gulo* aldoheptose (**3**) and 1,6‐diaminohexane were employed (Scheme 2). The Amadori rearrangement of **3** was performed with 0.5 equiv. of the diamine and 1 equiv. of acetic acid in ethanol with 1,4‐dioxane as co‐solvent. This led to a double Amadori rearrangement at both amino groups to give *N*,*N′*‐bis‐(1‐deoxy‐α‐d‐glucohept‐2‐ulopyranosyl)‐1,6‐diaminohexane (**4**) with a high yield of 82 % after purification. However, chromatographic purification required a two‐step protocol employing ion exchange chromatography on CG‐120‐II (Na^+^) Amberlite® resin using a water/NH_4_OH gradient and subsequent conventional silica gel chromatography.

To investigate the scope of the reaction with respect to the sugar moieties, d‐*glycero*‐d‐*galacto* aldoheptose (**9**), synthesized in six steps from d‐mannose,[13c] was used as an alternative heptose to **3**, giving access to d‐*manno*‐configured Amadori rearrangement products (Scheme 2). In previous studies, such d‐*manno*‐configured neoglycoconjugates were tested as inhibitors of bacterial adhesion.[13c] Treatment of aldoheptose **9** with 1,6‐diaminohexane, under the same reaction conditions exemplified with aldoheptose **3**, gave, after 2 d at 70 °C, the disubstituted product **10** in 60 % yield.

When *p*‐xylylenediamine was employed as the amino component under the same reaction conditions, heptose **3** gave *N*,*N′*‐bis(1‐deoxy‐α‐d‐glucohept‐2‐ulopyranosyl)‐*p*‐xylylenediamine (**5**) in 76 % yield. With substrate **9**, the corresponding rearrangement product **11** was isolated in 61 % yield.

The analogous reaction with 2,2′‐(ethylenedioxy)bis(ethylamine) gave rearrangement product **7** in only 23 % yield and the corresponding monosubstituted Amadori rearrangement product **6**, which was concomitantly obtained in 56 % yield. In this case, the low yield of **7** might be explained by the limited availability of the second amino group, which remains free after the first Amadori rearrangement, leading to **6**. Likewise, when sugar substrate **9** was employed with 2,2′‐(ethylenedioxy)bis(ethylamine) under the same reaction conditions, only 29 % of the bis‐compound **13** was isolated, whereas the corresponding mono‐compound **12** was isolated in a yield of 55 %. On the other hand, when 4,7,10‐trioxa‐1,13‐tridecanediamine was employed as the amino component and an excess of aldoheptose **3** (3 equiv.) was used, the Amadori rearrangement gave exclusively the disubstituted product **8** in a yield of 58 %. Aldoheptose **9** gave, under the same reaction conditions, exclusively product **14** in a yield of 54 %. Hence, the amount of monosubstitution vs. disubstitution of diamines can be controlled by selecting the amount of aldose in a quantifiable manner. Additionally, the availability of the second amino group increases with the chain length of the spacer of the respective diamines.

During our investigation applying various amines in the Amadori rearrangement, we observed an H/D exchange in the NMR spectra at position C‐1. This isotopic exchange was previously detected by Heyns and co‐workers^14^ who noted that signals of protons at the position C‐1 in Amadori rearrangement compounds decreased on prolonged storage of solutions in D_2_O because of H/D exchange, which significantly accelerated with increasing pH values^15^ (see the Supporting Information).

**Scheme 3:**
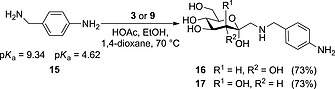
Diamine **15**, having two differing p*K*_a_ values, allows for regioselective Amadori rearrangement.

Inspired by the success of the double Amadori rearrangement with *p*‐xylylenediamine, we next investigated the use of 4‐aminobenzylamine (**15**) as a bidirectional linker. Here, we aimed at a regioselective single Amadori rearrangement involving the benzylic amino group, whereas the arylamino group was intended to remain available for subsequent modifications. Indeed, the reaction of both aldoheptoses **3** and **9** with diamine **15** gave exclusively the singly modified rearrangement products **16** and **17** in 73 % in both cases (Scheme 3).

The regioselective ligation reaction with **15** can be rationalized on the basis of the p*K*_a_ values of the amino group. Given that basicity is a crucial parameter for the nucleophilicity of amines, which is required for the success of the Amadori rearrangement, (4‐aminobenzyl)amine (**15**), with p*K*_a_ values of 9.3 for the benzylic amine and 4.6 for the aniline amino group, reacts selectively at the benzylic position. Products **16** and **17**, respectively, can be further ligated in amide coupling reactions, thiourea bridging or other ligation reactions involving amines. In addition, the amino group can be converted into an azido function, which is amenable for click chemistry.

### Amadori Rearrangement with Amino‐Functionalized Carbohydrates

**Scheme 4:**
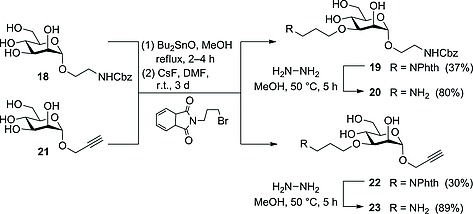
Regioselective synthesis of the amino‐functionalized mannosides **20** and **23**.

To utilize the Amadori rearrangement for the synthesis of more complex glycoconjugates, amino‐functionalized glycosides were used as amino components. To this end, 3‐*O*‐(aminopropyl)‐functionalized mannosides **20** and **23** were synthesized by applying a procedure for tin‐mediated regioselective etherification of glycosides, which we have disclosed earlier (Scheme 4).^16^ Thus, mannosides **18** 17 and **21**,^18^ respectively, were treated with dibutyltin oxide to achieve the corresponding stannylidene acetal intermediates. These were treated with *N*‐(3‐bromopropyl)phthalimide in the same reaction vessel after the solvent was exchanged from MeOH to *N*,*N*‐dimethylformamide (DMF) to deliver the respective 3‐*O*‐functionalized mannosides **19** and **22** after regioselective opening of the tin acetal ring. Although the yields were moderate in this step, this is an advantageous direct approach to selectively 3‐*O*‐functionalize mannosides without the need for protecting‐group chemistry. The free amines **20** and **23** were obtained after hydrazinolysis^16^ of **19** and **22** in 80 and 89 % yields, respectively.

**Scheme 5:**
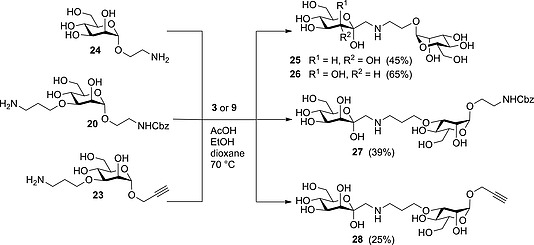
Synthesis of complex glycoconjugates employing Amadori rearrangement of heptoses **3** or **9** with amino‐functionalized mannosides **20**, **23**, and **24** as amino components.

In the next step, amino‐functionalized mannosides **20** and **23** as well as known 2‐aminoethyl α‐d‐mannopyranoside (**24**)^19^ were employed in the Amadori rearrangement with d‐*glycero*‐d‐*gulo* aldoheptose (**3**) as well as d‐*glycero*‐d‐*galacto* aldoheptose (**9**) (Scheme 5). First, 2‐aminoethyl α‐d‐mannopyranoside (**24**)^19^ was treated with **3** and **9** to obtain the rearrangement products **25** in a yield of 45 % and **26** in 65 % yield, respectively. Next, in the d‐*manno* series employing substrate **9**, 2‐(benzyloxycarbonylamino)ethyl 3‐*O*‐(3‐aminopropyl) α‐d‐mannopyranoside (**20**) gave Amadori rearrangement compound **27** in 39 % yield. Propynyl‐3‐*O*‐(3‐aminopropyl) α‐d‐mannopyranoside (**23**) gave compound **28** in 25 % yield. In these cases, the reactions had to be stopped after 5 d at 70 °C to avoid side‐product formation and degradation, and, consequently, the obtained yields were rather moderate. Nevertheless, both compounds are valuable intermediates, because they offer orthogonal groups at the anomeric position for further modification: compound **27**, bearing a masked amino function, and compound **28**, a versatile propargyl group.

### Synthesis of Amino Acid Glycoconjugates by Amadori Rearrangement

**Scheme 6:**
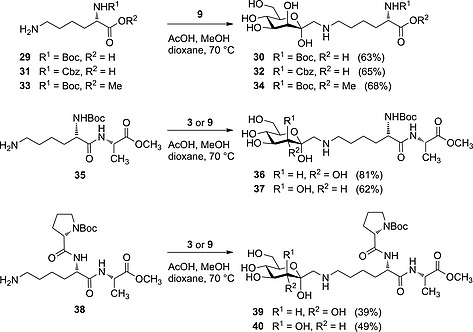
Amadori rearrangement employing lysine derivatives **29**, **31**, and **33** as well as dipeptide **35** and tripeptide **38**.

We then investigated the use of the Amadori rearrangement as a conjugation method for the synthesis of *C*‐glycosyl‐type amino acid glycoconjugates. Thus, we employed partly protected lysine derivatives **29**, **31**, and **33** as well as dipeptide **35** and tripeptide **38** as amino components (Scheme 6).

When d‐*glycero*‐d‐*galacto* aldoheptose (**9**) was treated with *N*_α_‐(*tert*‐butoxycarbonyl)‐l‐lysine (**29**) under acidic conditions, the corresponding Amadori rearrangement product **30** was obtained in a yield of 63 %. Using *N*_α_‐(benzyloxycarbonyl)‐l‐lysine (**31**) as amino component, the d‐*gluco*‐configured product was obtained in 73 %,[11b] and the d‐*manno*‐configured product **32** was isolated in 65 % yield. Likewise, methyl *N*_α_‐(*tert*‐butoxycarbonyl)‐l‐lysinate (**33**) was treated with aldoheptose **9** to obtain the desired rearrangement product **34** in 68 % yield.

We wanted to employ dipeptides as well as tripeptides as amino components for the Amadori rearrangement (Scheme 6). Therefore, the l‐lysine‐containing di‐ and tripeptides **35** and **38**, respectively, were synthesized by applying standard peptide coupling procedures.^20^ Dipeptide **35** employed in the Amadori rearrangement of d‐*glycero*‐d‐*gulo* aldoheptose (**3**) and d‐*glycero*‐d‐*galacto* aldoheptose (**9**) gave compounds **36** (81 %) and **37** (62 %), respectively. Likewise, tripeptide **38** furnished glycopeptide mimetics **39** (39 %) and **40** (49 %) from the aldoheptoses **3** and **9**, respectively.

## Conclusions

We demonstrated that the Amadori rearrangement is an attractive conjugation method for the synthesis of *C*‐glycosyl‐type glycoconjugate mimetics without the need for protecting‐group manipulations.

Symmetrical diamino components gave the respective disubstituted Amadori rearrangement products in acceptable yields. Interestingly, the ratio of mono‐sugar conjugation vs. bis‐sugar Amadori products can be controlled in a preparatively useful manner by controlling the amount of sugar substrate employed. The regioselectivity of the Amadori rearrangement employing unsymmetrical diamines can be controlled by taking the p*K*_a_ value as a parameter for the nucleophilicity of the respective amino components into account. Amines with a p*K*_a_ range between 8 and 12 are more efficient nucleophiles for this reaction than less basic amines. Amino‐functionalized glycosides can also be employed as amino components in the Amadori reaction. By the choice of the aglycon, such as a masked amino function or a versatile propargyl group, different applications for the obtained building blocks can be envisaged. Lysine derivatives as well as lysine‐containing di‐ and tripeptides in the Amadori rearrangement lead to *C*‐glycosyl‐type glycopeptide mimetics, which can be used as building blocks for glycopeptide and glycoprotein synthesis. In all cases the α‐anomeric configuration of the respective Amadori products was obtained exclusively, as has been shown earlier in the d‐*gluco* as well as d‐*manno* series.[11b], [11c] In the context of configuration of the sugar substrate, no significant differentiation in the obtained yields was observed.

Despite the fact that yields for this conjugation method may occasionally be found in a preparatively moderate range and product purification may be extensive in a few cases, conjugation through a *C*‐glycosidic linkage leads to versatile building blocks for different applications, in particular for biological investigations, because this type of conjugation is not sensitive towards enzymatic or chemical hydrolysis in a physiological environment.

## Experimental Section

**Materials and General Methods:** All chemicals were purchased from Sigma Aldrich and used without further purification. Moisture‐sensitive reactions were carried out under nitrogen in dry glassware. NMR spectra were recorded with Bruker Ultrashield spectrometers at 300.36 (^1^H) and 75.53 (^13^C) MHz, respectively. Higher resolution NMR spectra were recorded with VARIAN INOVA 500 MHz at 500.619 (^1^H) and 125.894 (^13^C) MHz, respectively. Chemical shifts are reported relative to internal tetramethylsilane (*δ* = 0.00 ppm), D_2_O (*δ* = 4.79 ppm), [D_6_]DMSO (*δ* = 2.50 ppm) or [D_4_]MeOH (*δ* = 4.78, 3.31 ppm). Full assignment of the peaks was achieved with the aid of 2D NMR techniques (^1^H‐^1^H COSY and ^1^H‐^13^C HSQC). Optical rotations were measured with a Perkin–Elmer 341 polarimeter (sodium D‐line: 589 nm, length of cell: 1 dm, temp.: 20 °C) in the solvents indicated. MALDI‐TOF mass spectrometry was performed with a Micromass TofSpec 2E time‐of‐flight mass spectrometer. Thin‐layer chromatography was performed on precoated silica gel plates on aluminum 60 F254 (E. Merck 5554). Detection was effected by UV and/or charring with 10 % sulfuric acid in EtOH as well as with ceric ammonium molybdate (100 g of ammonium molybdate/8 g of ceric sulfate in 1 L of 10 % H_2_SO_4_) followed by heat treatment at ca. 180 °C. Flash chromatography was performed on silica gel 60 (0.035–0.070 mm, 60 A, Acros Organics 24036) using distilled solvents. Ion exchange chromatography was performed with a strong cation exchanger (Amberlite® CG‐120‐II, Na^+^ form, 200–400 mesh, Fluka 06449) using water and a water/NH_4_OH conc. mixture.

**General Method A (Diamines):** To a solution of the respective aldoheptose (2 equiv.) in a mixture of EtOH and 1,4‐dioxane, the amino compound (1 equiv.) and acetic acid (2 equiv.) were added, and the reaction mixture was stirred at 70 °C until TLC showed satisfactory consumption of the starting material. The reaction mixture was concentrated under reduced pressure, and the crude product was purified by ion exchange chromatography as well as silica gel chromatography with the solvent system indicated.

**General Method B (Amines):** To a solution of the respective aldoheptose (1 equiv.) in a mixture of EtOH and 1,4‐dioxane, the amino compound and acetic acid (1 equiv.) were added, and the reaction mixture was stirred at 70 °C until TLC showed satisfactory consumption of the starting material. The reaction mixture was concentrated under reduced pressure, and the crude product was purified by silica gel column chromatography with the solvent system indicated.

***N*,*N′*‐Bis(1‐deoxy‐α‐d‐*gluco*‐hept‐2‐ulopyranosyl)‐1,6‐diaminohexane (4):** By applying general method A, d‐*glycero*‐d‐*gulo*‐heptopyranose (**3**; 300 mg, 1.43 mmol, 2 equiv.) was treated with 1,6‐diaminohexane (83 mg, 0.714 mmol, 1 equiv.) in EtOH (8 mL) and 1,4‐dioxane as co‐solvent in the presence of acetic acid (82 µL, 1.43 mmol, 2 equiv.) at 70 C for 2 d. The solvents were removed under reduced pressure, and subsequent column chromatography (CHCl_3_/MeOH, 1:1, v/v containing 50 % of conc. NH_4_OH) gave disubstituted product **4** (294 mg, 0.587 mmol, 82 %). [*α*]_D_^20^ = +27.3 (*c* = 1.06, H_2_O). ^1^H NMR (300 MHz, D_2_O): *δ* = 3.90–3.63 (m, 8 H, 7‐H, 7′‐H, 6‐H, 4‐H), 3.44 (d, *J*_3,4_ = 9.2 Hz, 2 H, 3‐H), 3.38 (dd, *J*_5,4_ = 9.4, *J*_5,6_ = 10.4 Hz, 2 H, 5‐H), 3.05 (d, *J*_1,1′_ = 12.6 Hz, 2 H, 1‐H), 2.96 (dd, 2 H, 1′‐H), 2.80 (t, 4 H, 8‐H), 1.58 (br. s, 4 H, 9‐H), 1.36 (br. s, 4 H, 10‐H) ppm. ^13^C NMR (75 MHz, D_2_O): *δ* = 96.3 (C‐2), 73.5, 72.4, 72.3 (3 C, C‐6, C‐4, C‐3), 69.5 (C‐5), 60.7 (C‐7), 53.6 (C‐1), 48.9 (C‐8), 26.9 (C‐9), 25.8 (C‐10) ppm. HRMS (MALDI): *m/z* calcd. for C_20_H_40_N_2_O_12_ [M + H]^+^ 501.2660; found 501.2665.

***N*,*N′*‐Bis(1‐deoxy‐α‐d‐*gluco*‐hept‐2‐ulopyranosyl)‐*p*‐xylylenediamine (5):** By applying general method A, d‐*glycero*‐d‐*gulo*‐heptopyranose (**3**; 300 mg, 1.43 mmol, 2 equiv.) was treated with *p*‐xylylenediamine (97 mg, 0.714 mmol, 1 equiv.) in EtOH (5 mL) and 1,4‐dioxane as co‐solvent in the presence of acetic acid (82 µL, 1.43 mmol, 2 equiv.) at 70 °C for 2 d. The solvents were removed under reduced pressure, and the crude product was passed through an ion exchange CG‐120‐II (Na+) Amberlite® resin column (H_2_O; H_2_O containing 1 % of conc. NH_4_OH) followed by purification by silica gel column chromatography (CHCl_3_/MeOH, 1:1, v/v containing 25 % of conc. NH_4_OH) to give disubstituted product **5** (281 mg, 0.540 mmol, 76 %). [*α*]_D_^20^ = +34.3 (*c* = 1.70, H_2_O). ^1^H NMR (300 MHz, D_2_O): *δ* = 7.43–7.36 (br. s, 4 H, Ph), 3.92 (br. s, 4 H, 8‐H), 3.84–3.61 (m, 8 H, 7‐H, 7′‐H, 6‐H, 4‐H), 3.40 (d, *J*_3,4_ = 9.4 Hz, 2 H, 3‐H), 3.36 (dd, *J*_4,5_ = 9.2, *J*_5,6_ = 9.3 Hz, 2 H, 5‐H), 2.97 (d, *J*_1,1′_ = 12.5 Hz, 2 H, 1‐H), 2.89 (d, 2 H, 1′‐H) ppm. ^13^C NMR (125 MHz, H_2_O/D_2_O, 9:1 v/v): *δ* = 136.0, 129.3 (6 C, phenyl), 96.5 (C‐2), 73.6, 72.4, 72.3 (3 C, C‐6, C‐4, C‐3), 69.5 (C‐5), 60.7 (C‐7), 53.0 (C‐1), 52.1 (C‐8) ppm. HRMS (MALDI): *m/z* calcd. for C_22_H_36_N_2_O_12_ [M + Na]^+^ 543.2166; found 543.2173.

***N*,*N′*‐Bis(1‐deoxy‐α‐d‐*gluco*‐hept‐2‐ulopyranosyl)‐2,2′‐ethylenedioxybis(ethylamine) (7):** By applying general method A, d‐*glycero*‐d‐*gulo*‐heptopyranose (**3**; 400 mg, 1.90 mmol, 2 equiv.) was treated with 2,2′‐ethylenedioxybis(ethylamine) (139 µL, 0.952 mmol, 1 equiv.) in EtOH (6 mL) and 1,4‐dioxane as co‐solvent in the presence of acetic acid (109 µL, 1.90 mmol, 2 equiv.) at 70 °C for 2 d. The solvents were removed under reduced pressure, and the crude product was passed through an ion exchange CG‐120‐II (Na+) Amberlite® resin column (H_2_O; H_2_O containing 1 % of conc. NH_4_OH) followed by purification by silica gel column chromatography (CHCl_3_/MeOH, 3:1, v/v containing 1 % of conc. NH_4_OH) to give disubstituted compound **7** (118 mg, 0.222 mmol, 23 %) and monosubstituted product **6** (180 mg, 0.529 mmol, 56 %).

**Compound 7:** [*α*]_D_^20^ = +22.7 (*c* = 2.23, H_2_O). ^1^H NMR (300 MHz, D_2_O): *δ* = 3.84–3.64 (m, 12 H, 10‐H, 7‐H, 7′‐H, 6‐H, 4‐H), 3.62 (t, 4 H, 9‐H), 3.41 (d, *J*_3,4_ = 9.4 Hz, 2 H, 3‐H), 3.38 (dd, *J*_5,4_ = 9.0, *J*_5,6_ = 9.4 Hz, 2 H, 5‐H), 2.89 (d, *J*_1,1′_ = 12.8 Hz, 2 H, 1‐H), 2.80 (t, 4 H, 8‐H), 2.75 (d, 2 H, 1′‐H) ppm. ^13^C NMR (75 MHz, D_2_O): *δ* = 97.3 (C‐2), 73.7, 72.2, 72.1 (3 C, C‐6, C‐4, C‐3), 69.6 (C‐5), 69.5, 69.4 (2 C, C‐9, C‐10), 60.8 (C‐7), 53.9 (C‐1), 48.2 (C‐8) ppm. HRMS (MALDI): *m/z* calcd. for C_20_H_40_D_2_N_2_O_14_ [M + Na]^+^ 559.2690; found 559.2695.

**Compound 6:** ^1^H NMR (300 MHz, D_2_O): *δ* = 3.85–3.58 (m, 12 H, 12‐H, 11‐H, 10‐H, 9‐H, 7‐H, 7′‐H, 6‐H, 4‐H), 3.42 (d, *J*_3,4_ = 9.4 Hz, 1 H, 3‐H), 3.39 (dd, *J*_5,4_ = 8.9, *J*_5,6_ = 9.4 Hz, 1 H, 5‐H), 3.15 (t, 2 H, 13‐H), 2.94 (d, *J*_1,1′_ = 12.7 Hz, 1 H, 1‐H), 2.86 (t, 2 H, 8‐H), 2.82 (d, 1 H, 1′‐H) ppm. ^13^C NMR (75 MHz, D_2_O): *δ* = 97.0 (C‐2), 73.7, 72.3, 72.1 (3 C, C‐6, C‐4, C‐3), 69.6 (C‐5), 69.5, 69.2, 69.1, 67.6 (4 C, C‐12, C‐11, C‐10, C‐9), 60.7 (C‐7), 53.8 (C‐1), 48.2 (C‐8), 39.3 (C‐13) ppm.

***N*,*N′*‐Bis(1‐deoxy‐α‐d‐*gluco*‐hept‐2‐ulopyranosyl)‐4,7,10‐trioxa‐1,13‐tridecanediamine (8):** d‐*glycero*‐d‐*gulo*‐Heptopyranose (**3**; 300 mg, 1.43 mmol, 3 equiv.) was treated with 4,7,10‐trioxa‐1,13‐tridecanediamine (104 µL, 0.476 mmol, 1 equiv.) in EtOH (4 mL) and 1,4‐dioxane as co‐solvent in the presence of acetic acid (55 µL, 0.952 mmol, 2 equiv.) at 70 °C for 2 d. The solvents were removed under reduced pressure, and the crude product was passed through an ion exchange CG‐120‐II (Na+) Amberlite® resin column (H_2_O; H_2_O containing 1 % of conc. NH_4_OH) followed by purification by silica gel column chromatography (CHCl_3_/MeOH, 1:1, v/v containing 25 % of conc. NH_4_OH) to give disubstituted product **8** (168 mg, 0.278 mmol, 58 %). [*α*]_D_^20^ = +31.7 (*c* = 2.83, H_2_O). ^1^H NMR (300 MHz, D_2_O): *δ* = 3.85–3.62 (m,16 H, 12‐H, 11‐H, 7‐H, 7′‐H, 6‐H, 4‐H), 3.59 (t, 4 H, 10‐H), 3.42 (d, *J*_3,4_ = 9.3 Hz, 2 H, 3‐H), 3.38 (dd, *J*_5,4_ = 9.2, *J*_5,6_ = 9.4 Hz, 2 H, 5‐H), 2.98 (d, *J*_1,1′_ = 12.6 Hz, 2 H, 1‐H), 2.87 (d, 2 H, 1′‐H), 2.81 (t, 4 H, 8‐H), 1.82 (t, 4 H, 9‐H) ppm. ^13^C NMR (125 MHz, H_2_O/D_2_O, 9:1 v/v): *δ* = 95.4 (C‐2), 73.4, 72.6, 72.5 (3 C, C‐6, C‐4, C‐3), 69.5, 69.4, 69.3 (3 C, C‐12, C‐11, C‐10), 68.4 (C‐5), 60.6 (C‐7), 53.3 (C‐1), 46.6 (C‐8), 25.9 (C‐9) ppm. HRMS (MALDI): *m/z* calcd. for C_24_H_48_N_2_O_15_ [M + H]^+^ 605.3133; found 605.3134.

***N*,*N′*‐Bis(1‐deoxy‐α‐d‐*manno*‐hept‐2‐ulopyranosyl)‐1,6‐hexanediamine (10):** By applying general method A, d‐*glycero*‐d‐*galcato*‐heptopyranose (**9**; 204 mg, 0.971 mmol, 2 equiv.) was treated with 1,6‐diaminohexane (56 mg, 0.485 mmol, 1 equiv.) in EtOH (4 mL) and 1,4‐dioxane as co‐solvent in the presence of acetic acid (56 µL, 0.971 mmol, 2 equiv.) at 70 °C for 2 d. The solvents were removed under reduced pressure, and subsequent column chromatography (CHCl_3_/MeOH, 1:1, v/v containing 25 % of conc. NH_4_OH) gave disubstituted product **10** (146 mg, 0.292 mmol, 60 %). [*α*]_D_^20^ = +11.0 (*c* = 1.62, H_2_O). ^1^H NMR (300 MHz, D_2_O): *δ* = 3.98–3.86 (m, 10 H, 7‐H, 7′‐H, 6‐H, 4‐H, 3‐H), 3.66 (dd, *J*_5,4_ = 9.3, *J*_5,6_ = 9.5 Hz, 2 H, 5‐H), 3.42 (d, *J*_1,1′_ = 12.9 Hz, 2 H, 1‐H), 3.22 (d, 2 H, 1′‐H), 3.11 (t, 4 H, 8‐H), 1.74 (br. s, 4 H, 9‐H), 1.42 (br. s, 4 H, 10‐H) ppm. ^13^C NMR (75 MHz, D_2_O): *δ* = 95.1 (C‐2), 73.5, 72.1, 70.6 (3 C, C‐6, C‐4, C‐3), 66.1 (C‐5), 60.9 (C‐7), 53.2 (C‐1), 48.1 (C‐8), 25.2 (C‐9), 24.8 (C‐10) ppm. HRMS (MALDI): *m/z* calcd. for C_20_H_40_N_2_O_12_ [M + H]^+^ 501.2660; found 501.2664.

***N*,*N′*‐Bis(1‐deoxy‐α‐d‐*manno*‐hept‐2‐ulopyranosyl)‐*p*‐xylylenediamine (11):** By applying general method A, d‐*glycero*‐d‐*galacto*‐heptopyranose (**9**; 300 mg, 1.43 mmol, 2 equiv.) was treated with *p*‐xylylenediamine (97 mg, 0.714 mmol, 1 equiv.) in EtOH (5 mL) and 1,4‐dioxane as co‐solvent in the presence of acetic acid (82 µL, 1.43 mmol, 2 equiv.) at 70 °C for 2 d. The solvents were removed under reduced pressure, and the crude product was passed through an ion exchange CG‐120‐II (Na+) Amberlite® resin column (H_2_O; H_2_O containing 1 % of conc. NH_4_OH) followed by purification by silica gel column chromatography (CHCl_3_/MeOH, 1:1, v/v containing 25 % of conc. NH_4_OH) to give disubstituted product **11** (226 mg, 0.434 mmol, 61 %). [*α*]_D_^20^ = +14.1 (*c* = 3.4, H_2_O). ^1^H NMR (300 MHz, D_2_O): *δ* = 7.46–7.39 (br. s, 4 H, phenyl), 4.00 (br. s, 4 H, 8‐H), 3.93–3.80 (m, 6 H, 7‐H, 4‐H, 3‐H), 3.79–3.66 (m, 4 H, 7′‐H, 6‐H), 3.59 (dd, *J*_4,5_ = 9.5, *J*_5,6_ = 9.6 Hz, 2 H, 5‐H), 3.01 (d, *J*_1,1′_ = 13.4 Hz, 2 H, 1‐H), 2.97 (d, 2 H, 1′‐H) ppm. ^13^C NMR (125 MHz, H_2_O/D_2_O, 9:1 v/v): *δ* = 135.8, 129.3 (6 C, phenyl), 96.7 (C‐2), 73.1 (C‐6), 71.6, 70.9 (2 C, C‐4, C‐3), 66.6 (C‐5), 61.1 (C‐7), 53.1 (C‐1), 51.9 (C‐8) ppm. HRMS (MALDI): *m/z* calcd. for C_22_H_36_N_2_O_12_ [M + H]^+^ 521.2347; found 521.2346.

***N*,*N′*‐Bis(1‐deoxy‐α‐d‐*manno*‐hept‐2‐ulopyranosyl)‐2,2′‐ethylenedioxybis(ethylamine) (13):** By applying general method A, d‐*glycero*‐d‐*galacto*‐heptopyranose (**9**; 400 mg, 1.90 mmol, 2 equiv.) was treated with 2,2′‐ethylenedioxybis(ethylamine) (139 µL, 0.952 mmol, 1 equiv.) in EtOH (6 mL) and 1,4‐dioxane as co‐solvent in the presence of acetic acid (109 µL, 1.90 mmol, 2 equiv.) at 70 °C for 4 d. The solvents were removed under reduced pressure, and the crude product was passed through an ion exchange CG‐120‐II (Na+) Amberlite® resin column (H_2_O; H_2_O containing 1 % of conc. NH_4_OH) followed by purification by silica gel column chromatography (CHCl_3_/MeOH, 3:1, v/v containing 1 % of conc. NH_4_OH) to give disubstituted product **13** (148 mg, 0.278 mmol, 29 %) and monosusbtituted product **12** (178 mg, 0.523 mmol, 55 %).

**Compound 13:** [*α*]_D_^20^ = +11.5 (*c* = 1.73, H_2_O). ^1^H NMR (300 MHz, D_2_O): *δ* = 3.97–3.87 (m, 4 H, 4‐H, 3‐H), 3.86–3.67 (m, 14 H, 10‐H, 9‐H, 7‐H, 7′‐H, 6‐H), 3.64 (dd, *J*_5,4_ = 9.4, *J*_5,6_ = 9.5 Hz, 2 H, 5‐H), 3.39 (d, *J*_1,1′_ = 12.9 Hz, 2 H, 1‐H), 3.33–3.20 (m, 4 H, 8‐H), 3.24 (d, 2 H, 1′‐H) ppm. ^13^C NMR (75 MHz, D_2_O): *δ* = 97.0 (C‐2), 73.2, 71.5, 70.9 (3 C, C‐6, C‐4, C‐3), 69.5 (C‐10), 68.4 (C‐5), 66.7 (C‐9), 61.1 (C‐7), 53.7 (C‐1), 47.9 (C‐8) ppm. HRMS (MALDI): *m/z* calcd. for C_20_H_40_N_2_O_14_ [M + Na]^+^ 555.2377; found 555.2379.

**Compound 12:** ^1^H NMR (300 MHz, D_2_O): *δ* = 3.99–3.56 (m, 14 H, 12‐H, 11‐H, 10‐H, 9‐H, 7‐H, 7′‐H, 6‐H, 5‐H, 4‐H, 3‐H), 3.46 (d, *J*_1,1′_ = 13.0 Hz, 1 H, 1‐H), 3.27 (d, 1 H, 1′‐H), 3.38–3.15 (4 H, 13‐H, 8‐H) ppm. ^13^C NMR (75 MHz, D_2_O): *δ* = 94.9 (C‐2), 73.5, 72.3, 70.6 (3 C, C‐6, C‐4, C‐3), 66.1 (C‐5), 69.7, 66.4, 65.1 (4 C, C‐12, C‐11, C‐10, C‐9), 60.8 (C‐7), 53.3 (C‐1), 47.4 (C‐8), 39.1 (C‐13) ppm.

***N*,*N′*‐Bis(1‐deoxy‐α‐d‐*manno*‐hept‐2‐ulopyranosyl)‐4,7,10‐trioxa‐1,13‐tridecanediamine (14):** d‐*glycero*‐d‐*galacto*‐Heptopyranose (**9**; 448 mg, 2.13 mmol, 3 equiv.) was treated with 4,7,10‐trioxa‐1,13‐tridecanediamine (156 µL, 0.711 mmol, 1 equiv.) in EtOH (6 mL) and 1,4‐dioxane as co‐solvent in the presence of acetic acid (82 µL, 1.43 mmol, 2 equiv.) at 70 °C for 4 d. The solvents were removed under reduced pressure, and the crude product was passed through an ion exchange CG‐120‐II (Na+) Amberlite® resin column (H_2_O; H_2_O containing 1 % of conc. NH_4_OH) followed by purification by silica gel column chromatography (CHCl_3_/MeOH, 1:1, v/v containing 25 % of conc. NH_4_OH) to give disubstituted product **14** (234 mg, 0.387 mmol, 54 %). [*α*]_D_^20^ = +12.9 (*c* = 2.24, H_2_O). ^1^H NMR (300 MHz, D_2_O): *δ* = 3.94–3.83 (m, 6 H, 7‐H, 4‐H, 3‐H), 3.78–3.71 (m, 4 H, 7′‐H, 6‐H), 3.69–3.65 (s, 12 H, 12‐H, 11‐H, 10‐H), 3.62 (dd, 2 H, 5‐H), 3.13 (d, *J*_1,1′_ = 12.5 Hz, 2 H, 1‐H), 3.08 (d, 1 H, 1′‐H), 2.98 (t, 4 H, 8‐H), 1.90 (m, 4 H, 9‐H) ppm. ^13^C NMR (125 MHz, H_2_O/D_2_O, 9:1 v/v): *δ* = 96.5 (C‐2), 73.1 (C‐6), 71.8, 70.9 (2 C, C‐4, C‐3), 69.5, 69.3, 68.8 (3 C, C‐12, C‐11, C10), 66.6 (C‐5), 61.1 (C‐7), 53.9 (C‐1), 46.5 (C‐8), 26.9 (C‐9) ppm. HRMS (MALDI): *m/z* calcd. for C_24_H_48_N_2_O_15_ [M + H]^+^ 605.3133; found 605.3135.

**1‐[(4‐Aminobenzyl)amino]‐1‐deoxy‐α‐d‐*gluco*‐hept‐2‐ulose (16):** By applying general method B, d‐*glycero*‐d‐*gulo*‐heptopyranose (**3**; 300 mg, 1.43 mmol, 1. equiv.) was treated with (4‐aminobenzyl)amine (162 µL, 1.43 mmol, 1 equiv.) in EtOH (5 mL) and 1,4‐dioxane as co‐solvent in the presence of acetic acid (82 µL, 1.43 mmol, 1 equiv.) at 70 °C for 2 d. The solvents were removed under reduced pressure, and subsequent column chromatography (CHCl_3_/MeOH, 8:1, v/v containing 1 % of conc. NH_4_OH) gave product **16** (326 mg, 1.04 mmol, 73 %). [*α*]_D_^20^ = +37.6 (*c* = 1.82, MeOH). ^1^H NMR (300 MHz, [D_4_]MeOH): *δ* = 7.08 (d, 2 H, Ph), 6.69 (d, 2 H, Ph), 3.77 (dd, *J*_7,6_ = 1.9, *J*_7,7′_ = 10.9 Hz, 1 H, 7‐H), 3.75–3.59 (m, 5 H, 8‐H, 7′‐H, 6‐H, 4‐H), 3.29 (dd, *J*_5,4_ = 8.4, *J*_5,6_ = 9.3 Hz, 1 H, 5‐H), 3.27 (d, *J*_3,4_ = 9.3 Hz, 1 H, 3‐H), 2.81 (d, *J*_1,1′_ = 12.1 Hz, 1 H, 1‐H), 2.76 (d, 1 H, 1′‐H) ppm. ^13^C NMR (75 MHz, [D_4_]MeOH): *δ* = 147.8, 130.5, 129.9, 116.6 (6 C, Ph), 97.9 (C‐2), 75.7, 74.0 (2 C, C‐6, C‐4), 74.6 (C‐3), 71.7 (C‐5), 62.8 (C‐7), 55.3 (C‐1), 54.4 (C‐8) ppm. HRMS (ESI): *m/z* calcd. for C_14_H_22_N_2_O_6_ [M + H]^+^ 315.155; found 315.157.

**1‐[(4‐Aminobenzyl)amino]‐1‐deoxy‐α‐d‐*manno*‐hept‐2‐ulose (17):** By applying general method B, d‐*glycero*‐d‐*galacto*‐heptopyranose (**9**; 300 mg, 1.43 mmol, 1. equiv.) was treated with (4‐aminobenzyl)amine (162 µL, 1.43 mmol, 1 equiv.) in EtOH (5 mL) and 1,4‐dioxane as co‐solvent in the presence of acetic acid (82 µL, 1.43 mmol, 1 equiv.) at 70 °C for 2 d. The solvents were removed under reduced pressure, and subsequent column chromatography (CHCl_3_/MeOH, 8:1, v/v containing 1 % of conc. NH_4_OH) gave product **17** (327 mg, 1.04 mmol, 73 %). [*α*]_D_^20^ = +5.75 (*c* = 1.55, MeOH). ^1^H NMR (300 MHz, [D_4_]MeOH): *δ* = 7.08 (d, 2 H, phenyl), 6.69 (d, 2 H, phenyl), 3.85–3.75 (m, 3 H, 7‐H, 6‐H, 3‐H), 3.74–3.65 (m, 4 H, 8‐H, 7′‐H, 4‐H), 3.61 (dd, *J*_5,4_ = 9.1, *J*_5,6_ = 9.3 Hz, 1 H, 5‐H), 2.89 (d, *J*_1,1′_ = 12.2 Hz, 1 H, 1‐H), 2.79 (d, 1 H, 1′‐H) ppm. ^13^C NMR (75 MHz, [D_4_]MeOH): *δ* = 148.0, 130.6, 129.3, 116.6 (6 C, phenyl), 97.4 (C‐2), 74.9 (C‐3), 74.8 (C‐4), 73.1 (C‐6), 68.4 (C‐5), 63.0 (C‐7), 56.1 (C‐1), 54.1 (C‐8) ppm. HRMS (MALDI): *m/z* calcd. for C_14_H_22_N_2_O_6_ [M + H]^+^ 315.1556; found 315.1550.

**2‐(Benzyloxycarbonylamino)ethyl 3‐*O*‐(3‐Phthalimidopropyl)‐α‐d‐mannopyranoside (19):** To a solution of the benzyloxycarbonyl‐protected mannoside **18** 17 (3.29 g, 9.19 mmol) in anhydrous methanol (90 mL), dibutyltin oxide (2.40 g, 9.65 mmol) was added, and the solution was heated to reflux for 2 h. The solvent was evaporated, and the crude product was disolved in anhydrous *N*,*N*‐dimethylformamide (90 mL). *N*‐(3‐Bromopropyl)phthalimide (9.12 g, 34.0 mmol) and cesium fluoride (1.40 g, 9.19 mmol) were added, and the reaction mixture was stirred at room temperature for 3 d. The solvent was then evaporated, and purification of the crude product by column chromatography (CH_2_Cl_2_/EtOAc/MeOH, 10:5:1 → 5:5:1) gave **19** (1.87 g, 3.44 mmol, 37 %) as colorless foam. [*α*]_D_^23^ = +32.7 (*c* = 0.4 CH_2_Cl_2_). ^1^H NMR (500 MHz, [D_6_]DMSO): *δ* = 7.87–7.82 (m, 4 H, Ar‐H*_Phth_*), 7.37–7.29 (m, 6 H, NH, Ar‐H*_Cbz_*), 5.02 (s, 2 H, PhCH_2_O), 4.65 (s~d, 1 H, 1‐H), 3.82–3.80 (m, 1 H, 2‐H), 3.72–3.62 (m, 3 H, PhthNC*H*_2_, 6‐H), 3.61–3.56 (m, 2 H, C*H*_2_CH_2_NH), 3.51–3.48 (m, 1 H, 4‐H), 3.47–3.40 (m, 3 H, OC*H*_2_CH_2_CH_2_N, 6′‐H), 3.38–3.35 (m, 1 H, 5‐H), 3.27 (dd, ^3^ *J*_2,3_ = 3.0, ^3^ *J*_3,4_ = 9.2 Hz, 1 H, 3‐H), 3.24–3.14 (m, 2 H, C*H*_2_NH), 1.85 (p, ^3^ *J* = 6.7, ^3^ *J* = 6.7 Hz, 2 H, OCH_2_C*H*_2_CH_2_N) ppm. ^13^C NMR (126 MHz, [D_6_]DMSO): *δ* = 168.0 (2 C=O), 156.2 (OCONH), 137.2 (C‐Ar*_Phth_*), 134.3 (C‐Ar*_Phth_*), 131.7 (C‐Ar*_Cbz_*), 128.3 (C‐Ar*_Cbz_*), 127.7 (C‐Ar*_Cbz_*), 123.0 (C‐Ar*_Phth_*), 99.9 (C‐1), 79.6 (C‐3), 74.0 (C‐5), 66.8 (C‐2), 66.4 (*C*H_2_CH_2_NH), 65.7 (C‐4), 65.5 (O*C*H_2_CH_2_CH_2_N), 65.2 (Ph*C*H_2_O), 61.1 (C‐6), 40.1 (CH_2_NH), 35.1 (PhthNCH_2_), 28.7 (OCH_2_ *C*H_2_CH_2_N) ppm. IR (ATR): ν̃ = 3386, 2924, 1770, 1699, 1397, 1371, 1259, 1113, 1042, 721 cm^–1^. ESI‐MS: *m/z* calcd. for C_27_H_32_N_3_O_10_ 567.195 [M + Na]^+^; found 567.198.

**2‐(Benzyloxycarbonylamino)ethyl 3‐*O*‐(3‐Aminopropyl)‐α‐d‐mannopyranoside (20):** A solution of 2‐(benzyloxycarbonylamino)ethyl 3‐*O*‐(3‐phthalimidopropyl)‐α‐d‐mannopyranoside (**19**; 175 mg, 0.321 mmol) in MeOH (10 mL) was treated with hydrazine monohydrate (56 µL, 1.16 mmol, 3.6 equiv.), and the solution was stirred at 50 °C for 5 h. The reaction mixture was filtered through a bed of Celite, the solvent was removed under reduced pressure, and the residue was taken up in H_2_O, the pH was adjusted to 5 with 6 % aqueous HCl and the mixture extracted thoroughly with EtOAc. Removal of the solvent under reduced pressure and purification by column chromatography (CHCl_3_/MeOH, 6:1, v/v containing 1 % of conc. NH_4_OH) gave compound **20** (106 mg, 0.256 mmol, 80 %). ^1^H NMR (300 MHz, D_2_O): *δ* = 7.47–7.33 (5 H, Ph), 5.09 (br. s, 2 H, PhC*H*_2_O), 4.85 (dd, *J*_1,2_ = 1.65 Hz, 1 H, 1‐H), 4.05 (br. s, 1 H, 2‐H), 3.80 (dd, *J*_6,6′_ = 12.3, *J*_6,5_ = 2.3 Hz, 1 H, 6‐H), 3.78–3.45 (m, 8 H, OC*H*_2_CH_2_CH_2_NH_2_, C*H*_2_CH_2_NH, 6′‐H, 5‐H, 4‐H, 3‐H), 3.42–3.27 (br. s, 2 H, CH_2_C*H*_2_NH), 2.78–2.67 (br. s, 2 H, OCH_2_CH_2_C*H*_2_NH_2_), 1.74 (t, 2 H, OCH_2_C*H*_2_CH_2_NH_2_) ppm. ^13^C NMR (75 MHz, D_2_O): *δ* = 158.4 (C=O), 136.6, 128.8, 128.4, 127.6 (6 C, phenyl), 99.6 (C‐1), 78.7 (C‐3), 72.8 (C‐5), 67.4 (*C*H_2_CH_2_NH), 66.8 (Ph*C*H_2_O), 66.6 (C‐2), 66.3 (O*C*H_2_CH_2_CH_2_NH_2_), 65.6 (C‐4), 60.8 (C‐6), 40.1 (CH_2_ *C*H_2_NH), 37.8 (OCH_2_CH_2_ *C*H_2_NH_2_), 30.9 (OCH_2_ *C*H_2_CH_2_NH_2_) ppm.

**Propargyl 3‐*O*‐(3‐phthalimidopropyl)‐α‐d‐mannopyranoside (22):** To a solution of mannoside **21** 18 (327 mg, 1.50 mmol) in anhydrous methanol (13 mL), dibutyltin oxide (392 mg, 1.57 mmol) was added, and the solution was heated to reflux for 4 h. The solvent was then evaporated, and the crude product was desolved in anhydrous *N*,*N*‐dimethylformamide (13 mL). *N*‐(3‐Bromopropyl)phthalimide (1.49 g, 5.55 mmol) and cesium fluoride (229 mg, 1.50 mmol) were added, and the reaction mixture was stirred at room temperature for 3 d. The solvent was then evaporated, and purification of the crude product by column chromatography (dichloromethane/ethyl acetate, 1:1 → 1:3 → ethyl acetate/methanol, 10:1) gave **22** (182 mg, 450 µmol, 30 %) as a colorless foam. [*α*]_D_^23^ = +61.9 (*c* = 0.6 CH_2_Cl_2_). ^1^H NMR (500 MHz, [D_6_]DMSO): *δ* = 7.88–7.82 (m, 4 H, Ar‐H), 4.81 (d, ^3^ *J*_1,2_ = 1.6 Hz, 1 H, 1‐H), 4.77–4.75 (m, 2 H, OH), 4.49 (t, ^3^ *J* = 6.0 Hz, 1 H, OH), 4.24 (dd, ^4^ *J*_OC*H*CɡCH,CɡCH_ = 2.4, ^2^ *J*_OC*H*CɡCH,OCH′_ = 15.9 Hz, 1 H, OC*H*CɡCH), 4.16 (dd, ^4^ *J*_OC*H′*CɡCH,CɡCH_ = 2.4, ^2^ *J*_OC*H*CɡCH,OCH′_ = 15.9 Hz, 1 H, OCH′CɡCH), 3.82–3.81 (m, 1 H, 2‐H), 3.73–3.58 (m, 4 H, PhthNC*H*_2_, 6‐H, OC*H*CH_2_CH_2_N), 3.51–3.42 (m, 4 H, OC*H′*CH_2_CH_2_N, CɡCH, 4‐H, 6′‐H), 3.28 (ddd, ^3^ *J*_4,5_ = 8.8, ^3^ *J*_5,6_ = 6.3 Hz, ^3^ *J*_5,6′_ = 1.9 Hz, 1 H, 5‐H), 3.22 (dd, ^3^ *J*_2,3_ = 3.3, ^3^ *J*_3,4_ = 9.3 Hz, 1 H, 3‐H), 1.85 (p, ^3^ *J*_OCH2C*H2*CH2N,OC*H*2CH2CH2N_ = 6.8, ^3^ *J*_OCH2C*H2*CH2N,OCH2CH2C*H*2N_ = 6.8 Hz, 2 H, OCH_2_C*H*_2_CH_2_N) ppm. ^13^C NMR (150 MHz, [D_6_]DMSO): *δ* = 168.5 (C=O), 134.8, 132.2, 123.5 (C‐Ar), 98.5 (C‐1), 80.2 (*C*ɡCH), 80.0 (C‐3), 77.8 (Cɡ*C*H), 74.9 (C‐5), 67.1 (C‐2), 67.0 (O*C*H_2_CH_2_CH_2_N), 66.1 (C‐4), 61.5 (C‐6), 53.42 (O*C*H_2_CɡCH), 35.5 (PhthN*C*H_2_), 29.2 (OCH_2_ *C*H_2_CH_2_N) ppm. IR (ATR): ν̃ = 3464, 3279, 2923, 1769, 1700, 1396, 1371, 1113, 1039, 964, 720 cm^–1^. ESI‐MS: *m/z* calcd. for C_20_H_23_NO_8_ 428.132 [M + Na]^+^; found 428.133.

**Propargyl 3‐*O*‐(3‐Aminopropyl)‐α‐d‐mannopyranoside (23):** A solution of propargyl 3‐*O*‐(3‐phthalimidopropyl)‐α‐d‐mannopyranoside (**22**; 200 mg, 0.493 mmol) in MeOH (10 mL) was treated with hydrazine monohydrate (89 µL, 1.83 mmol, 3.7 equiv.), and the solution was stirred at 50 °C for 5 h. The reaction mixture was filtered through a bed of Celite, the solvent was removed under reduced pressure, and the residue was taken up in H_2_O, the pH was adjusted to 5 with 6 % aqueous HCl, and the mixture extracted thoroughly with EtOAc. Removal of the solvent under reduced pressure and purification by column chromatography (CHCl_3_/MeOH, 6:1, v/v containing 1 % of conc. NH_4_OH) gave compound **23** (121 mg, 0.440 mmol, 89 %). ^1^H NMR (300 MHz, D_2_O): *δ* = 5.06 (d, 1 H, 1‐H), 4.32 (dd, 2 H, OC*H*_2_CɡCH), 4.15 (dd, *J*_2,3_ = 3.3, *J*_2,1_ = 1.9 Hz, 1 H, 2‐H), 3.88 (dd, *J*_6,6′_ = 12.1, *J*_6,5_ = 1.9 Hz, 1 H, 6‐H), 3.84–3.61 (m, 6 H, OC*H*_2_CH_2_CH_2_NH_2_, OCH_2_CɡC*H*, 6′‐H, 5‐H, 4‐H), 3.58 (dd, *J*_3,4_ = 8.3, *J*_3,2_ = 3.2 Hz, 1 H, 3‐H), 2.93 (t, 2 H, OCH_2_CH_2_C*H*_2_NH_2_), 1.91–1.81 (m, 2 H, OCH_2_C*H*_2_CH_2_NH_2_) ppm. ^13^C NMR (75 MHz, D_2_O): *δ* = 98.7 (C‐1), 78.6 (C‐3), 78.4 (Cɡ*C*H), 73.2 (C‐5), 67.5 (O*C*H_2_CH_2_CH_2_NH_2_), 66.5 (C‐2), 65.5 (C‐4), 60.8 (C‐6), 54.6 (O*C*H_2_CɡCH), 37.9 (OCH_2_CH_2_ *C*H_2_NH_2_), 29.2 (OCH_2_ *C*H_2_CH_2_NH_2_) ppm.

**1‐{[2‐(α‐d‐Mannopyranosyl)ethyl]amino}‐1‐deoxy‐α‐d‐*gluco*‐hept‐2‐ulose (25):** By applying general method B, d‐*glycero*‐d‐*gulo*‐heptopyranose (**3**; 105 mg, 0.500 mmol, 1.1 equiv.) was treated with 2‐aminoethyl α‐d‐mannopyranoside^19^ (**24**; 100 mg, 0.448 mmol, 1 equiv.) in EtOH (4 mL) and 1,4‐dioxane as co‐solvent in the presence of acetic acid (26 µL, 0.448 mmol, 1 equiv.) at 70 °C for 4 d. The solvents were removed under reduced pressure, and subsequent column chromatography (CHCl_3_/MeOH, 1:1 v/v containing 25 % of conc. NH_4_OH) gave product **25** (84 mg, 0.202 mmol, 45 %). [*α*]_D_^20^ = +46.8 (*c* = 1.15, H_2_O). ^1^H NMR (300 MHz, D_2_O): *δ* = 4.88 (d, *J*_10,11_ = 1.8 Hz, 1 H, 10‐H), 4.02–3.57 (m, 12 H, 15‐H, 15′‐H, 14‐H, 13‐H, 12‐H, 11‐H; 9‐H, 7‐H, 7′‐H, 6‐H, 4‐H), 3.46 (d,* J*_3,4_ = 9.6 Hz, 1 H, 3‐H), 3.41 (dd, *J*_5,4_ = 9.5, *J*_5,6_ = 9.6 Hz, 1 H, 5‐H), 3.21 (d, 1 H, 1‐H), 3.25–3.16 (m, 2 H, 8‐H), 3.17 (d, *J*_1,1′_ = 12.0 Hz, 1 H, 1′‐H) ppm. ^13^C NMR (75 MHz, D_2_O): *δ* = 100.0 (C‐10), 95.5 (C‐2), 73.4, 73.0 (2 C, C‐6, C‐4), 72.7 (C‐14), 72.6 (C‐3), 70.5, 69.9 (2 C, C‐13, C‐11), 69.4 (C‐5), 66.7 (C‐12), 63.3 (C‐9), 60.9, 60.6 (2 C, C‐15, C‐7), 53.1 (C‐1), 47.6 (C‐8) ppm. HRMS (MALDI): *m/z* calcd. for C_15_H_29_NO_12_ [M + H]^+^ 416.1768; found 416.1768.

***N*‐[(α‐d‐Mannopyranosyl)ethyl]amino‐1‐deoxy‐α‐d‐*manno*‐hept‐2‐ulose (26):** By applying general method B, d‐*glycero*‐d‐*galacto*‐heptopyranose (**9**; 94 mg, 0.448 mmol, 1 equiv.) was treated with 2‐aminoethyl α‐d‐mannopyranoside^19^ (**24**; 100 mg, 0.448 mmol, 1 equiv.) in EtOH (4 mL) and 1,4‐dioxane as co‐solvent in the presence of acetic acid (26 µL, 0.448 mmol, 1 equiv.) at 70 °C for 4 d. The solvents were removed under reduced pressure, and subsequent column chromatography (CHCl_3_/MeOH, 1:1, v/v containing 25 % of conc. NH_4_OH) gave product **26** (120 mg, 0.289 mmol, 65 %). [*α*]_D_^20^ = +33.9 (*c* = 1.36, H_2_O). ^1^H NMR (300 MHz, D_2_O): *δ* = 4.89 (br. s, 1 H, 10‐H), 4.05–3.69 (m, 11 H, 15‐H, 15′‐H, 13‐H, 11‐H, 9‐H, 7‐H, 7′‐H, 6‐H, 4‐H, 3‐H), 3.68–3.54 (m, 3 H, 14‐H, 12‐H; 5‐H), 3.46 (d, *J*_1,1′_ = 12.9 Hz, 1 H, 1‐H), 3.37 (t, 2 H, 8‐H), 3.27 (d, 1 H, 1′‐H) ppm. ^13^C NMR (75 MHz, D_2_O): *δ* = 99.9 (C‐10), 96.5 (C‐2), 73.2, 71.9 (2 C, C‐4, C‐3), 72.9, 70.9 (2 C, C‐13, C‐12), 70.5 (C‐6), 69.9 (C‐11), 66.7, 66.5 (2 C, C‐14, C‐5), 64.7 (C‐9), 61.0, 60.9 (2 C, C‐15, C‐7), 53.7 (C‐1), 47.7 (C‐8) ppm. HRMS (MALDI): *m/z* calcd. for C_15_H_29_NO_12_ [M + H]^+^ 417.1846; found 417.1807.

**1‐{[2‐(Benzyloxycarbonylamino)ethyl α‐d‐mannopyranoside‐3‐*O*‐yl)propyl]amino}‐1‐deoxy‐α‐d‐*manno*‐hept‐2‐ulose (27):** By applying general method B, d‐*glycero*‐d‐*galacto*‐heptopyranose (**9**; 222 mg, 1.06 mmol, 1 equiv.) was treated with 2‐(benzyloxycarbonylamnio)ethyl 3‐*O*‐(3‐aminopropyl)‐α‐d‐mannopyranoside (**20**; 436 mg, 1.05 mmol, 1 equiv.) in EtOH (5 mL) and 1,4‐dioxane as co‐solvent in the presence of acetic acid (60 µL, 1.06 mmol, 1 equiv.) at 70 °C for 5 d. The solvents were removed under reduced pressure, and subsequent column chromatography (CHCl_3_/MeOH, 1:1, v/v containing 1 % of conc. NH_4_OH) gave product **27** (250 mg, 0.412 mmol, 39 %). [*α*]_D_^20^ = +18.6 (*c* = 1.95, H_2_O). ^1^H NMR (300 MHz, D_2_O, pH = 7 H/D exchange; pH = 8): *δ* = 7.39–7.27 (s, 5 H, Ph), 5.00 (dd, 2 H, 20‐H), 4.87 (br. s, 1 H, 11‐H, pH = 7), 4.01 (s, 1 H, 12‐H), 3.95–3.45 (m, 15 H, 17‐H, 16‐H, 16′‐H, 15‐H, 14‐H, 13‐H, 10‐H, 7‐H, 7′‐H, 6‐H, 5‐H, 4‐H, 3‐H), 3.28 (q, 2 H, 18‐H), 3.13 (d,* J*_1,1′_ = 12.8 Hz, 1 H, 1 H, 1‐H), 3.04 (d,1 H, 1′‐H), 2.99 (t, 2 H, 8‐H), 2.00–1.74 (m, 2 H, 9‐H) ppm. ^13^C NMR (125 MHz, H_2_O/D_2_O, 9:1, v/v; pH 8 signals for C‐1 detectable): *δ* = 158.4 (C=O), 136.5, 128.8, 128.4, 127.6 (6 C, Ph), 99.6 (C‐11), 95.6 (C‐2), 78.9 (C‐13), 73.3, 72.1 (2 C, C‐4, C‐3), 72.7 (C‐15), 70.7 (C‐6), 67.5, 66.8, 66.4 (3 C, C‐20, C‐17, C‐10), 66.5 (C‐12), 66.3 (C‐14), 65.5 (C‐5), 60.9, 60.7 (2 C, C‐16, C‐7), 53.7 (C‐1), 47.1 (C‐8), 40.1 (C‐18), 25.9 (C‐9) ppm. HRMS (MALDI): *m/z* calcd. for C_26_H_42_N_2_O_14_ [M + H]^+^ 607.2714; found 607.2717.

**1‐{[(Propargyl β‐d‐mannopyranoside‐3‐*O*‐yl)propyl]amino}‐1‐deoxy‐α‐d‐*manno*‐hept‐2‐ulose (28):** By applying general method B, d‐*glycero*‐d‐*galacto*‐heptopyranose (**9**; 178 mg, 0.847 mmol, 1.2 equiv.) was treated with propargyl 3‐*O*‐(3‐aminopropyl)‐α‐d‐mannopyranoside (**23**; 190 mg, 0.690 mmol, 1 equiv.) in EtOH (4 mL) and 1,4‐dioxane as co‐solvent in the presence of acetic acid (48 µL, 0.847 mmol, 1.2 equiv.) at 70 °C for 5 d. The solvents were removed under reduced pressure, and subsequent column chromatography (CHCl_3_/MeOH, 2:1, v/v containing 1 % of conc. NH_4_OH) gave product **28** (81 mg, 0.173 mmol, 25 %). [*α*]_D_^20^ = +37.9 (*c* = 0.912, H_2_O). ^1^H NMR (300 MHz, D_2_O): *δ* = 5.04 (br. s, 1 H, 11‐H), 4.31 (dd, 2 H, 17‐H), 4.13 (br. s, 1 H, 12‐H), 3.94–3.59 (m, 12 H, 16‐H, 16′‐H, 15‐H, 14‐H; 10‐H, 7‐H, 7′‐H, 6‐H, 5‐H, 4‐H, 3‐H), 3.56 (dd, *J*_13,12_ = 3.5, *J*_13,14_ = 9.2 Hz, 1 H, 13‐H), 3.17–2.88 (m, 4 H, 1‐H, 1′‐H, 8‐H), 1.99–1.86 (m, 2 H, 9‐H) ppm. ^13^C NMR (125 MHz, H_2_O/D_2_O, 9:1 v/v): *δ* = 98.6 (C‐11), 95.0 (C‐2), 78.7 (C‐13), 78.6 (C‐18), 76. 1 (C‐19), 73.4, 73.0, 72.1, 70.6, 67.3, 66.4, 66.1, 65.5 (8 C, C‐15, C‐14, C‐12, C‐10, C‐6, C‐5, C‐4, C‐3), 60.8, 60.7 (2 C, C‐16, C‐7), 54.5 (C‐17), 53.6 (C‐1), 47.2 (C‐8), 25.2 (C‐9) ppm. HRMS (MALDI): *m/z* calcd. for C_19_H_33_NO_12_ [M + H]^+^ 468.2081; found 468.2085.

**1‐{[(5*S*)‐5‐(*tert*‐Butoxycarbonylamino)‐5‐(hydroxycarbonyl)pentyl]amino}‐1‐deoxy‐α‐d‐*manno*‐hept‐2‐ulose (30):** By applying general method B, d‐*glycero*‐d‐*galacto*‐heptopyranose (**9**; 200 mg, 0.952 mmol, 1. equiv.) was treated with *N^α^*‐(*tert*‐butoxycarbonyl)‐l‐lysine (**29**; 234 mg, 0.950 mmol, 1 equiv.) in EtOH (4 mL) and 1,4‐dioxane as co‐solvent in the presence of acetic acid (55 µL, 0.952 mmol, 1 equiv.) at 70 °C for 4 d. The solvents were removed under reduced pressure, and subsequent column chromatography (CHCl_3_/MeOH, 6:1, v/v containing 1 % of conc. NH_4_OH) gave product **30** (263 mg, 0.600 mmol, 63 %). [*α*]_D_^20^ = +21.8 (*c* = 2.87, MeOH). ^1^H NMR (300 MHz, [D_4_]MeOH): *δ* = 3.87 (t, 1 H, 12‐H), 3.82–3.73 (m, 3 H, 7‐H, 6‐H, 3‐H), 3.72–3.59 (m, 2 H, 7′‐H, 4‐H), 3.57 (dd, *J*_5,4_ = 8.5, *J*_5,6_ = 9.5 Hz, 1 H, 5‐H), 3.25 (d, 1 H, 1‐H), 3.07 (d, *J*_1,1′_ = 12.7 Hz, 1 H, 1′‐H), 2.93 (t, 2 H, 8‐H), 1.79–1.51 (m, 4 H, 9‐H, 11‐H), 1.41–1.27 (br. s, 11 H, 10‐H, Boc) ppm. ^13^C NMR (75 MHz, [D_4_]MeOH): *δ* = 179.5 (C‐13), 157.6 (C‐14), 96.1 (C‐2), 80.1 (C‐15), 75.1 (C‐4), 74.6 (C‐3), 72.5 (C‐6), 67.8 (C‐5), 62.5 (C‐7), 56.7 (C‐12), 54.9 (C‐1), 49.2 (C‐8), 33.6 (C‐11), 28.8 (Boc), 26.3 (C‐9), 23.6 (C‐10) ppm. HRMS (MALDI): *m/z* calcd. for C_18_H_34_N_2_O_10_ [M + H]^+^ 439.2292; found 439.2293.

**1‐{[(5*S*)‐5‐(Benzyloxycarbonylamino)‐5‐(methoxycarbonyl)pentyl]amino}‐1‐deoxy‐α‐d‐*manno*‐hept‐2‐ulose (32):** By applying general method B, d‐*glycero*‐d‐*galacto*‐heptopyranose (**9**; 200 mg, 0.952 mmol, 1. equiv.) was treated with *N^α^*‐(benzyloxycarbonyl)‐l‐lysine (**31**; 267 mg, 0.952 mmol, 1 equiv.) in EtOH (4 mL) and 1,4‐dioxane as co‐solvent in the presence of acetic acid (55 µL, 0.952 mmol, 1 equiv.) at 70 °C for 4 d. The solvents were removed under reduced pressure, and subsequent column chromatography (CHCl_3_/MeOH, 6:1, v/v containing 1 % of conc. NH_4_OH) gave product **32** (292 mg, 0.618 mmol, 65 %). [*α*]_D_^20^ = +12.2 (*c* = 0.545, MeOH). ^1^H NMR (300 MHz, [D_4_]MeOH): *δ* = 7.38–7.23 (m, 5 H, Ph), 5.05 (br. s, 2 H, 15‐H), 4.07–3.96 (m, 1 H, 12‐H), 3.86 (d,* J*_3,4_ = 3.2 Hz, 1 H, 3‐H), 3.86–3.73 (m, 3 H, 7‐H, 7′‐H, 6‐H), 3.72–3.66 (m, 1 H, 4‐H), 3.65 (dd, *J*_5,4_ = 8.5, *J*_5,6_ = 9.4 Hz, 1 H, 5‐H), 3.29 (d, 1 H, 1‐H), 3.09 (d, *J*_1,1′_ = 12.2 Hz, 1 H, 1′‐H), 2.97 (t, 2 H, 8‐H), 1.91–1.59 (m, 4 H, 9‐H, 11‐H), 1.42 (q, 2 H, 10‐H) ppm. ^13^C NMR (75 MHz, [D_4_]MeOH): *δ* = 179.1 (C‐13), 158.1 (C‐14), 138.4, 129.5, 128.9, 128.8 (6 C, Ph), 96.1 (C‐2), 75.1 (C‐4), 74.8 (C‐3), 72.6 (C‐6), 67.8 (C‐5), 67.4 (C‐15), 62.5 (C‐7), 57.2 (C‐12), 55.1 (C‐1), 49.2 (C‐8), 33.5 (C‐11), 26.4 (C‐9), 23.5 (C‐10) ppm. HRMS (MALDI): *m/z* calcd. for C_21_H_32_N_2_O_10_ [M + H]^+^ 473.2135; found 473.2137.

**1‐{[(5*S*)‐5‐(*tert*‐Butoxycarbonylamino)‐5‐(methoxycarbonyl)pentyl]amino}‐1‐deoxy‐α‐d‐*manno*‐hept‐2‐ulose (34):** By applying general method B, d‐*glycero*‐d‐*galacto*‐heptopyranose (**9**; 300 mg, 1.43 mmol, 1. equiv.) was treated with methyl *N^α^*‐(*tert*‐butoxycarbonyl)‐l‐lysinate (**33**; 370 mg, 1.42 mmol, 1 equiv.) in EtOH (5 mL) and 1,4‐dioxane as co‐solvent in the presence of acetic acid (82 µL, 1.43 mmol, 1 equiv.) at 70 °C for 3 d. The solvents were removed under reduced pressure, and subsequent column chromatography (CHCl_3_/MeOH, 6:1, v/v containing 1 % of conc. NH_4_OH) gave product **34** (438 mg, 0.968 mmol, 68 %). [*α*]_D_^20^ = +0.63 (*c* = 2.06, MeOH). ^1^H NMR (300 MHz, [D_4_]MeOH): *δ* = 4.15–4.05 (m, 1 H, 12‐H), 3.89 (d, *J*_3,4_ = 3.3 Hz, 1 H, 3‐H), 3.87–3.77 (m, 3 H, 7‐H, 7′‐H, 6‐H), 3.76–3.67 (m, 4 H, 4‐H, OCH_3_), 3.66 (dd, *J*_4,5_ = 9.0, *J*_5,6_ = 9.3 Hz, 1 H, 5‐H), 3.37 (d, *J*_1,1′_ = 12.3 Hz, 1 H, 1‐H), 3.15 (d, 1 H, 1′‐H), 3.06 (t, 2 H, 8‐H), 1.91–1.59 (m, 4 H, 9‐H, 11‐H), 1.53–1.33 (br. s, 11 H, 10‐H, Boc) ppm. ^13^C NMR (75 MHz, [D_4_]MeOH): *δ* = 174.7 (C‐13), 158.1 (C‐14), 95.9 (C‐2), 80.7 (C‐15), 75.1 (C‐4), 74.8 (C‐3), 72.4 (C‐6), 67.6 (C‐5), 62.3 (C‐7), 55.1 (C‐1), 54.8 (C‐12), 52.7 (OCH_3_), 49.2 (C‐8), 32.0 (C‐11), 28.7 (Boc), 26.2 (C‐9), 23.9 (C‐10) ppm. HRMS (MALDI): *m/z* calcd. for C_19_H_36_N_2_O_10_ [M + H]^+^ 453.2448; found 453.2448.

**Methyl *N^α^*‐(*tert*‐Butoxycarbonyl)‐l‐lysinyl‐*N^6^*‐(1‐deoxy‐α‐d‐*gluco*‐hept‐2‐ulose)‐l‐alaninate (36):** By applying general method B, d‐*glycero*‐d‐*gluco*‐heptopyranose (**3**; 67 mg, 0.319 mmol, 1.3 equiv.) was treated with methyl *N^α^*‐(*tert*‐butoxycarbonyl)‐l‐lysinyl‐l‐anlaninate[20a] (**35**; 93 mg, 0.281 mmol, 1 equiv.) in EtOH (10 mL) and 1,4‐dioxane as co‐solvent in the presence of acetic acid (18 µL, 0.319 mmol, 1 equiv.) at 70 °C for 5 h. The solvents were removed under reduced pressure, and subsequent column chromatography (CHCl_3_/MeOH, 3:1, v/v containing 1 % of conc. NH_4_OH) gave product **36** (119 mg, 0.227 mmol, 81 %). ^1^H NMR (300 MHz, [D_4_]MeOH): *δ* = 4.29 (q, 1 H, 16‐H), 3.93 (t, 1 H, 12‐H), 3.68–3.44 (m, 7 H, 7‐H, 7′‐H, 6‐H, 4‐H, OCH_3_), 3.15–3.06 (dd, 2 H, 5‐H, 3‐H), 3.12 (d, *J*_1,1′_ = 12.7 Hz, 1 H, 1‐H), 3.05 (d, 1 H, 1′‐H), 2.87–2‐75 (m, 2 H, 8‐H), 1.63–1.48 (m, 3 H, 9‐H, 11‐H), 1.49–1.38 (m, 1 H, 11‐H), 1.33–1.21 (br. s, 11 H, 10‐H, Boc), 1.19 (d, 3 H, 18‐H) ppm. ^13^C NMR (75 MHz, [D_4_]MeOH): *δ* = 174.7, 174.5 (2 C, C‐17, C‐13), 157.7 (C‐14), 96.2 (C‐2), 80.6 (C‐15), 75.1, 74.6, 74.5 (3 C, C‐6, C‐4, C‐3), 71.4 (C‐5), 62.5 (C‐7), 55.3 (C‐1), 54.4 (C‐12), 52.8 (OCH_3_), 49.9 (C‐8), 49.4 (C‐16), 32.8 (C‐11), 28.7 (Boc), 26.5 (C‐9), 23.8 (C‐10), 17.4 (C‐18) ppm.

**Methyl *N^α^*‐(*tert*‐Butoxycarbonyl)‐l‐lysinyl‐*N^6^*‐(1‐deoxy‐α‐d‐*manno*‐hept‐2‐ulose)‐l‐alaninate (37):** By applying general method B, d‐*glycero*‐d‐*galacto*‐heptopyranose (**9**; 150 mg, 0.714 mmol, 1. equiv.) was treated with methyl *N^α^*‐(*tert*‐butoxycarbonyl)‐l‐lysinyl‐l‐anlaninate[20a] (**35**; 237 mg, 0.715 mmol, 1 equiv.) in EtOH (3 mL) and 1,4‐dioxane as co‐solvent in the presence of acetic acid (41 µL, 0.714 mmol, 1 equiv.) at 70 °C for 3 d. The solvents were removed under reduced pressure, and subsequent column chromatography (CHCl_3_/MeOH, 6:1, v/v containing 1 % of concd. NH_4_OH) gave product **37** (231 mg, 0.441 mmol, 62 %). [*α*]_D_^20^ = –12.3 (*c* = 0.86, MeOH). ^1^H NMR (300 MHz, [D_4_]MeOH): *δ* = 4.42 (q, 1 H, 16‐H), 4.10–4.00 (m, 1 H, 12‐H), 3.87–3.80 (m, 3 H, 7‐H, 6‐H, 3‐H), 3.79–3.68 (m, 5 H, 7′‐H, 4‐H, OCH_3_), 3.64 (dd, *J*_4,5_ = 8.9, *J*_5,6_ = 9.5 Hz, 1 H, 5‐H), 3.20 (d, *J*_1,1′_ = 12.5 Hz, 1 H, 1‐H), 3.02 (d, 1 H, 1′‐H), 2.92 (t, 2 H, 8‐H), 1.84–1.56 (m, 4 H, 9‐H, 11‐H), 1.52–1.35 (m, 14 H, 18‐H, 10‐H, Boc) ppm. ^13^C NMR (75 MHz, [D_4_]MeOH): *δ* = 174.7, 174.6 (2 C, C‐17, C‐13), 157.8 (C‐14), 96.4 (C‐2), 80.7 (C‐15), 75.0, 74.9 (2 C, C‐4, C‐3), 72.7 (C‐6), 67.9 (C‐5), 62.6 (C‐7), 55.8 (C‐1), 55.4 (C‐12), 52.8 (OCH_3_), 49.4 (C‐8), 49.2 (C‐16), 32.9 (C‐11), 28.7 (Boc), 27.5 (C‐9), 23.9 (C‐10), 17.4 (C‐18) ppm. HRMS (MALDI): *m/z* calcd. for C_22_H_41_N_3_O_11_ [M + H]^+^ 524.2819; found 524.2819.

**Methyl *N*‐(*tert*‐Butoxycarbonyl)‐l‐prolinyl‐*N^6^*‐(1‐deoxy‐α‐d‐*gluco*‐hept‐2‐ulose)‐l‐lysinyl‐l‐alaninate (39):** By applying general method B, d‐*glycero*‐d‐*gulo*‐heptopyranose (**3**; 340 mg, 1.62 mmol, 1. equiv.) was treated with methyl *N*‐(*tert*‐butoxycarbonyl)‐l‐prolyl‐l‐lysyl‐l‐alaninate[20a], [20b] (**38**; 693 mg, 1.62 mmol, 1 equiv.) in EtOH (5 mL) and 1,4‐dioxane as co‐solvent in the presence of acetic acid (93 µL, 1.62 mmol, 1 equiv.) at 70 °C for 4 d. The solvents were removed under reduced pressure, and subsequent column chromatography (CHCl_3_/MeOH, 3:1, v/v containing 1 % of conc. NH_4_OH) gave product **39** (392 mg, 0.632 mmol, 39 %). [*α*]_D_^20^ = –29.1 (*c* = 3.65, MeOH). ^1^H NMR (300 MHz, [D_4_]MeOH): *δ* = 4.46–4.32 (m, 2 H, 18‐H, 14‐H), 4.28–4.19 (m, 1 H, 12‐H), 3.84–3.60 (m, 4 H, 7‐H, 7′‐H, 6‐H, 4‐H), 3.72 (s, 3 H, OCH_3_), 3.56–3.38 (m, 2 H, 21‐H), 3.31 (dd, *J*_4,5_ = 9.6, *J*_5,6_ = 9.6 Hz, 1 H, 5‐H), 3.29 (d, *J*_3,4_ = 9.6 Hz, 1 H, 3‐H), 2.86 (d, *J*_1,1′_ = 12.3 Hz, 1 H, 1‐H), 2.78 (d, 1 H, 1′‐H), 2.74–2.56 (m, 2 H, 8‐H), 2.34–2.12 (m, 1 H, 11‐H), 2.04–1.65 (m, 5 H, 19‐H, 11‐H, 9‐H), 1.64–1.36 (m, 16 H, 20‐H, 16‐H, 10‐H, Boc) ppm. ^13^C NMR (75 MHz, [D_4_]MeOH, particular peaks in the peptide part were found in doublets due to rotameric appearance): *δ* = 175.4, 175.2, 174.5, 174.0, 173.9 (5 C, 2 × C‐17, C‐15, 2 × C‐13), 156.5, 156.0 (2 C, 2 × C‐22), 97.9 (C‐2), 81.4, 81.3 (2 C, 2 × C‐23), 75.7 (C‐4), 74.5 (C‐3), 74.0 (C‐6), 71.7 (C‐5), 62.8 (C‐7), 61.4, 61.3 (2 C, 2 × C‐12), 56.1 (C‐1), 54.4 (C‐18), 52.8 (OMe), 50.8 (C‐8), 49.4 (C‐14), 47.9 (C‐21), 33.4, 33.0 (2X C‐11), 32.5, 31.4 (2 × C‐9), 30.3, 30.2 (2 × C‐20), 28.8 (Boc), 25.5, 24.7 (2 × C‐19), 24.5, 24.3 (2 × C‐10), 17.4 (C‐16) ppm. HRMS (MALDI): *m/z* calcd. for C_27_H_48_N_4_O_12_ [M + H]^+^ 621.3347; found 621.3348.

**Methyl *N*‐(*tert*‐Butoxycarbonyl)‐l‐prolinyl‐*N^6^*‐(1‐deoxy‐α‐d‐*manno*‐hept‐2‐ulose)‐l‐lysinyl‐l‐alaninate (40):** By applying general method B, d‐*glycero*‐d‐*galacto*‐heptopyranose (**9**; 165 mg, 0.785 mmol, 1. equiv.) was treated with methyl *N*‐(*tert*‐butoxycarbonyl)‐l‐prolyl‐l‐lysyl‐l‐alaninate[20a], [20b] (**38**; 335 mg, 0.782 mmol, 1 equiv.) in EtOH (4 mL) and 1,4‐dioxane as co‐solvent in the presence of acetic acid (45 µL, 0.785 mmol, 1 equiv.) at 70 °C for 4 d. The solvents were removed under reduced pressure, and subsequent column chromatography (CHCl_3_/MeOH, 3:1, v/v containing 1 % of conc. NH_4_OH) gave product **40** (239 mg, 0.385 mmol, 49 %). [*α*]_D_^20^ = –44.7 (*c* = 2.62, MeOH). ^1^H NMR (300 MHz, [D_4_]MeOH): *δ* = 4.44–4.30 (m, 2 H, 18‐H, 14‐H), 4.25–4.18 (m, 1 H, 12‐H), 3.87–3.79 (m, 3 H, 7‐H, 6‐H, 3‐H), 3.79–3.67 (m, 2 H, 7′‐H, 4‐H), 3.70 (s, 3 H, OCH_3_), 3.63 (dd, *J*_4,5_ = 8.7, *J*_5,6_ = 9.0 Hz, 1 H, 5‐H), 3.53–3.34 (m, 2 H, 21‐H), 3.31 (d, 1 H, 1‐H), 3.09 (d, *J*_1,1′_ = 12.5 Hz, 1 H, 1‐H′), 2.99 (t, 2 H, 8‐H), 2.31–2.11 (m, 1 H, 11‐H), 2.01–1.63 (m, 7 H, 20‐H, 19‐H, 11‐H, 9‐H), 1.56–1.33 (m, 14 H, 16‐H, 10‐H, Boc) ppm. ^13^C NMR (75 MHz, [D_4_]MeOH, particular peaks in the peptide part were found in doublets due to rotameric appearance): *δ* = 175.5, 175.3, 174.5, 173.8, 173.6 (5 C, 2X C‐17, C‐15, 2 × C‐13), 156.4, 155.9 (2 C, 2 × C‐22), 96.0 (C‐2), 81.4, 81.3 (2 C, 2 × C‐23), 75.1 (C‐4), 74.6 (C‐3), 72.4 (C‐6), 67.7 (C‐5), 62.4 (C‐7), 61.4, 61.3 (2 C, 2 × C‐12), 55.0 (C‐1), 54.2, 54.1 (2C. 2 × C‐18), 52.8 (OMe), 49.4 (C‐14), 49.1 (C‐8), 47.9 (C‐21), 32.8, 31.6 (2 × C‐11), 32.5 (C‐9), 28.8 (Boc), 26.4, 26.3 (2 × C‐19), 25.5, 24.7 (2 × C‐20), 23.8, 23.7 (2 × C‐10), 17.3 (C‐16) ppm. HRMS (MALDI): *m/z* calcd. for C_27_H_48_N_4_O_12_ [M + H]^+^ 621.3347, found 621.3349.

## Supporting information

Supporting Information
