# Supplementary material for: The Amadori Rearrangement for Carbohydrate Conjugation: Scope and Limitations
Source: European J Org Chem. 2016 Jun 27;2016(25):4328–37. doi: 10.1002/ejoc.201600458 (PMC5094532; doi:10.1002/ejoc.201600458)
Supplement: Supplementary file 1 — Supporting Information [file EJOC-2016-4328-s001.pdf]

Eur. J. Org. Chem. **2016** · ISSN 1099–0690

SUPPORTING INFORMATION

DOI: 10.1002/ejoc.201600458
Title: The Amadori Rearrangement for Carbohydrate Conjugation: Scope and Limitations
Author(s): Cornelia Hojnik, Anne Müller, Tobias-Elias Gloe, Thisbe K. Lindhorst,* Tanja M. Wrodnigg*

Table of Contents

S1. Physical Characterization 3
S2. NMR and Mass spectra 4
S3. H/D exchange of Amadori products.. 51
S4. References 54

S1. Physical Characterization

NMR spectra were recorded on Bruker Ultrashield spectrometers at 300.36 (^{1}H) and 75.53 (^{13}C) MHz, respectively. For a higher resolution NMR spectra were recorded on VARIAN INOVA 500 MHz at 500.619 (^{1}H) and 125.894 (^{13}C) MHz, respectively. Chemical shifts are reported relative to internal tetramethylsilane (δ = 0.00 ppm) or D_2O (δ = 4.79 ppm), DMSO-d_6 (δ = 2.50 ppm) or MeOH-d_4 (4.78, 3.31). Full assignment of the peaks was achieved with the aid of 2D NMR techniques (^{1}H-^{1}H COSY and ^{1}H-^{13}C HSQC). MALDI-TOF Mass Spectrometry was performed on a Micromass TofSpec 2E Time-of-Flight Mass Spectrometer.

S2. NMR and Mass spectra

^{1}H and ^{13}C NMR spectra (**4**)

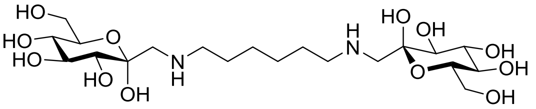

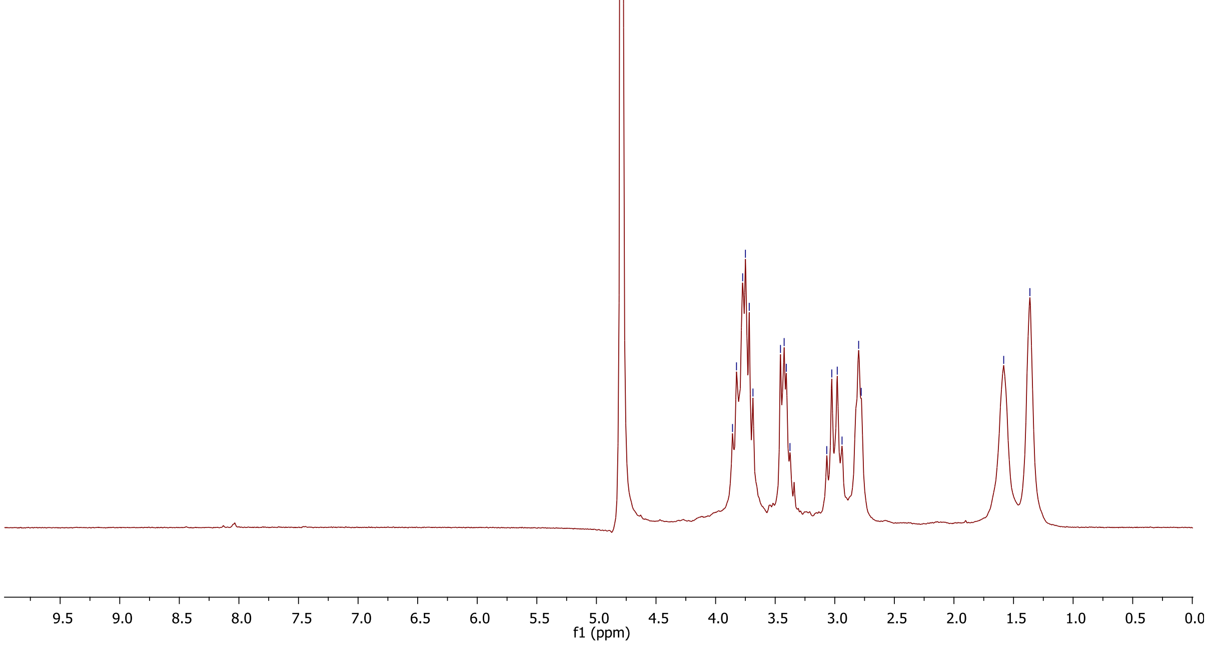

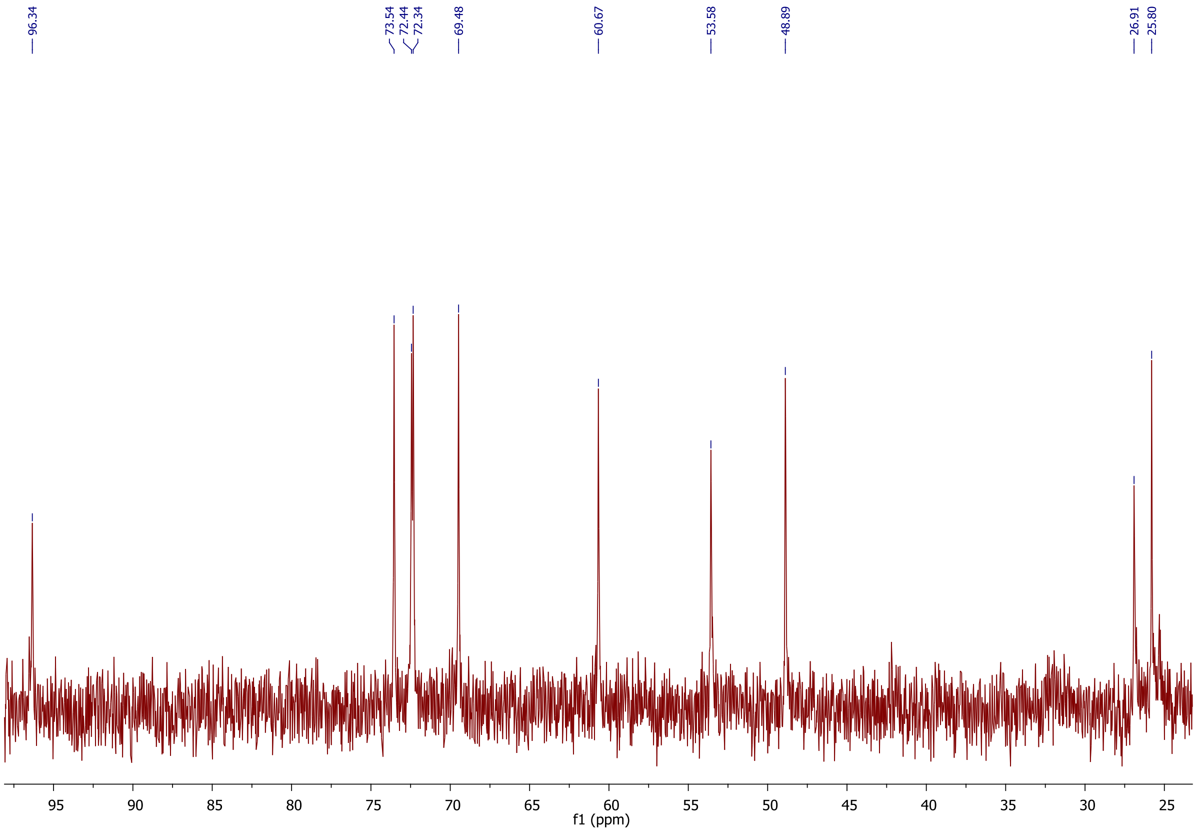

HRMS (MALDI): **4**

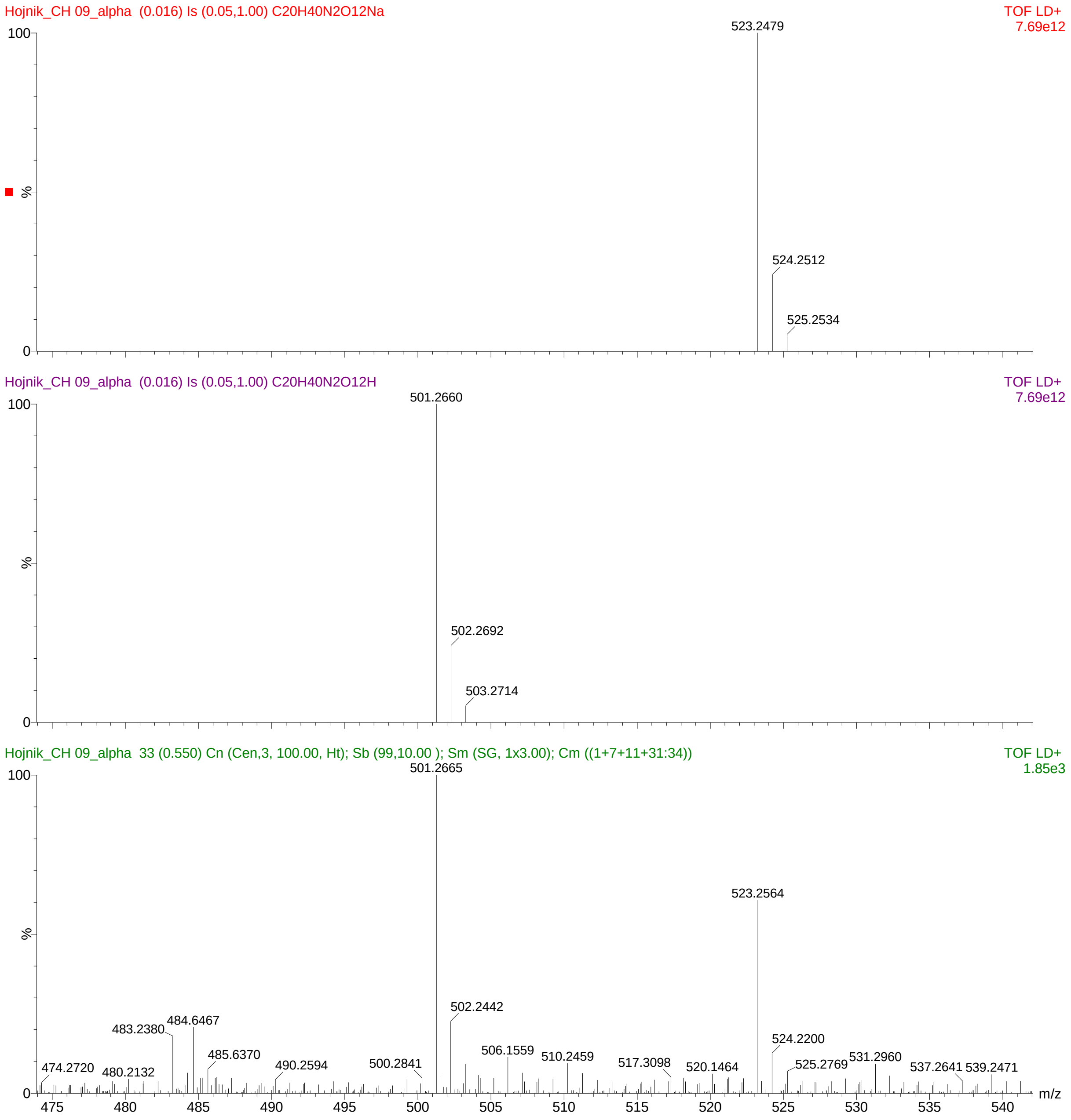
Hojnik_CH 09_alpha (0.016) Is (0.05,1.00) C20H40N2O12Na
TOF LD+
7.69e12
523.2479
524.2512
525.2534
Hojnik_CH 09_alpha (0.016) Is (0.05,1.00) C20H40N2O12H
TOF LD+
7.69e12
501.2660
502.2692
503.2714
Hojnik_CH 09_alpha 33 (0.550) Cn (Cen,3, 100.00, Ht); Sb (99,10.00); Sm (SG, 1x3.00); Cm ((1+7+11+31:34))
TOF LD+
1.85e3
501.2665
523.2564
502.2442
483.2380
484.6467
485.6370
474.2720
480.2132
490.2594
500.2841
506.1559
510.2459
517.3098
520.1464
524.2200
525.2769
531.2960
537.2641
539.2471
100
%
0
475
480
485
490
495
500
505
510
515
520
525
530
535
540
m/z

^{1}H and ^{13}C NMR spectra (**5**)

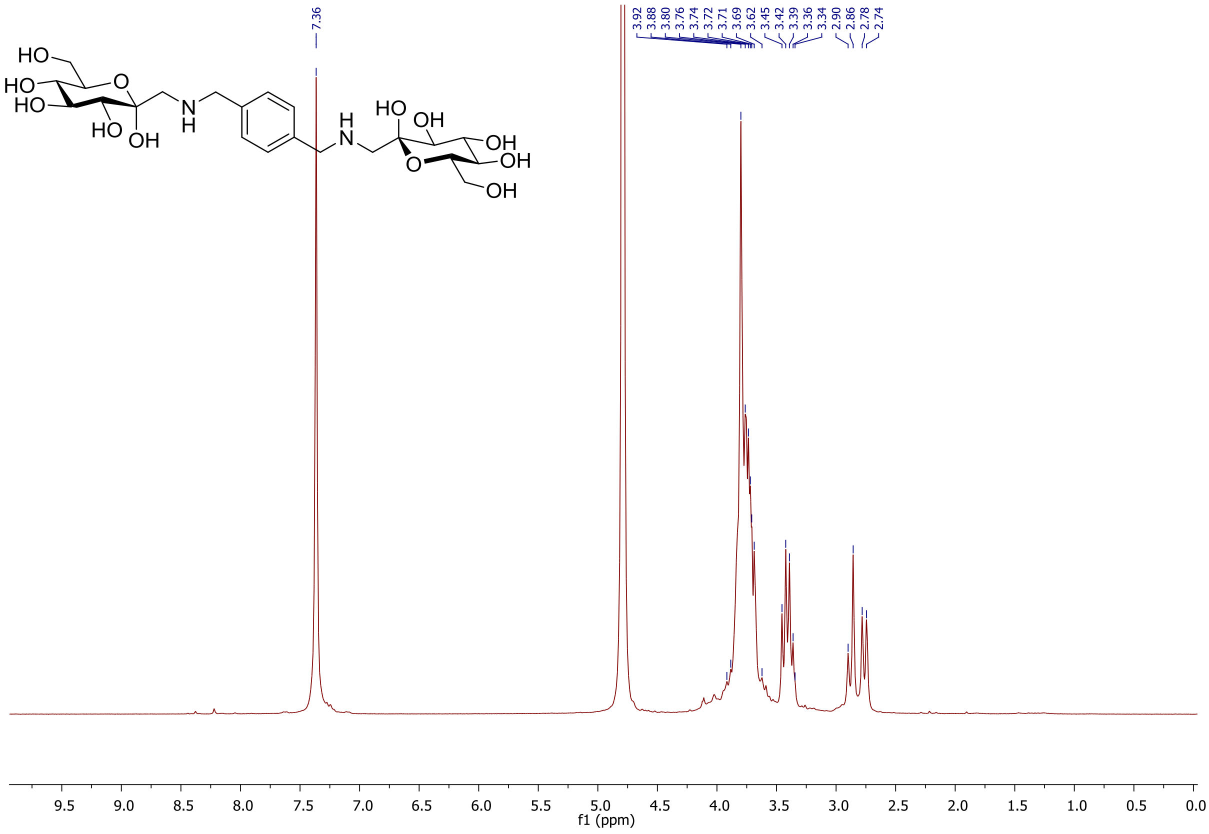

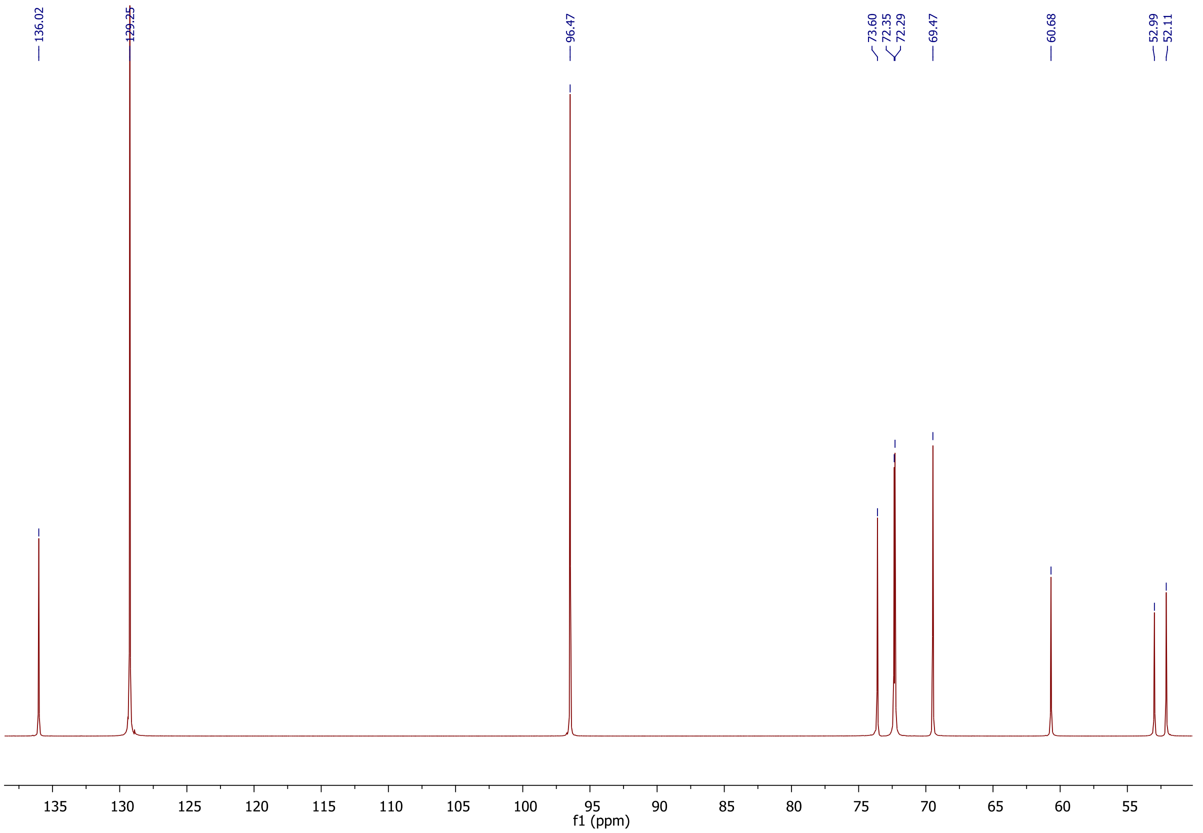

HRMS (MALDI): **5**

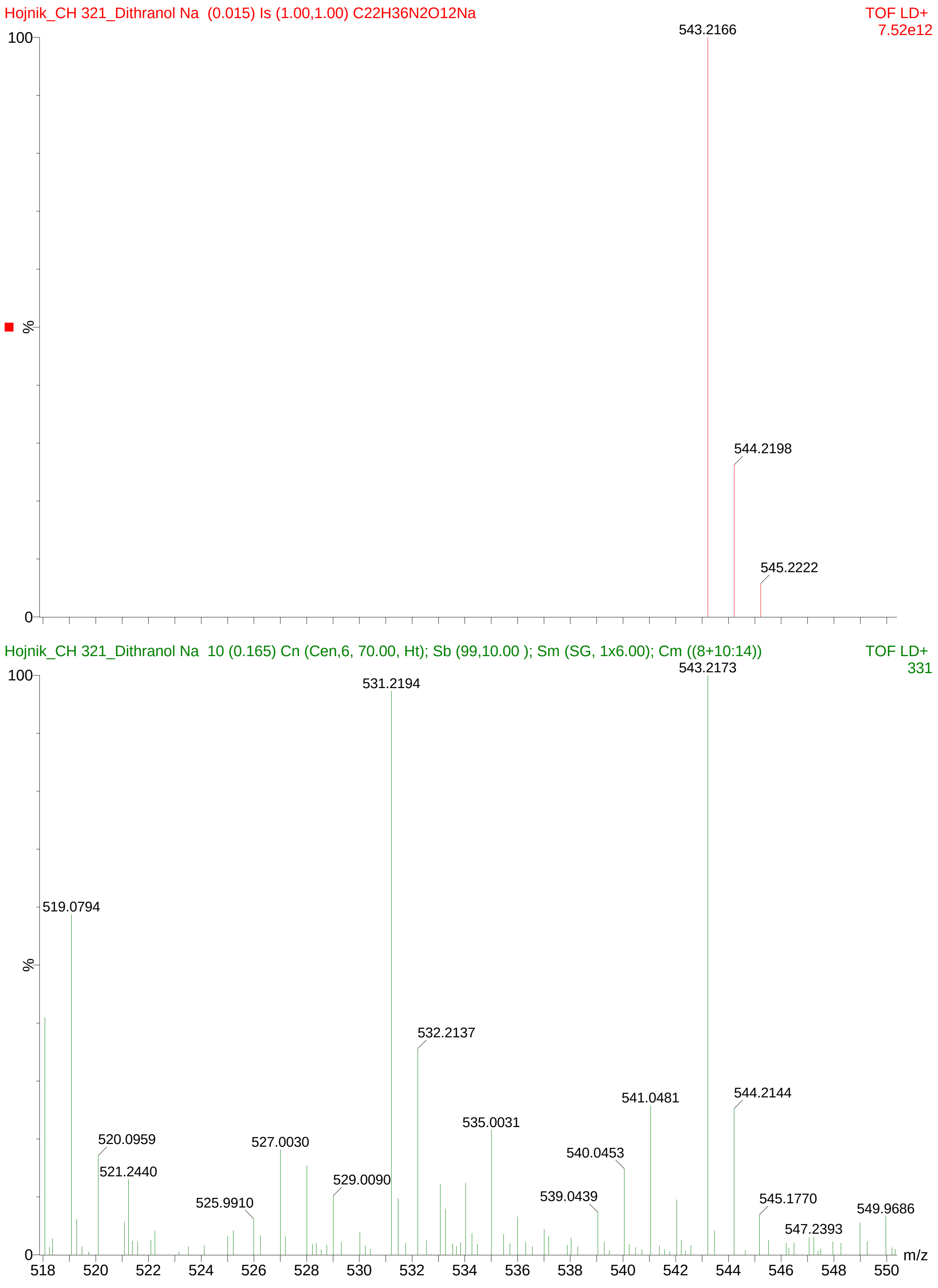
Hojnik_CH 321_Dithranol Na (0.015) Is (1.00,1.00) C22H36N2O12Na
TOF LD+
7.52e12
543.2166
544.2198
545.2222
Hojnik_CH 321_Dithranol Na 10 (0.165) Cn (Cen,6, 70.00, Ht); Sb (99,10.00); Sm (SG, 1x6.00); Cm ((8+10:14))
TOF LD+
331
543.2173
531.2194
519.0794
532.2137
541.0481
544.2144
535.0031
520.0959
527.0030
540.0453
521.2440
529.0090
539.0439
525.9910
545.1770
549.9686
547.2393
m/z

^{1}H and ^{13}C NMR spectra (**7**)

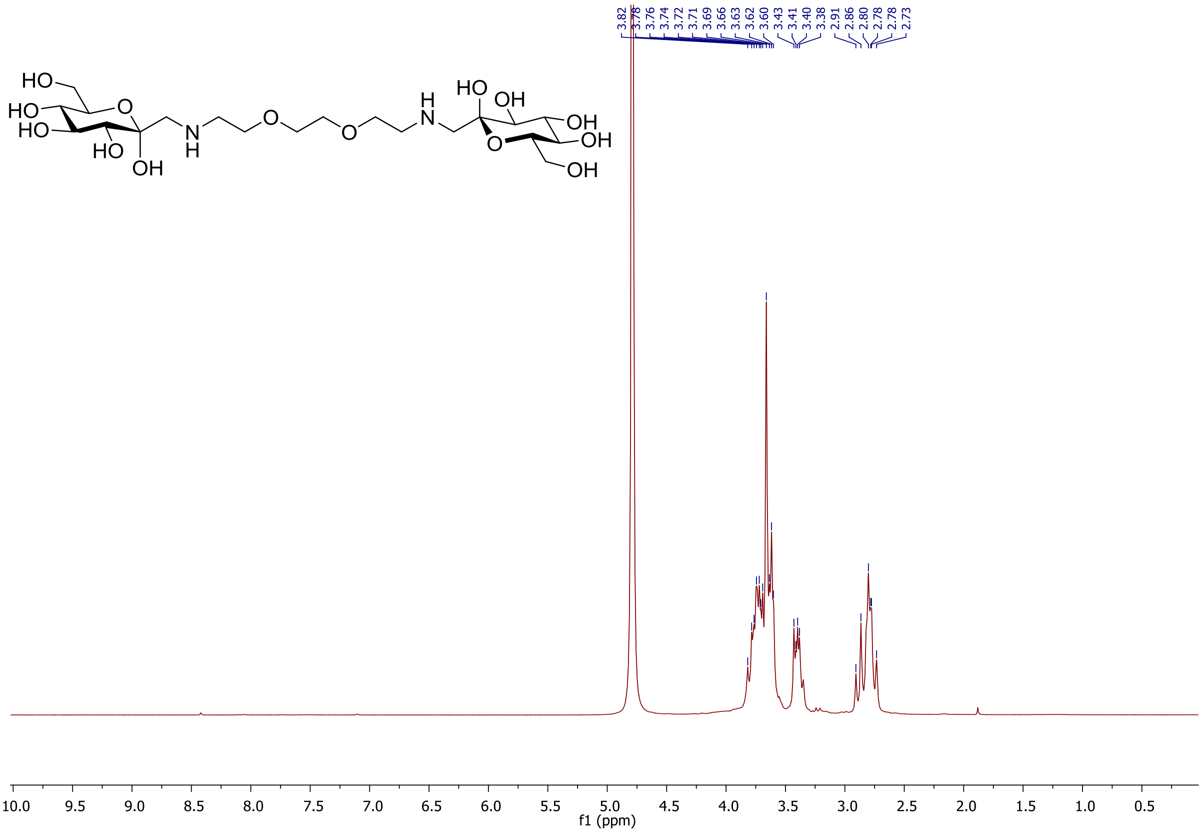

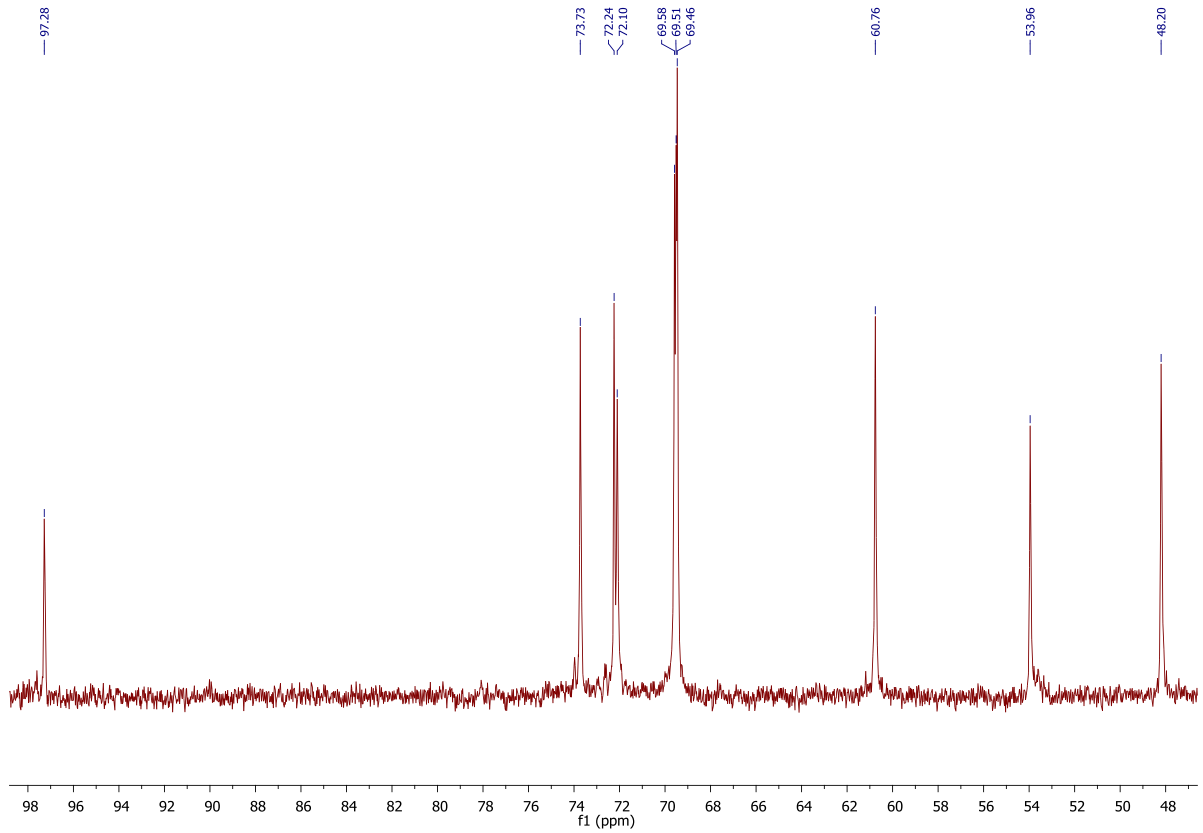

HRMS (MALDI): **7**

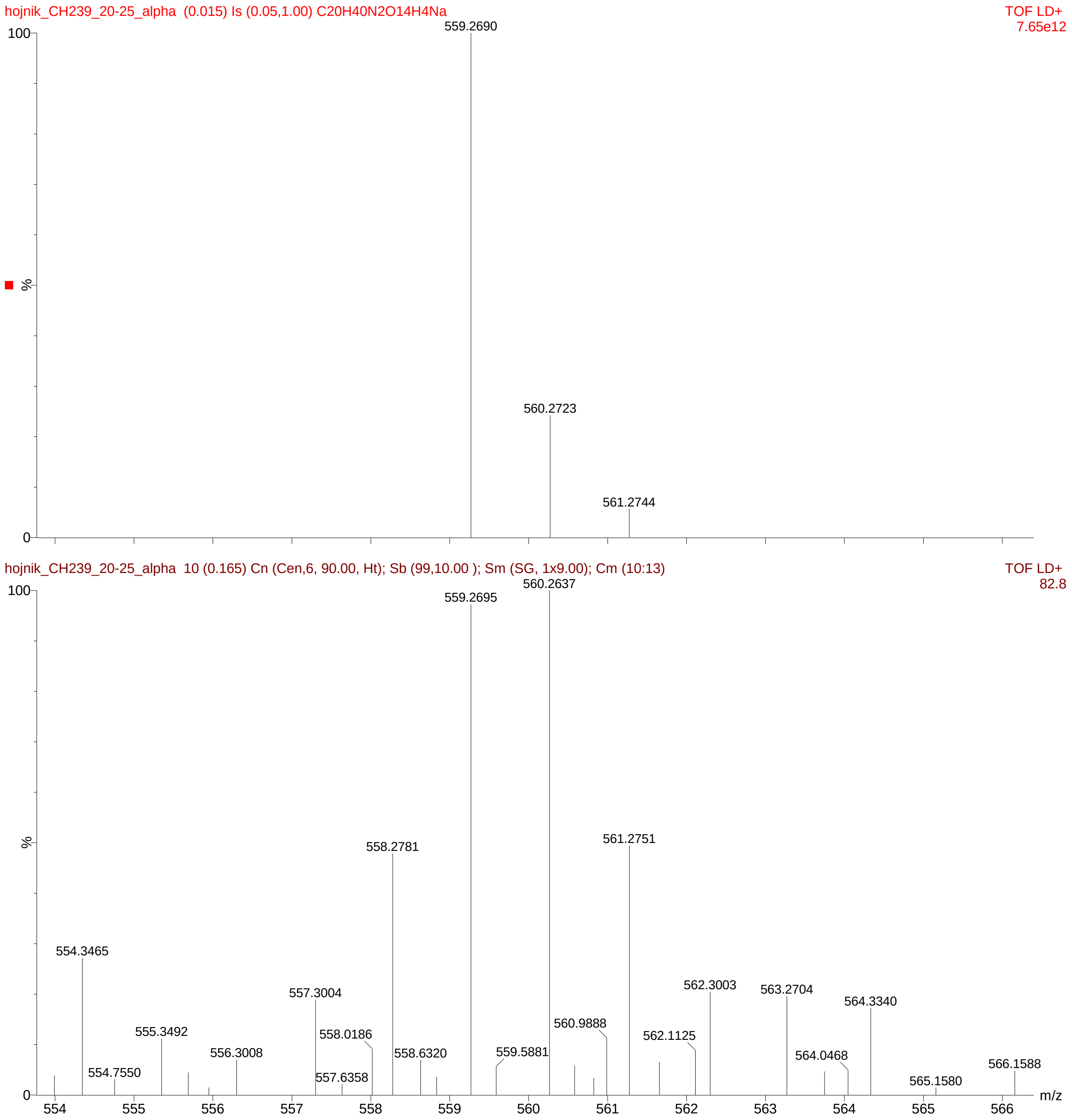
hojnik_CH239_20-25_alpha (0.015) Is (0.05,1.00) C20H40N2O14H4Na
TOF LD+
7.65e12
559.2690
560.2723
561.2744
%
hojnik_CH239_20-25_alpha 10 (0.165) Cn (Cen,6, 90.00, Ht); Sb (99,10.00); Sm (SG, 1x9.00); Cm (10:13)
TOF LD+
82.8
559.2695
560.2637
558.2781
561.2751
554.3465
557.3004
562.3003
563.2704
564.3340
555.3492
558.0186
560.9888
562.1125
556.3008
558.6320
559.5881
564.0468
554.7550
557.6358
566.1588
565.1580
m/z

^{1}H and ^{13}C NMR spectra (**8**)

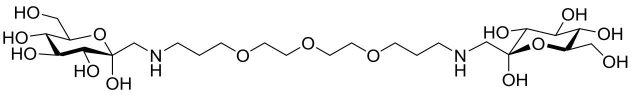

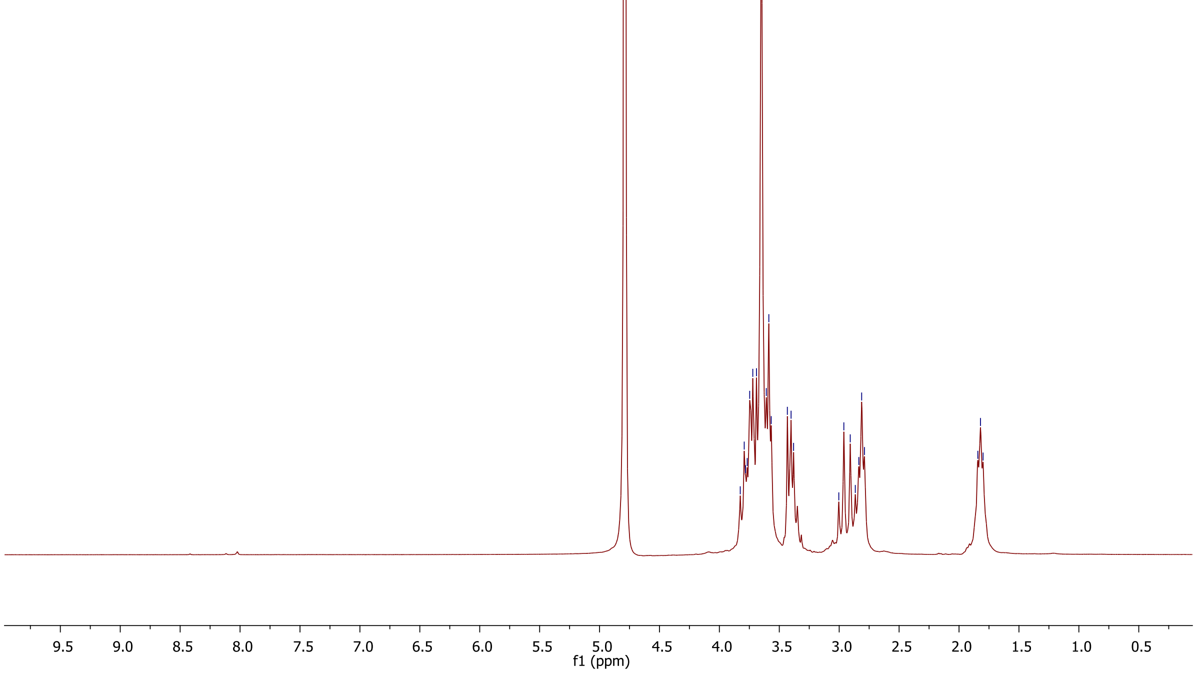

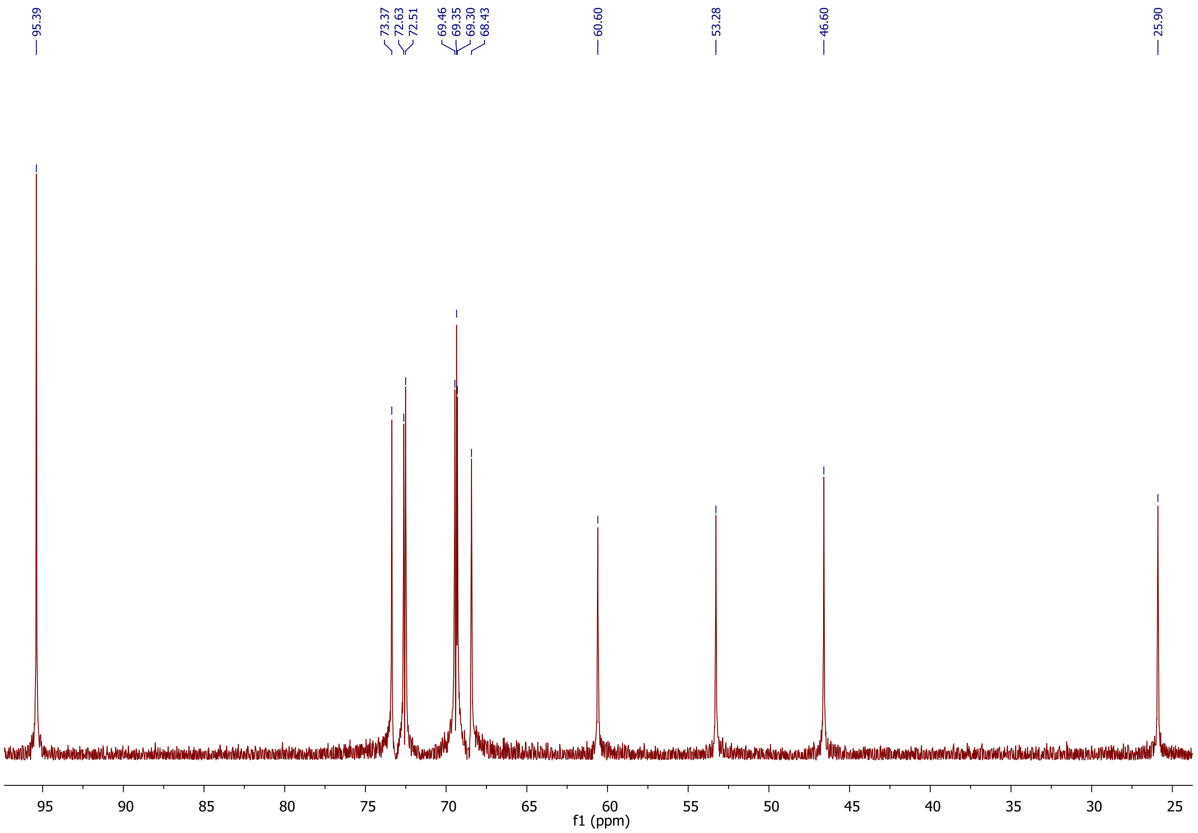

HRMS (MALDI): **8**

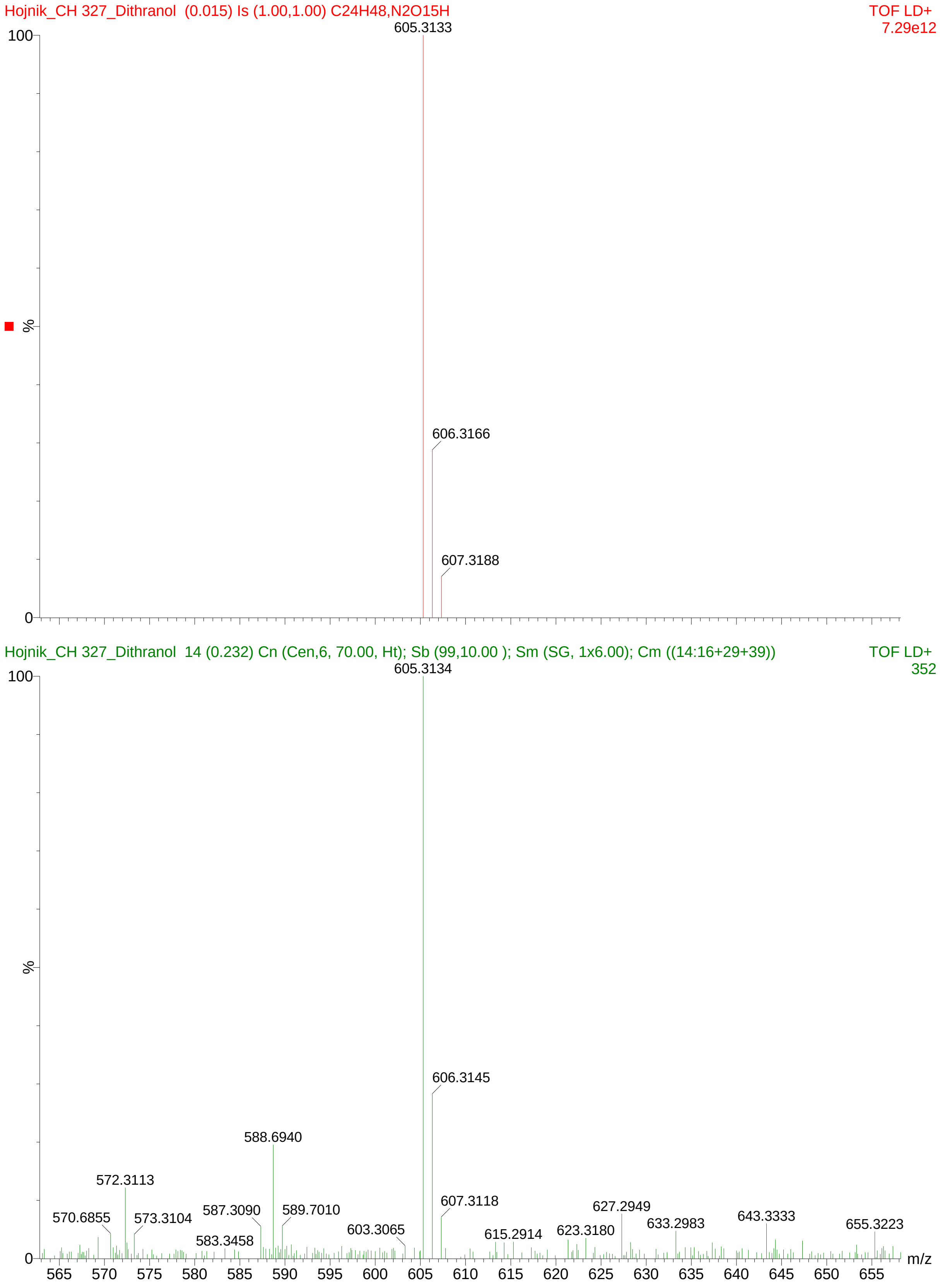
Hojnik_CH 327_Dithranol (0.015) Is (1.00,1.00) C24H48,N2O15H
TOF LD+
7.29e12
605.3133
606.3166
607.3188
100
%
0
Hojnik_CH 327_Dithranol 14 (0.232) Cn (Cen,6, 70.00, Ht); Sb (99,10.00); Sm (SG, 1x6.00); Cm ((14:16+29+39))
TOF LD+
352
605.3134
606.3145
588.6940
572.3113
570.6855
573.3104
587.3090
589.7010
583.3458
603.3065
607.3118
615.2914
623.3180
627.2949
633.2983
643.3333
655.3223
100
%
0
565 570 575 580 585 590 595 600 605 610 615 620 625 630 635 640 645 650 655
m/z

^{1}H and ^{13}C NMR spectra (**10**)

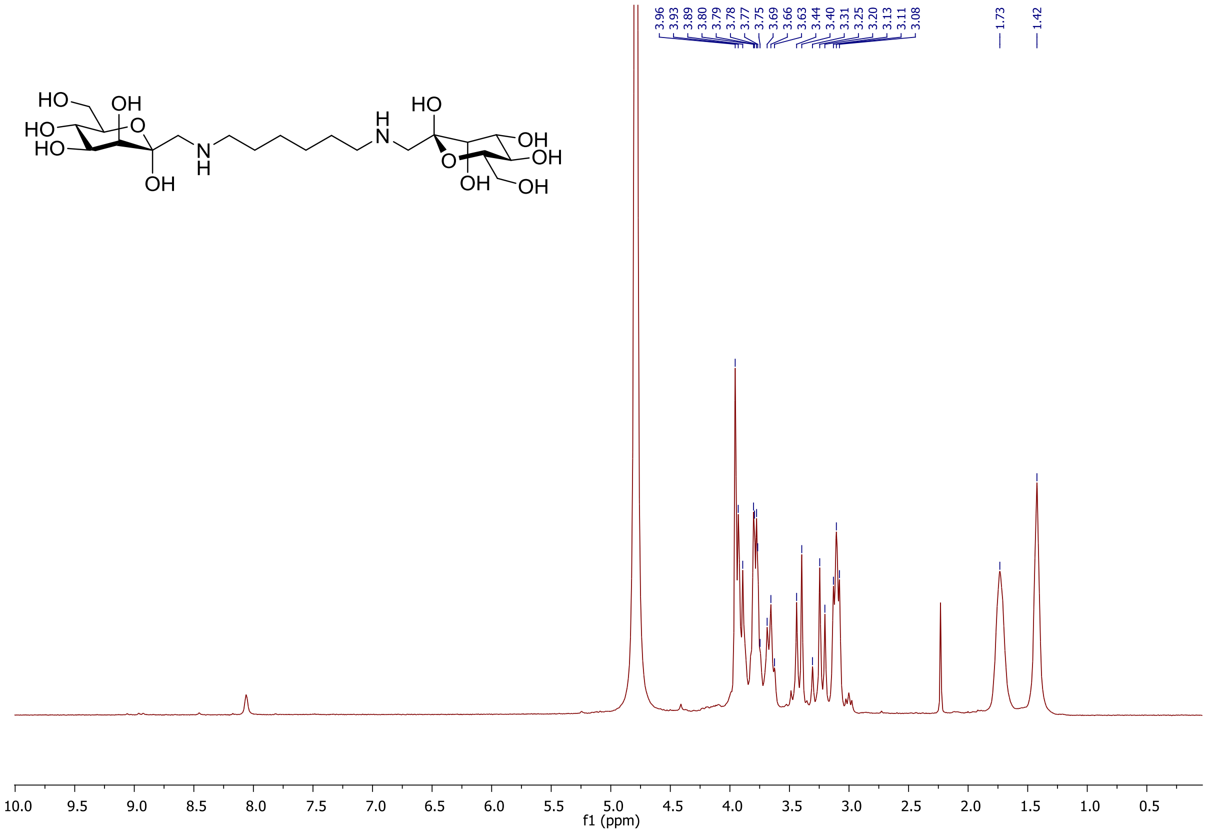

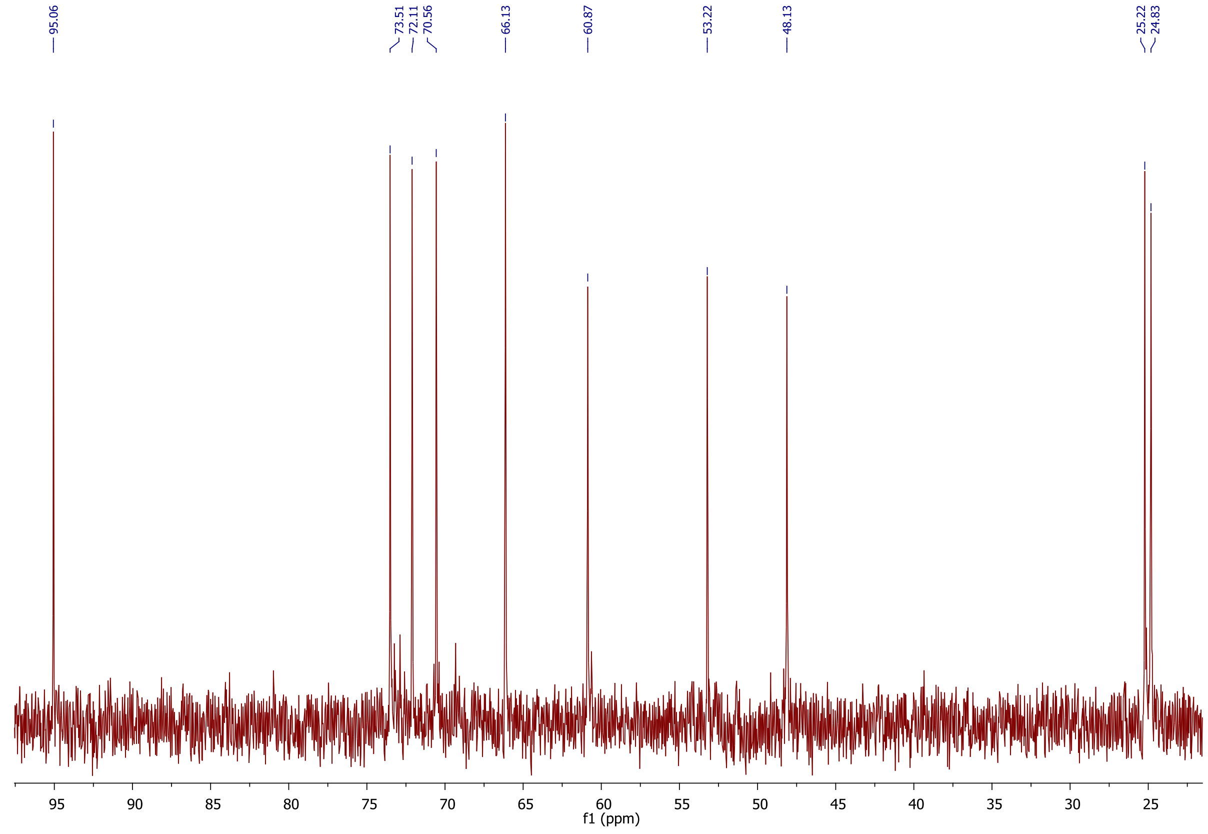

HRMS (MALDI): **10**

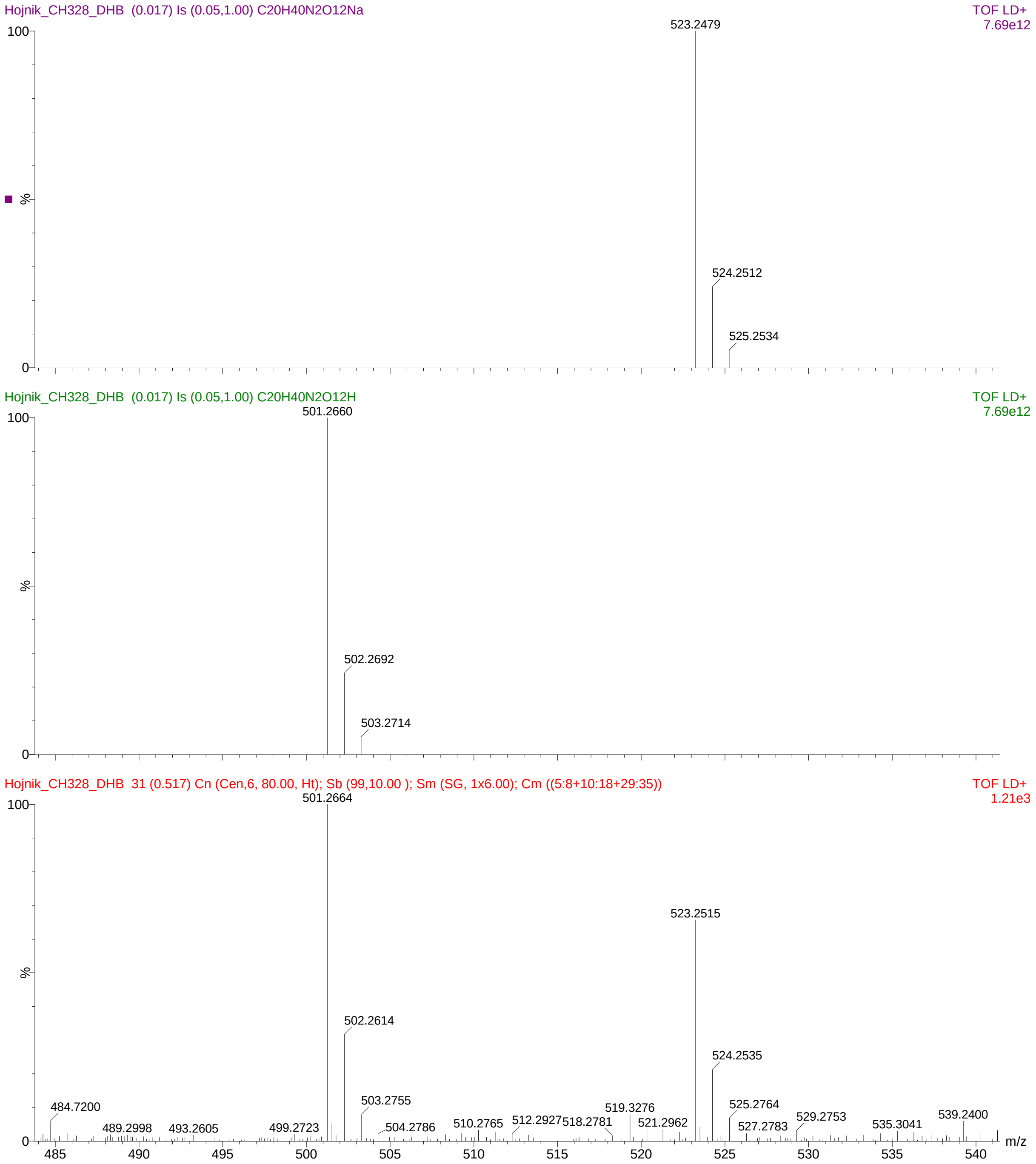

^{1}H and ^{13}C NMR spectra (**11**)

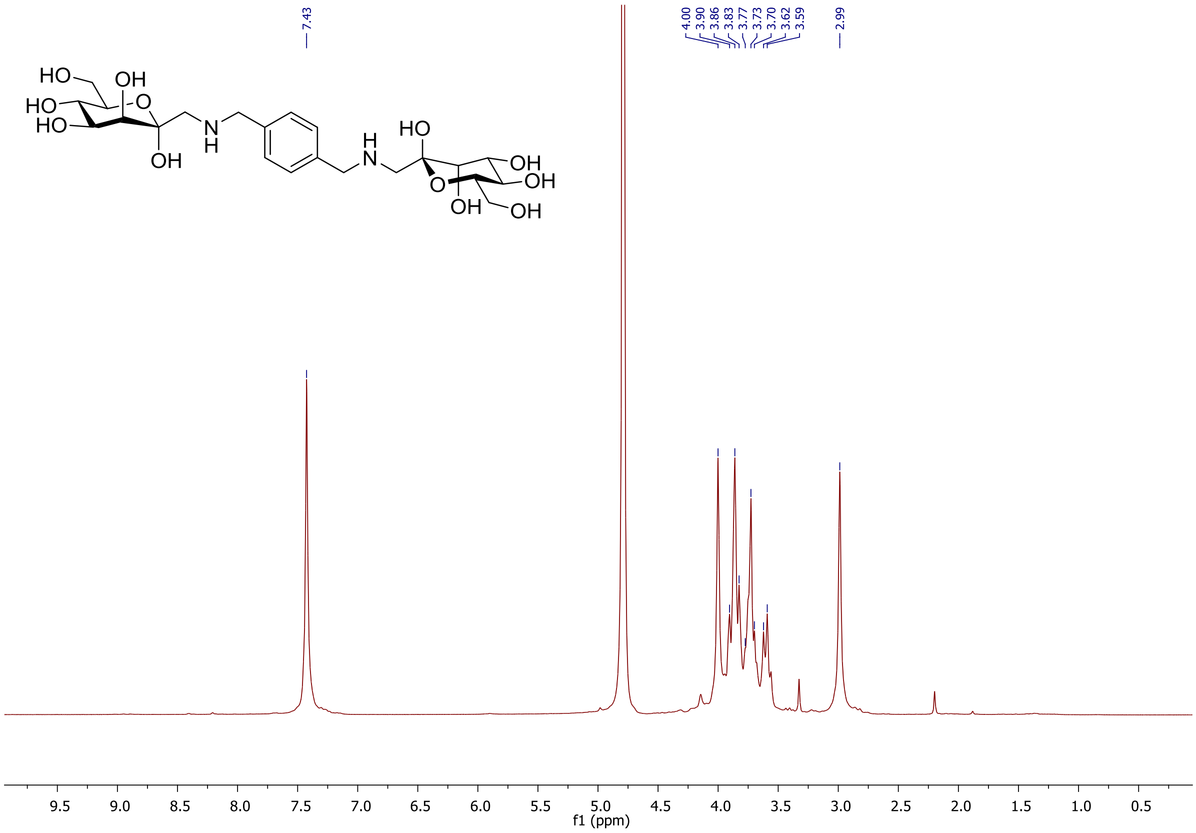

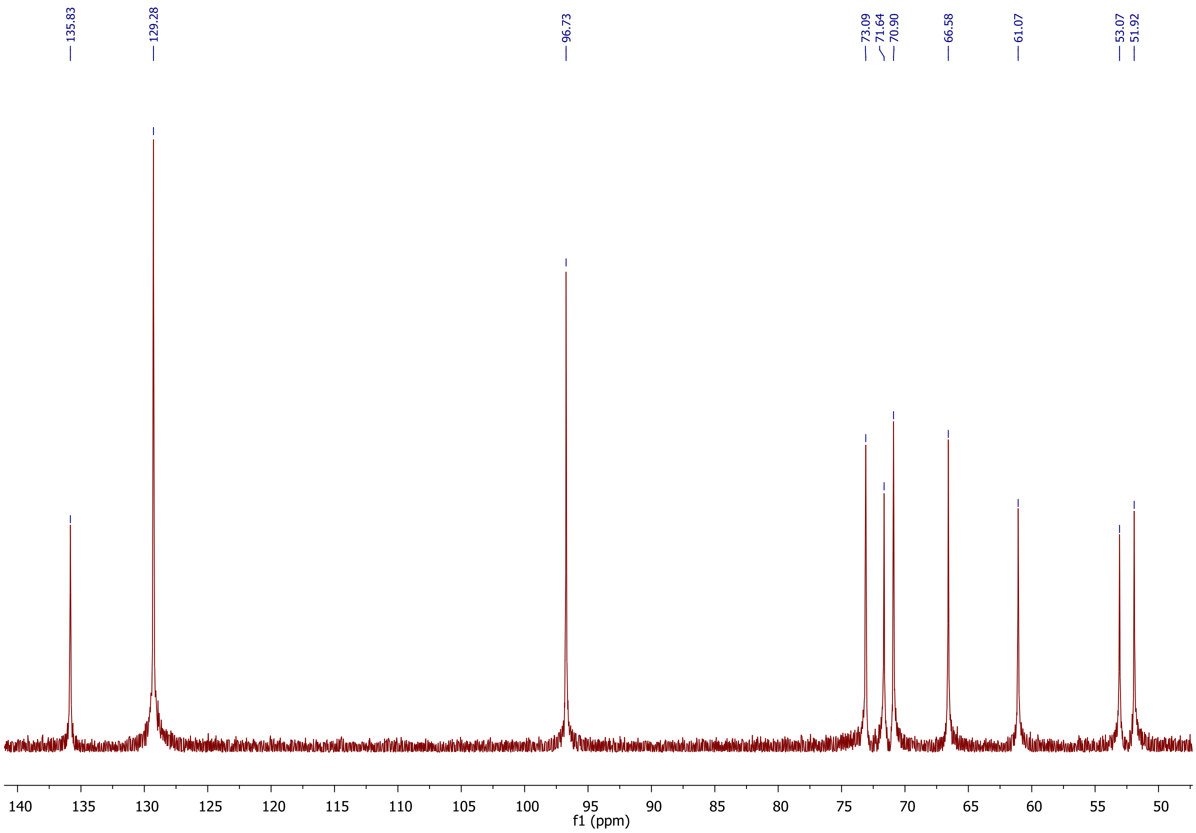

HRMS (MALDI): **11**

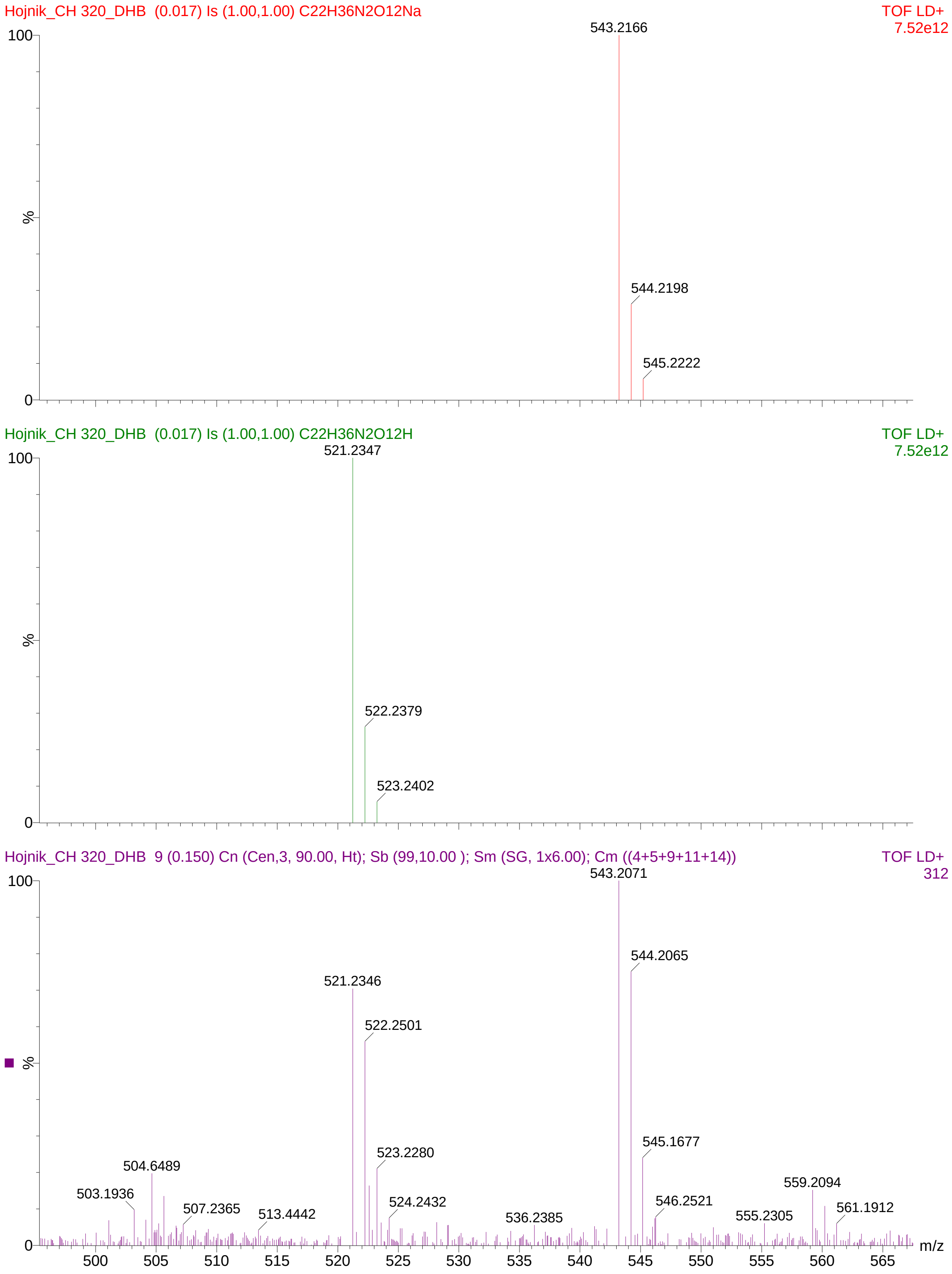

^{1}H and ^{13}C NMR spectra (**13**)

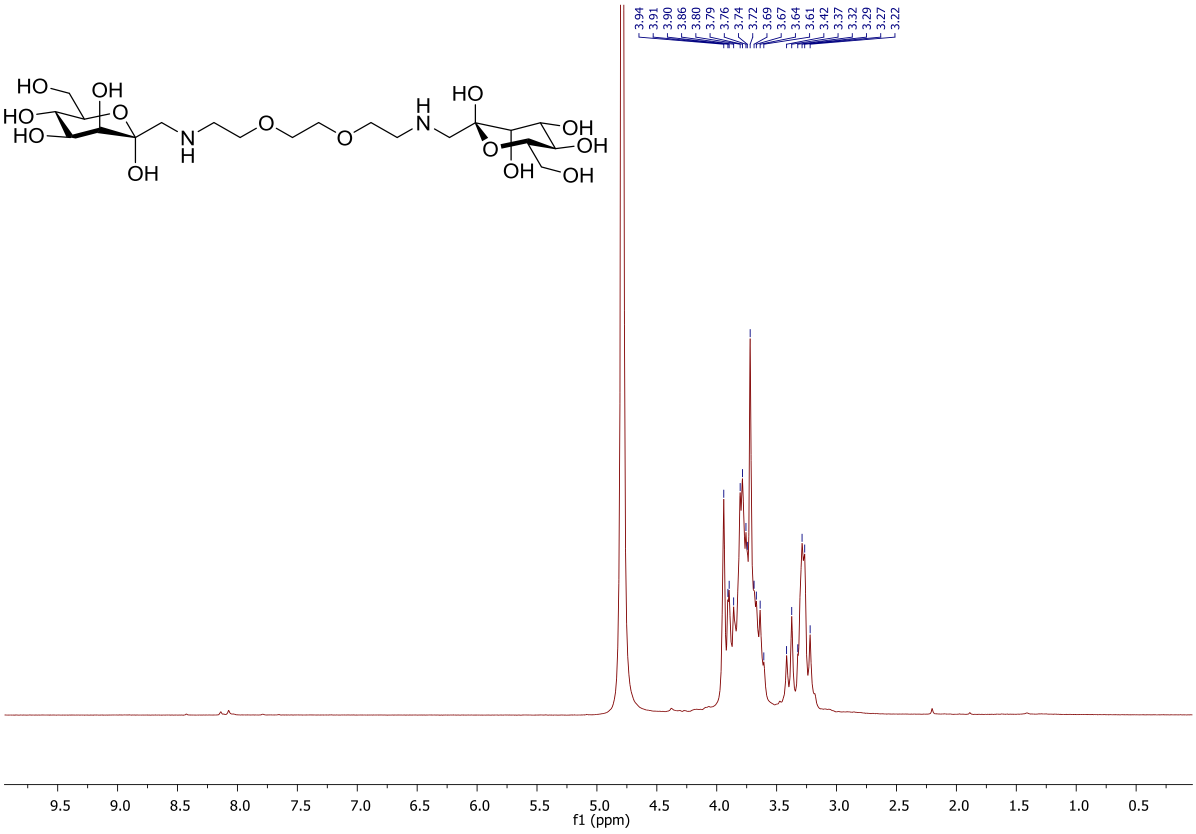

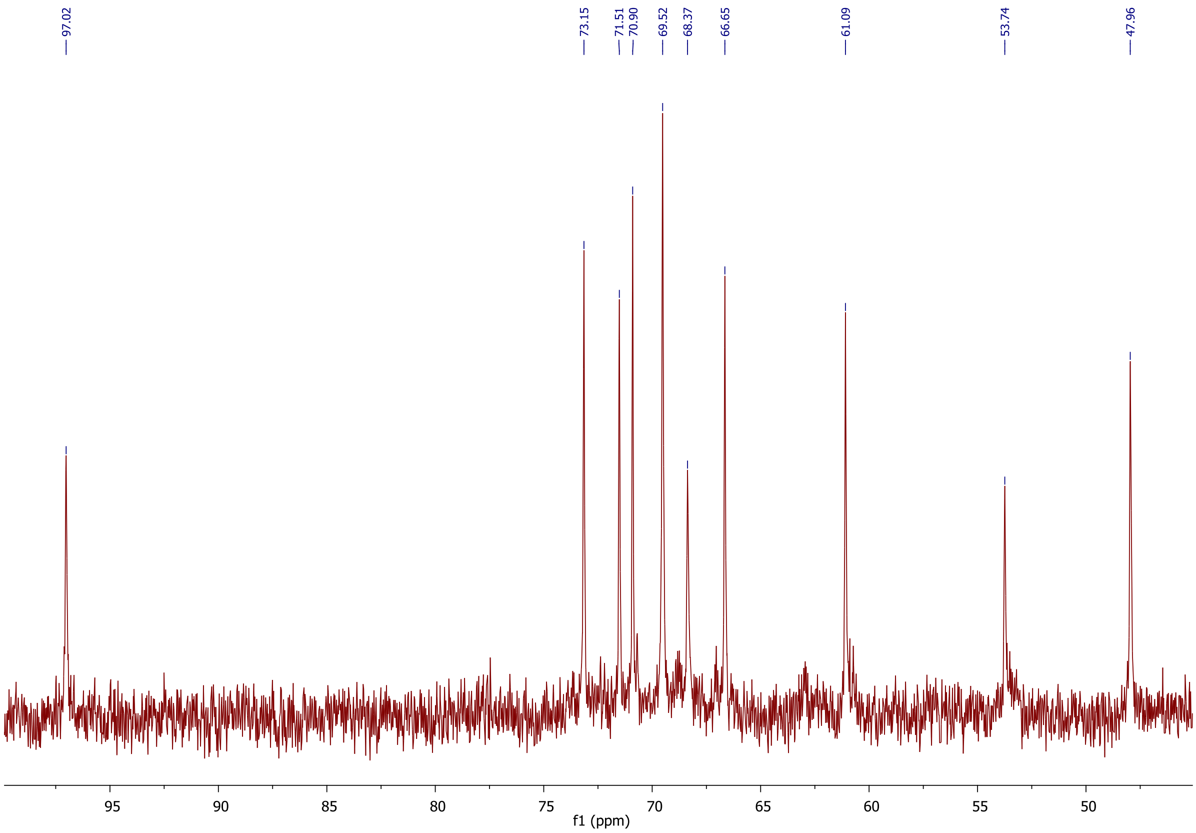

HRMS (MALDI): **13**

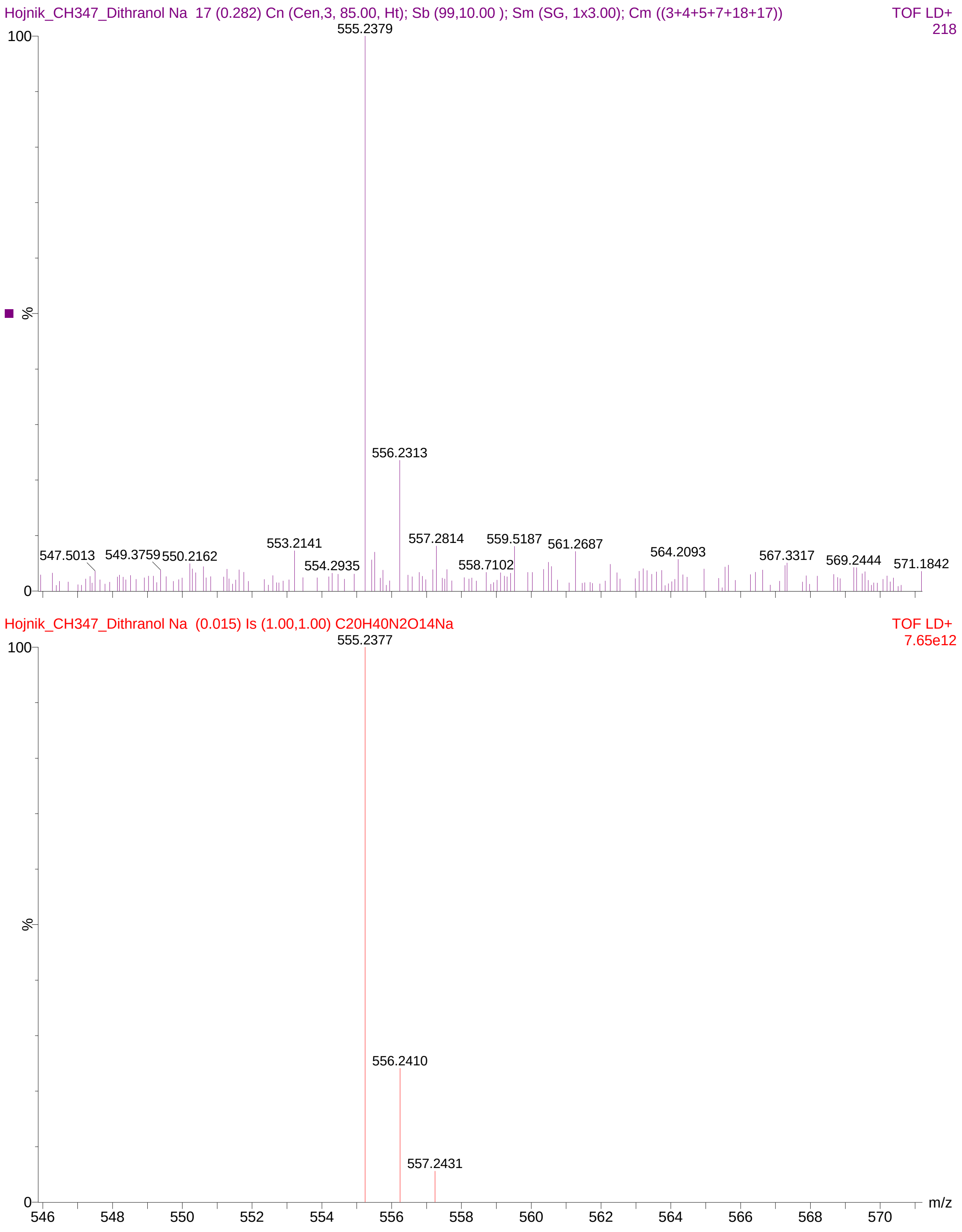

^{1}H and ^{13}C NMR spectra (**14**)

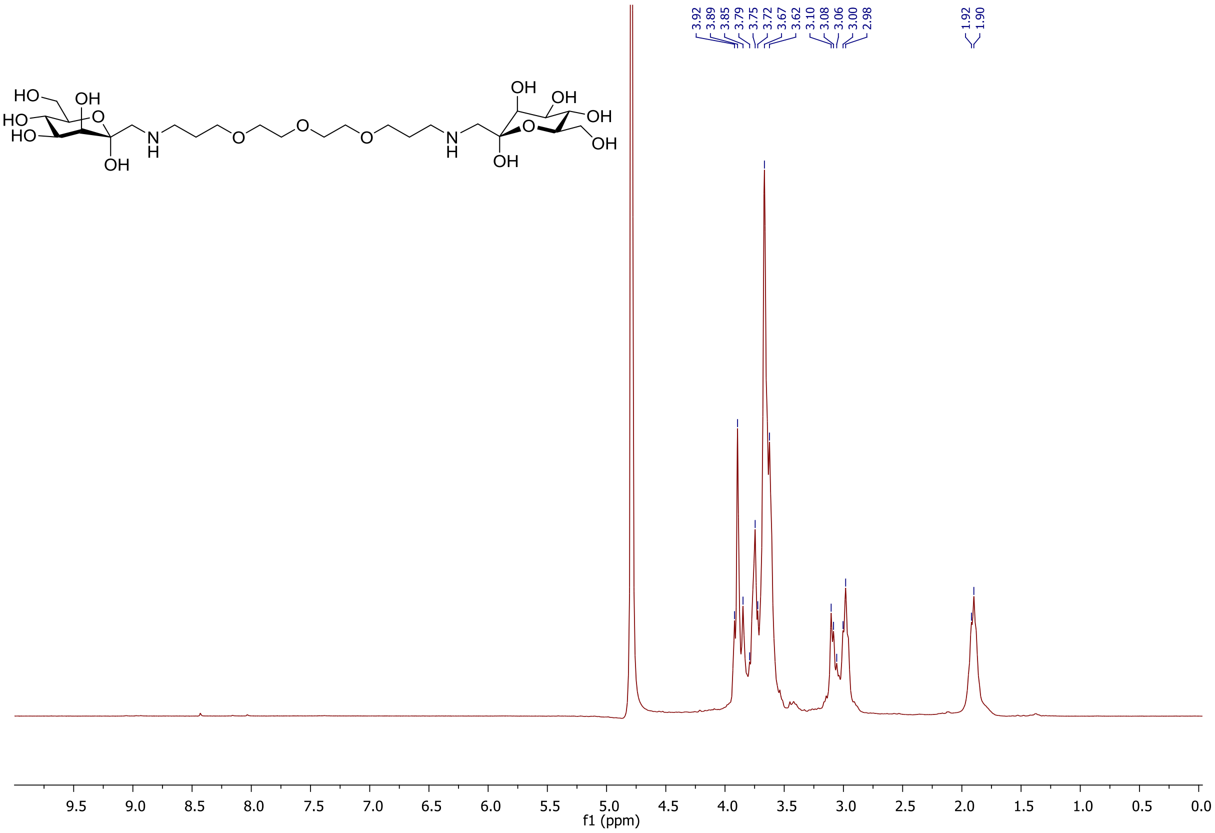

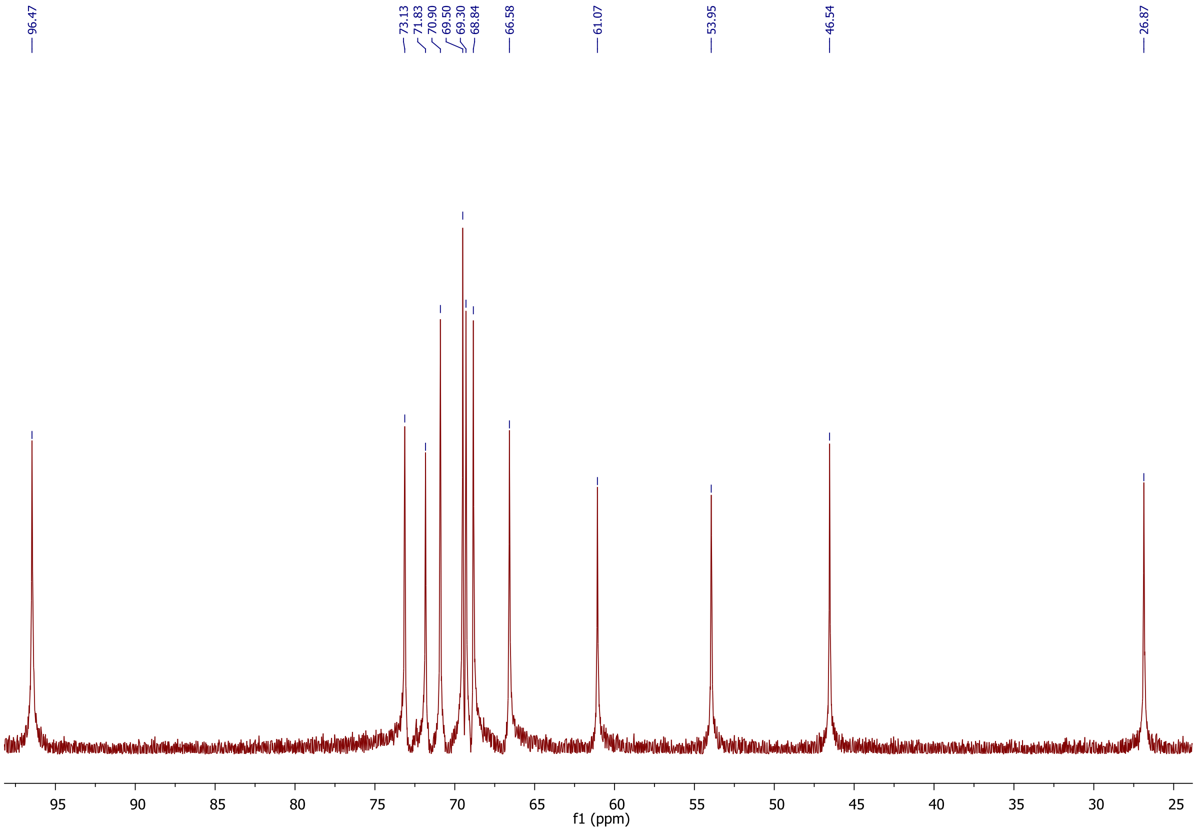

HRMS (MALDI): **14**

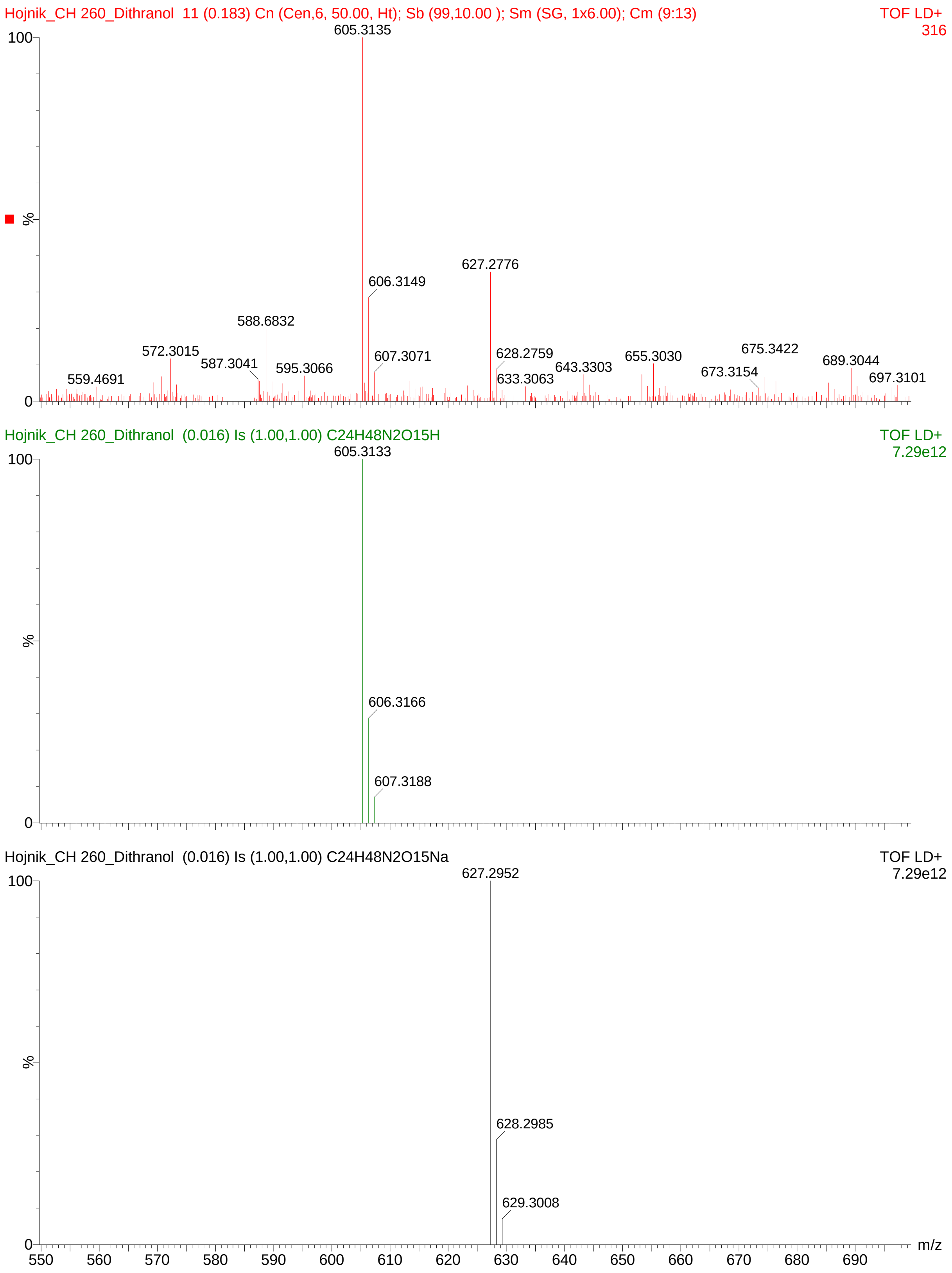

^{1}H and ^{13}C NMR spectra (**16**)

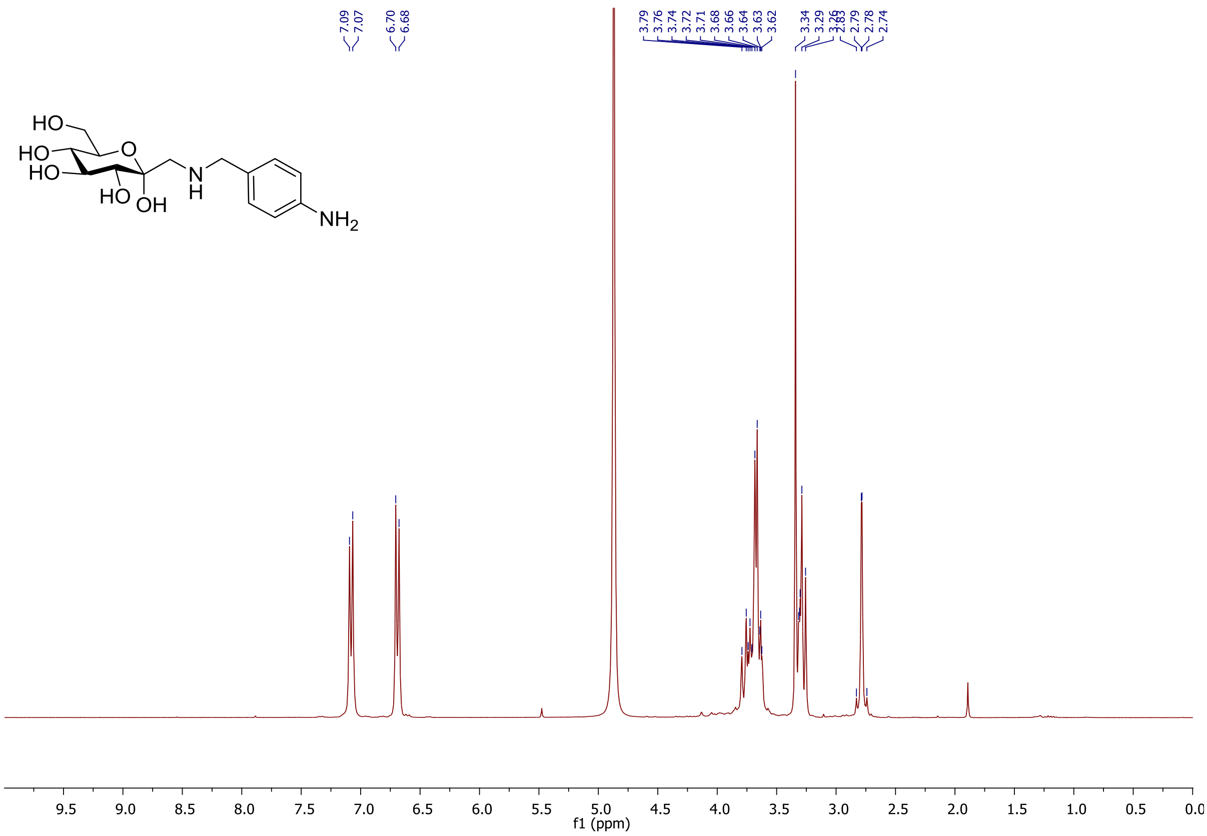

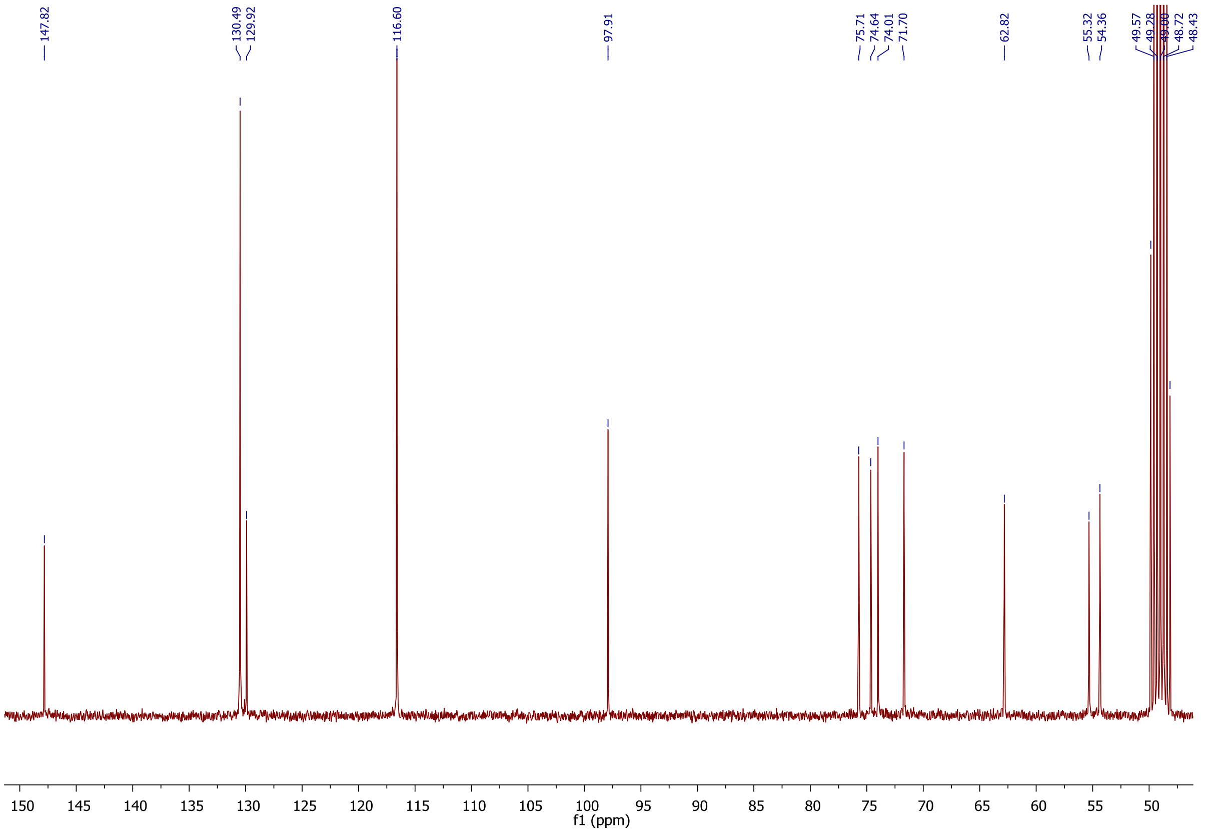

ESI-MS: **16**

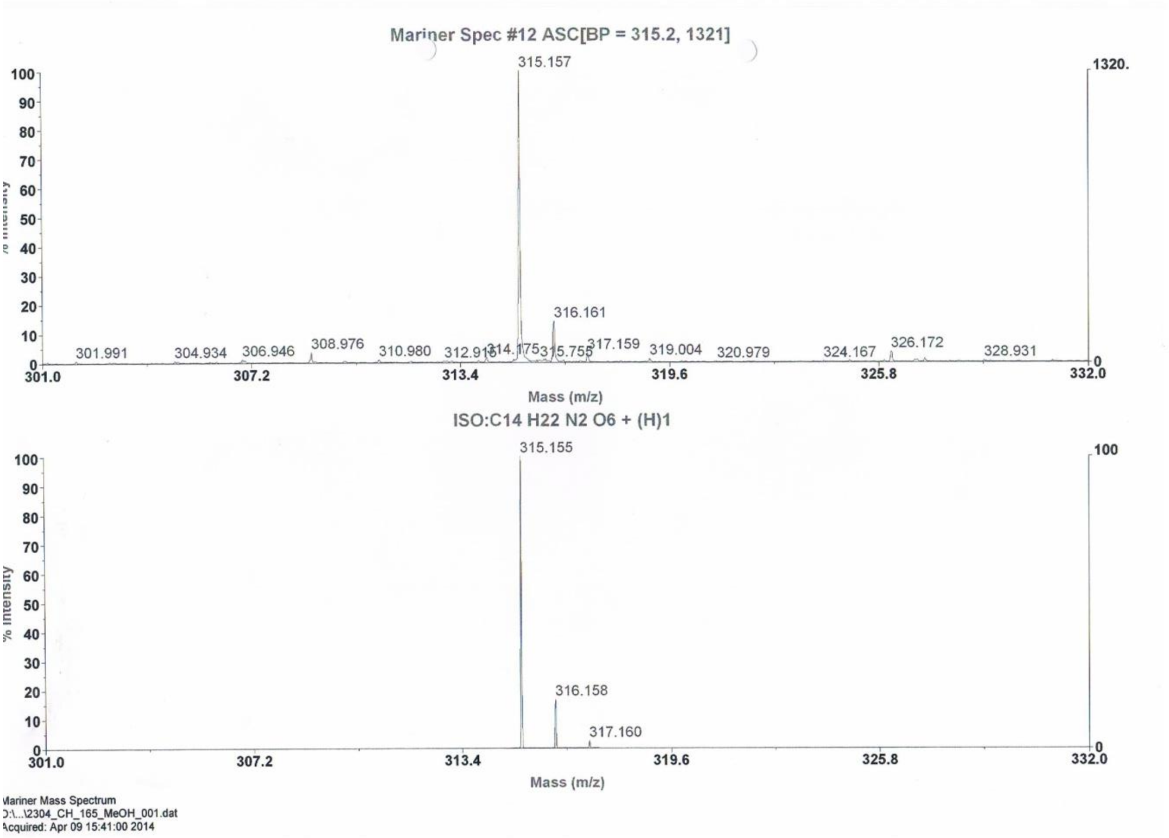
Mariner Spec #12 ASC[BP = 315.2, 1321]
315.157
316.161
301.991
304.934
306.946
308.976
310.980
312.916
314.175
315.755
317.159
319.004
320.979
324.167
326.172
328.931
1320.
Mass (m/z)
ISO:C14 H22 N2 O6 + (H)1
315.155
316.158
317.160
% Intensity
Mass (m/z)
Mariner Mass Spectrum
D:\...\2304_CH_165_MeOH_001.dat
Acquired: Apr 09 15:41:00 2014

^{1}H and ^{13}C NMR spectra (**17**)

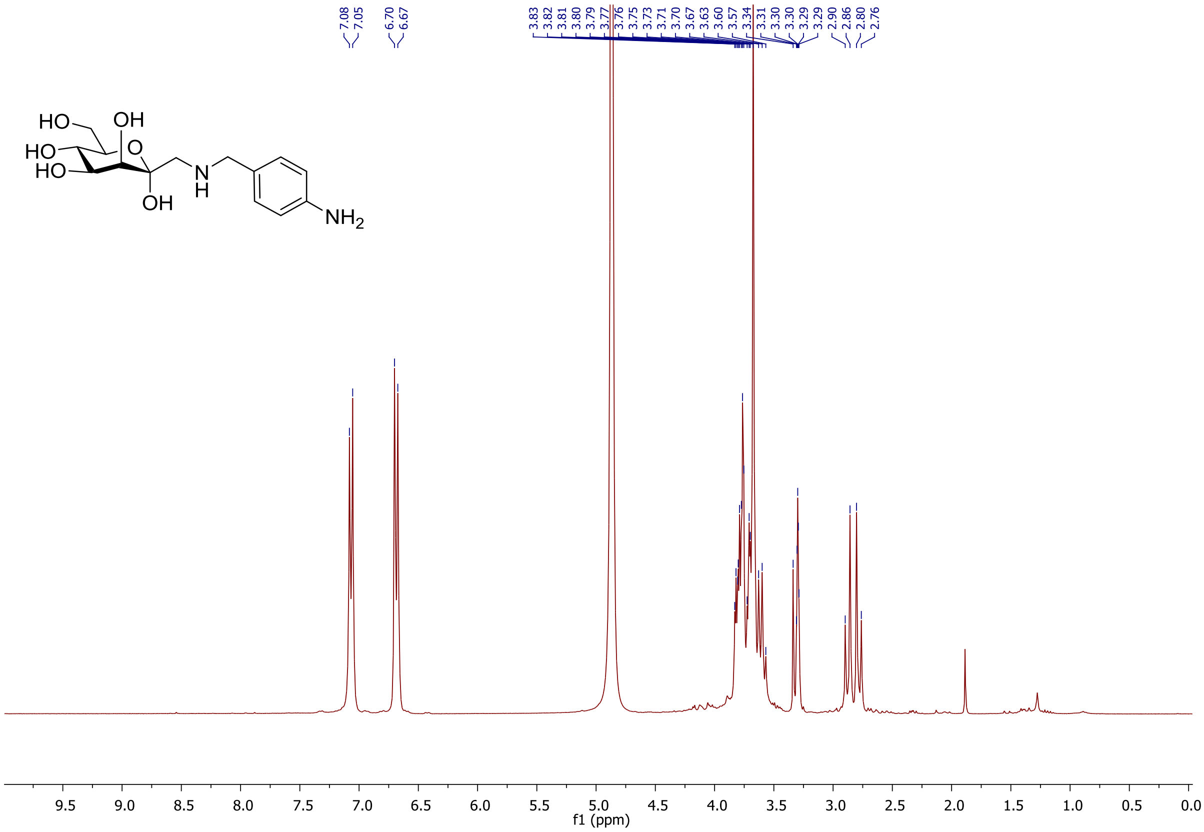

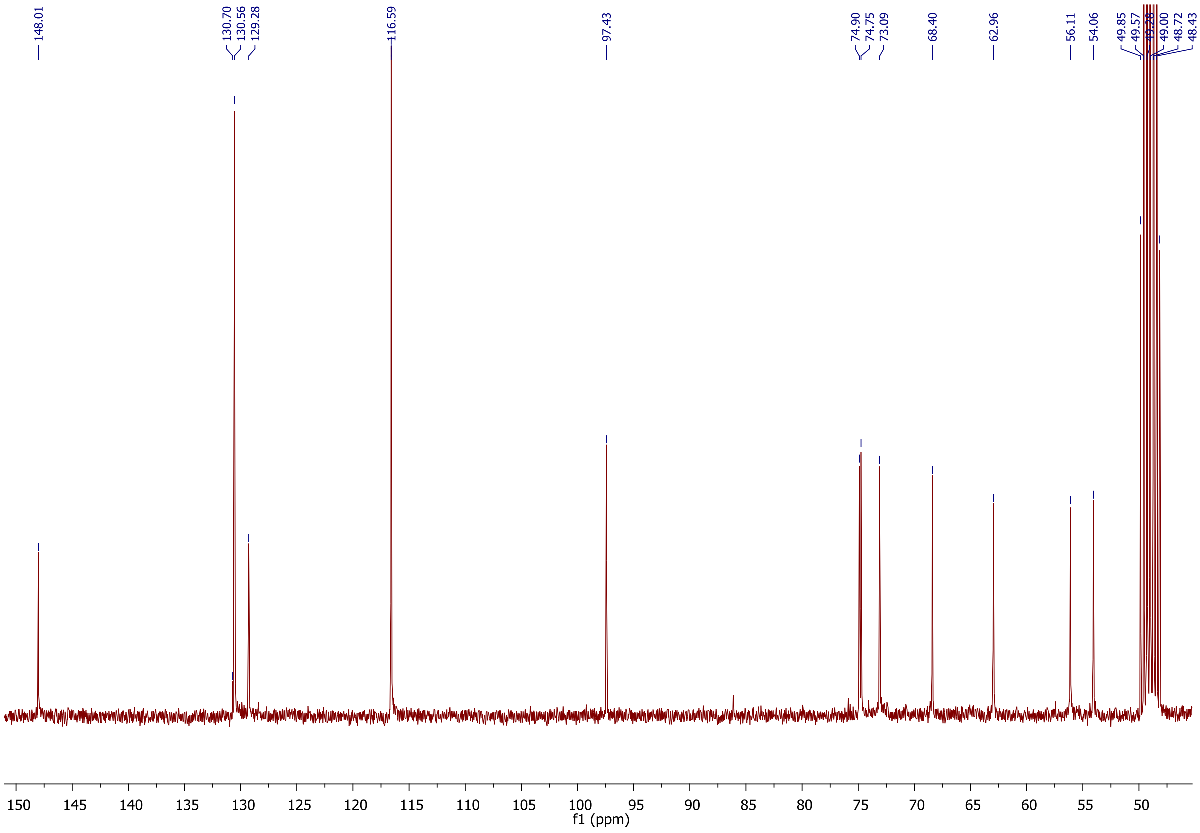

HRMS (MALDI): **17**

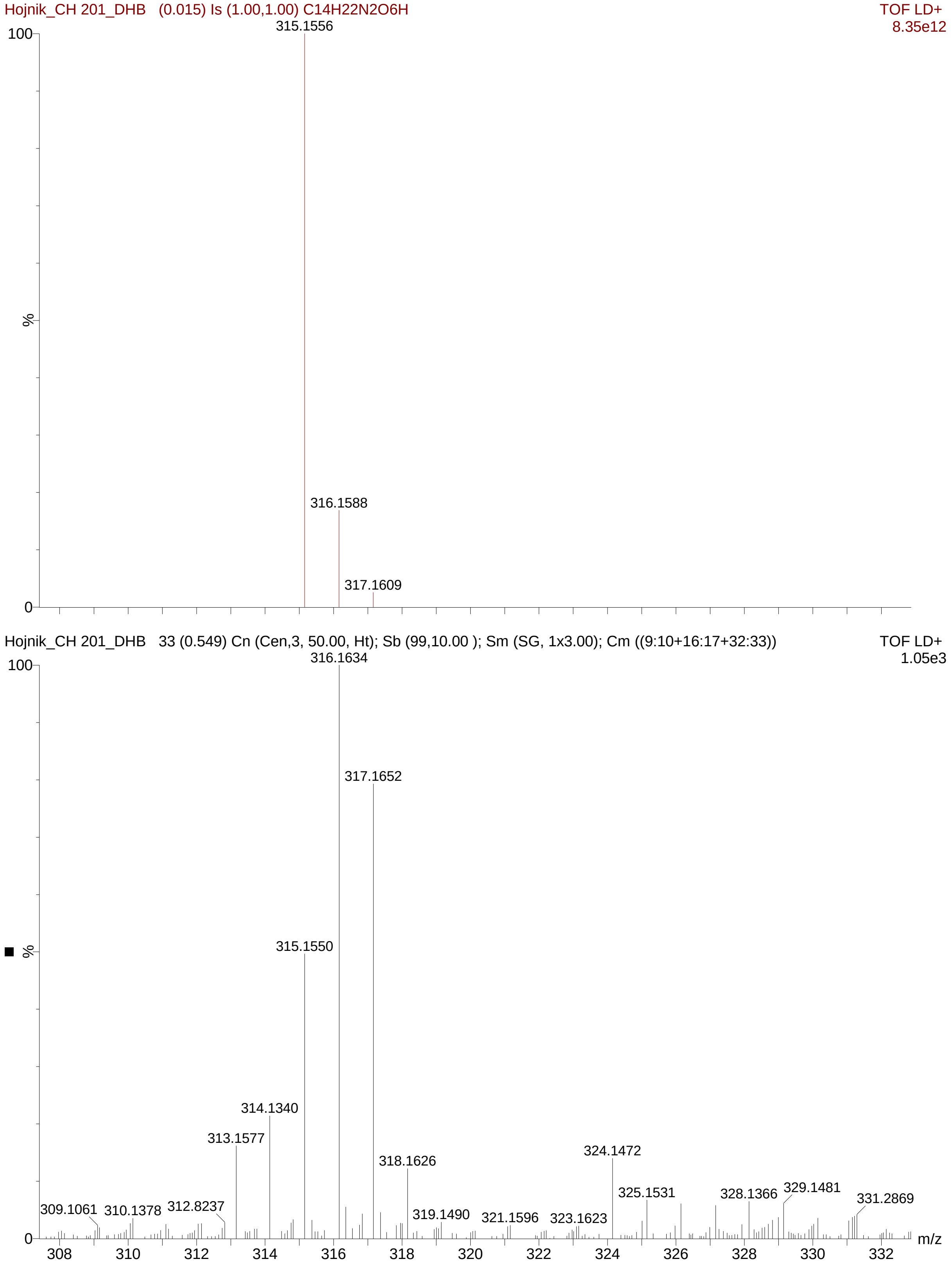

^{1}H and ^{13}C NMR spectra (**19**)

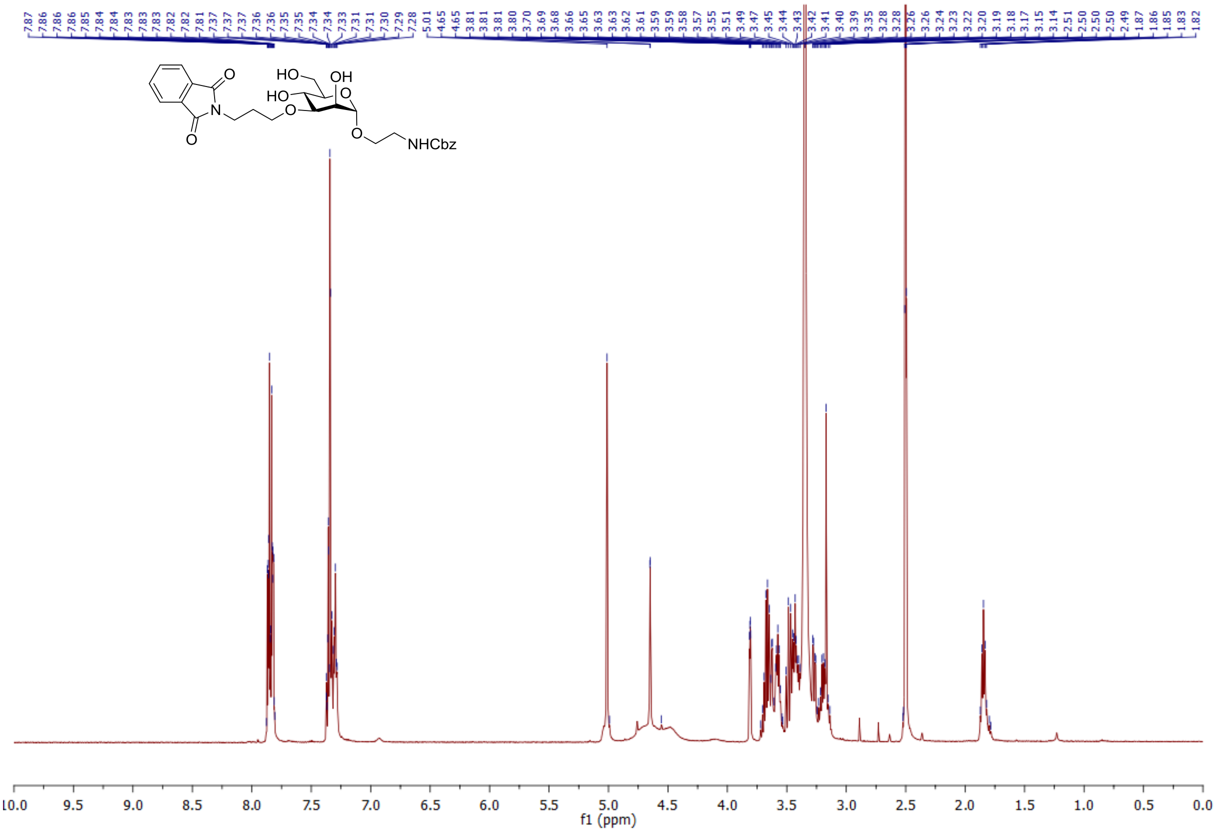

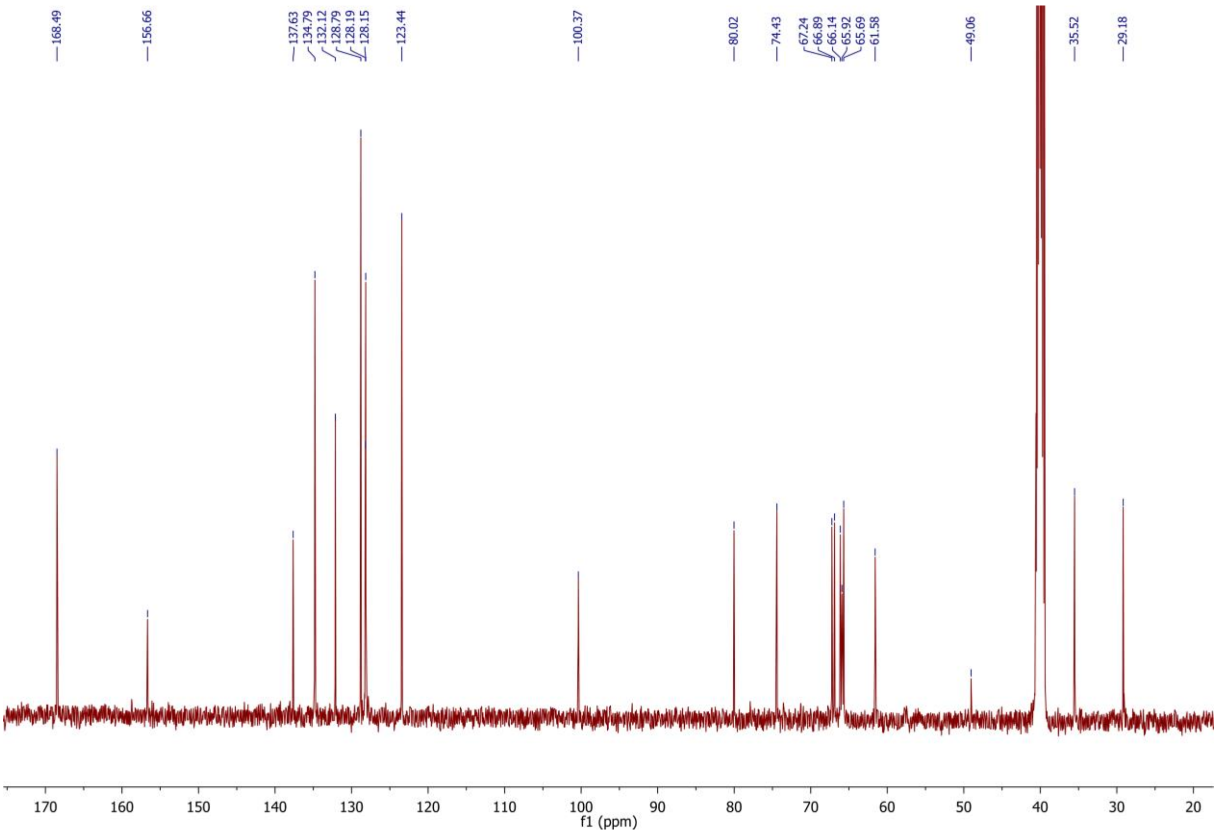

ESI-MS: **19**

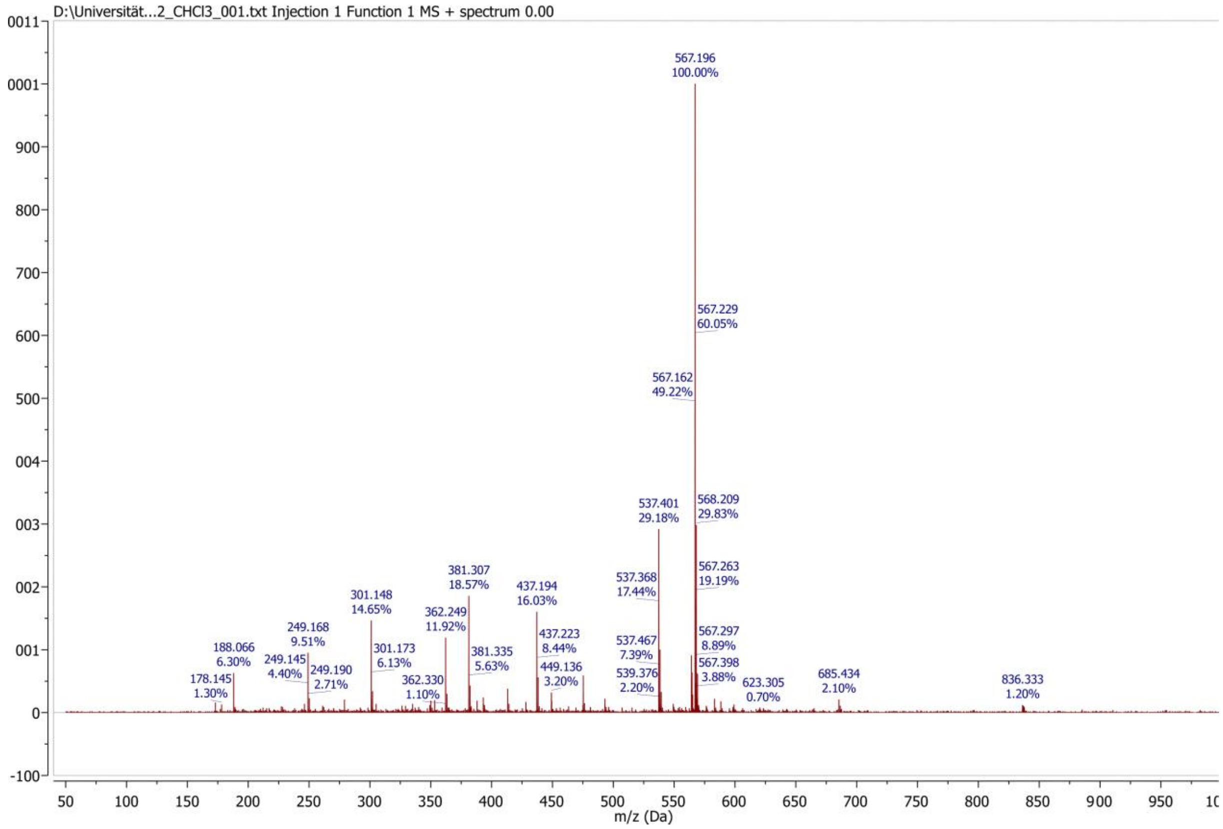
D:\Universität...2_CHCl3_001.txt Injection 1 Function 1 MS + spectrum 0.00
567.196
100.00%
567.229
60.05%
567.162
49.22%
537.401
29.18%
568.209
29.83%
381.307
18.57%
437.194
16.03%
537.368
17.44%
567.263
19.19%
301.148
14.65%
362.249
11.92%
249.168
9.51%
188.066
6.30%
249.145
4.40%
249.190
2.71%
178.145
1.30%
301.173
6.13%
362.330
1.10%
381.335
5.63%
437.223
8.44%
449.136
3.20%
537.467
7.39%
539.376
2.20%
567.297
8.89%
567.398
3.88%
623.305
0.70%
685.434
2.10%
836.333
1.20%
m/z (Da)

^{1}H and ^{13}C NMR spectra (**20**)

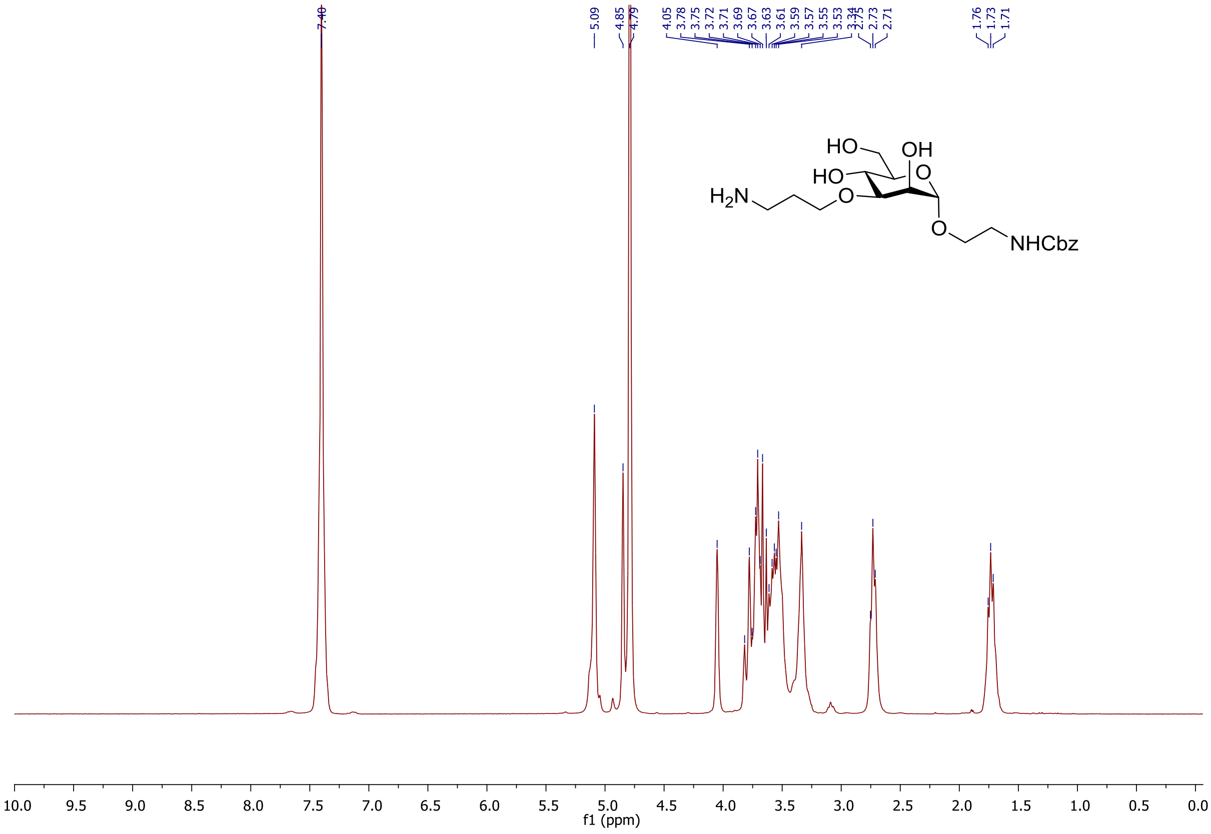

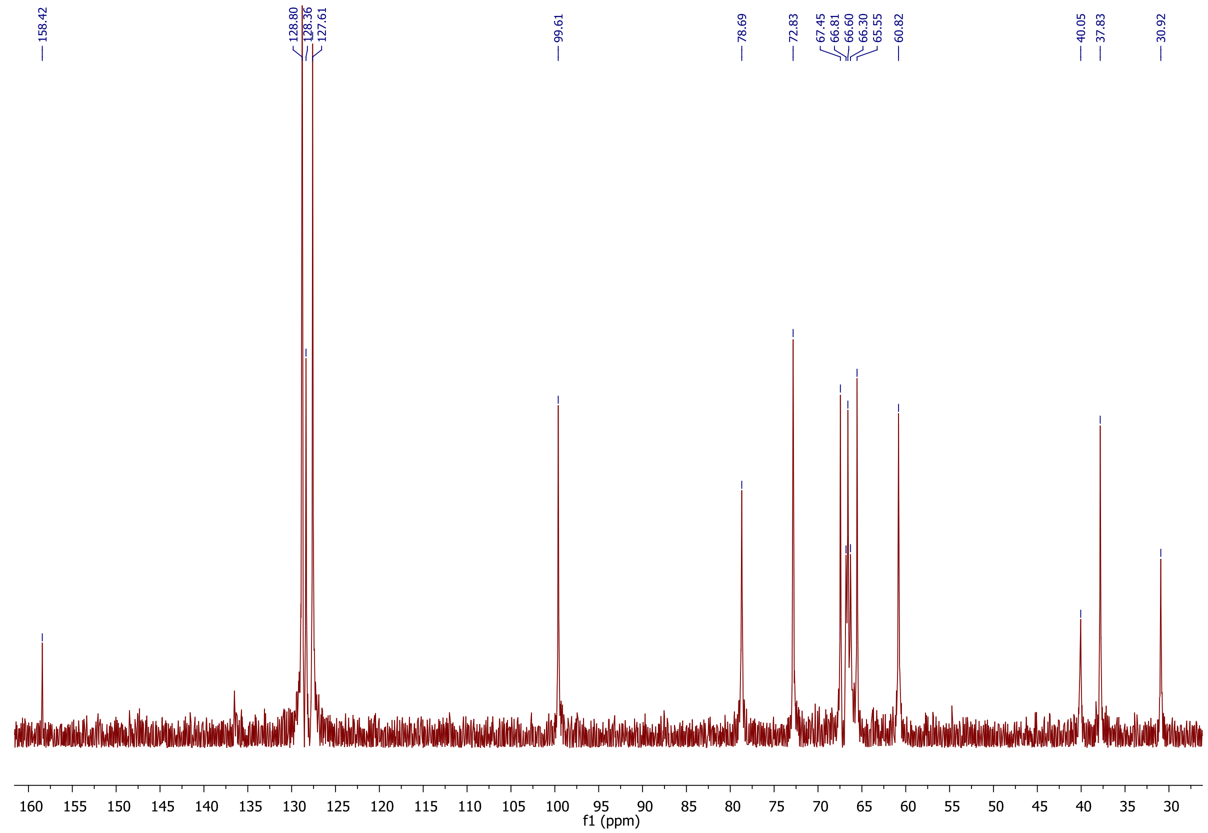

^{1}H and ^{13}C NMR spectra (**22**)

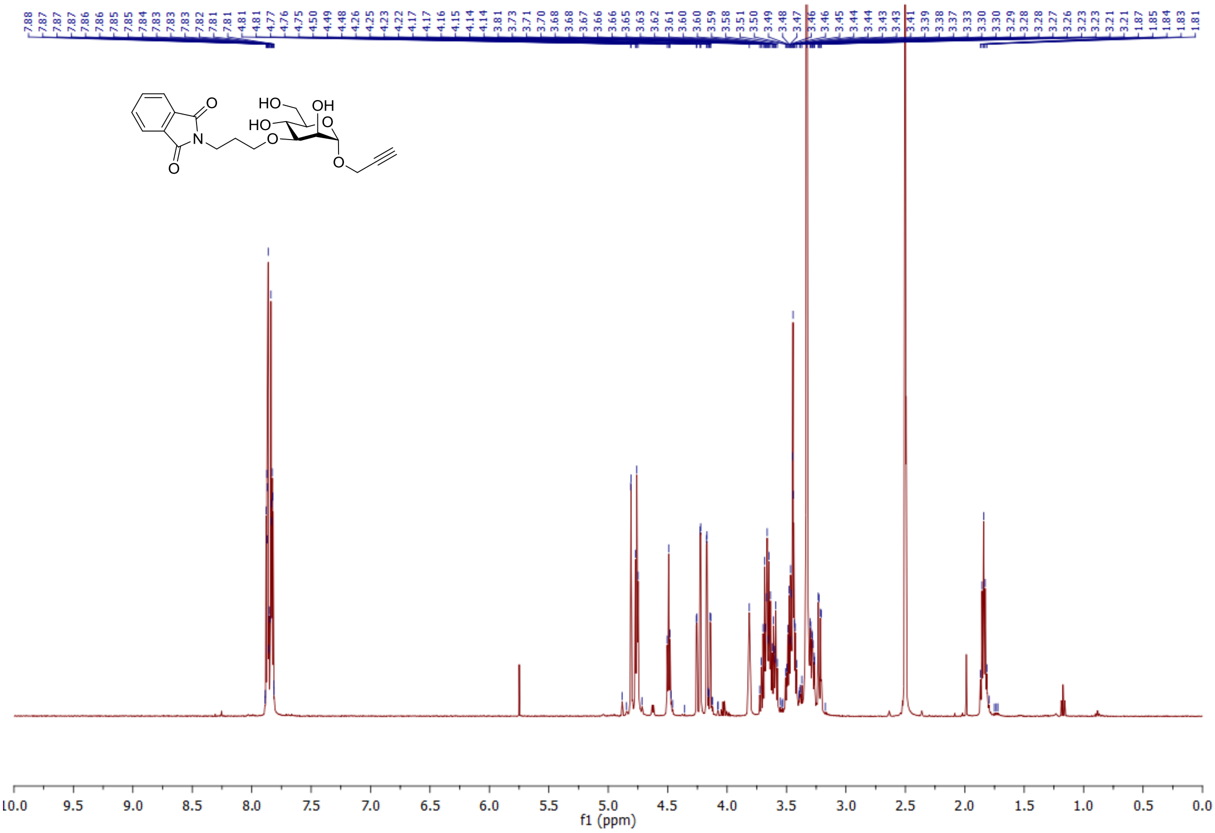

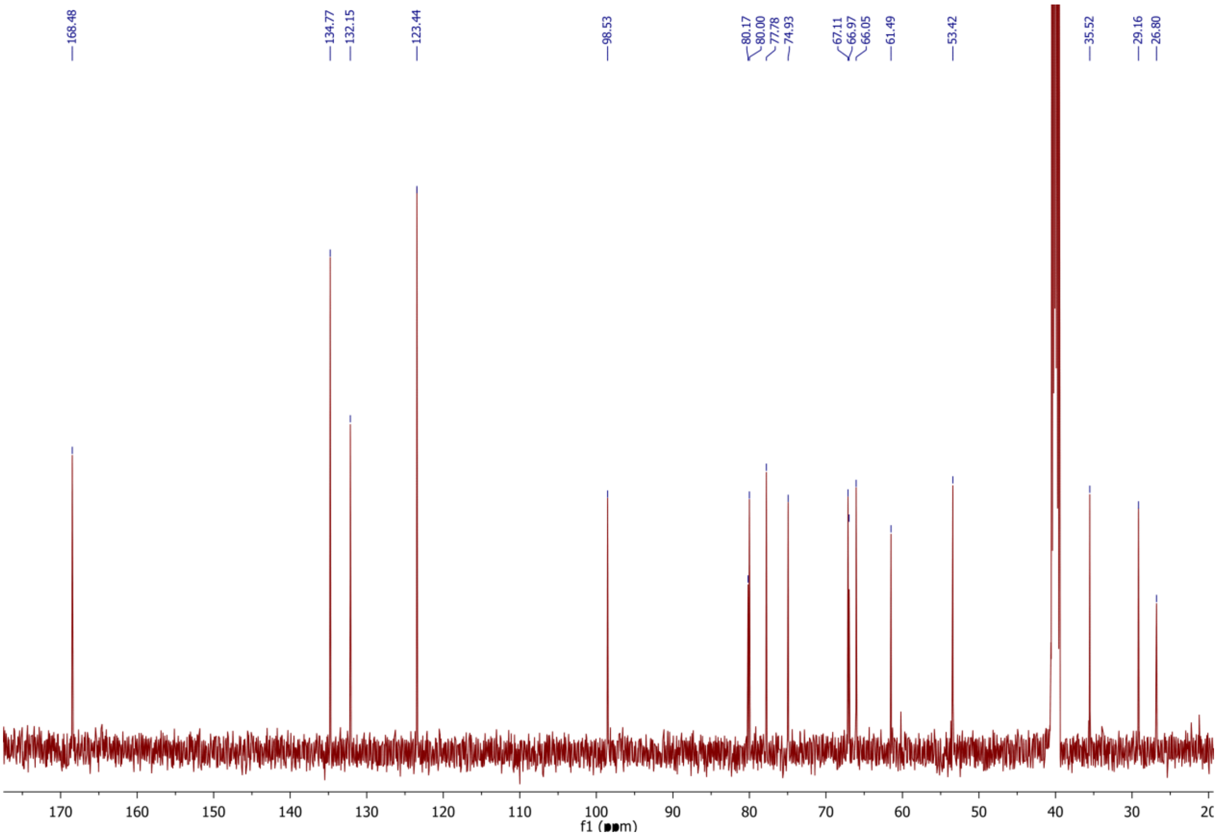

ESI-MS: **22**

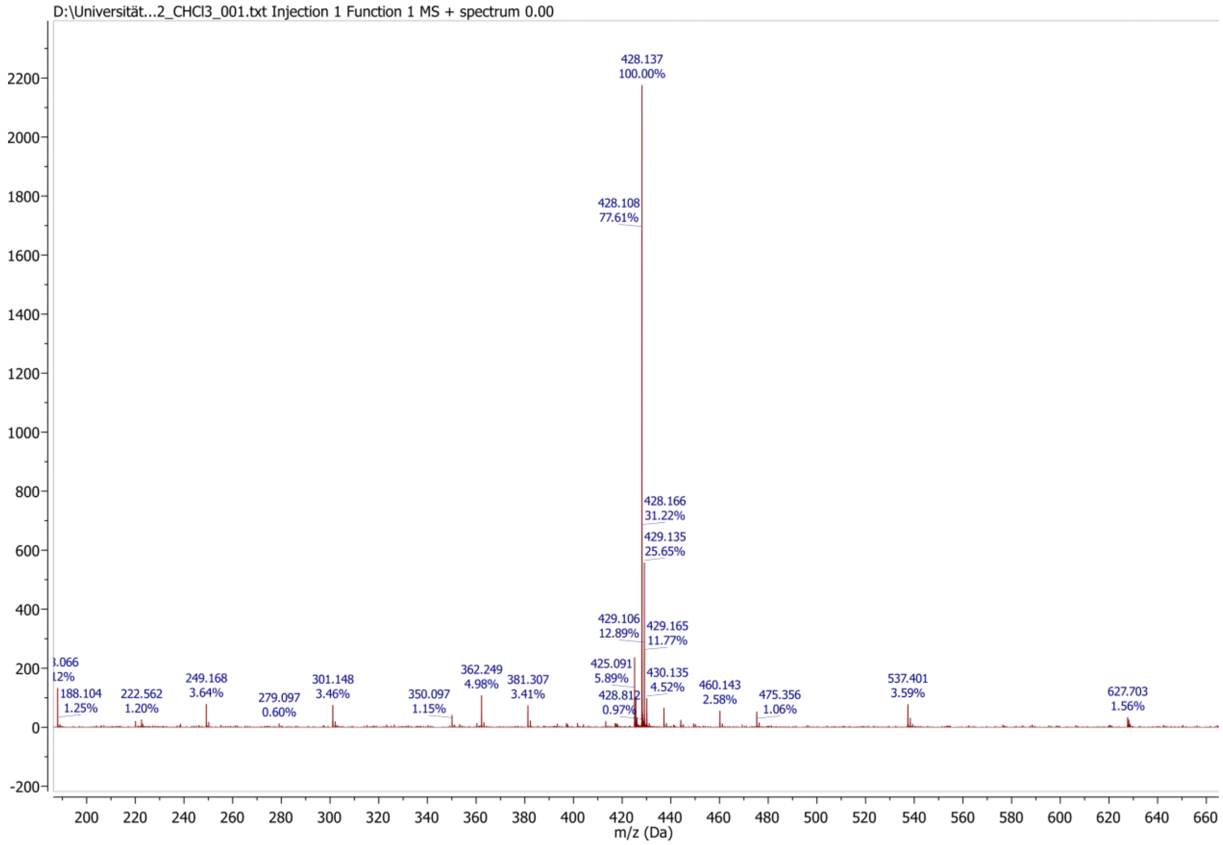
D:\Universität...2_CHCl3_001.txt Injection 1 Function 1 MS + spectrum 0.00
428.137
100.00%
428.108
77.61%
428.166
31.22%
429.135
25.65%
429.106
12.89%
429.165
11.77%
425.091
5.89%
430.135
4.52%
428.812
0.97%
.066
12%
188.104
1.25%
222.562
1.20%
249.168
3.64%
279.097
0.60%
301.148
3.46%
350.097
1.15%
362.249
4.98%
381.307
3.41%
460.143
2.58%
475.356
1.06%
537.401
3.59%
627.703
1.56%
m/z (Da)

^{1}H and ^{13}C NMR spectra (**23**)

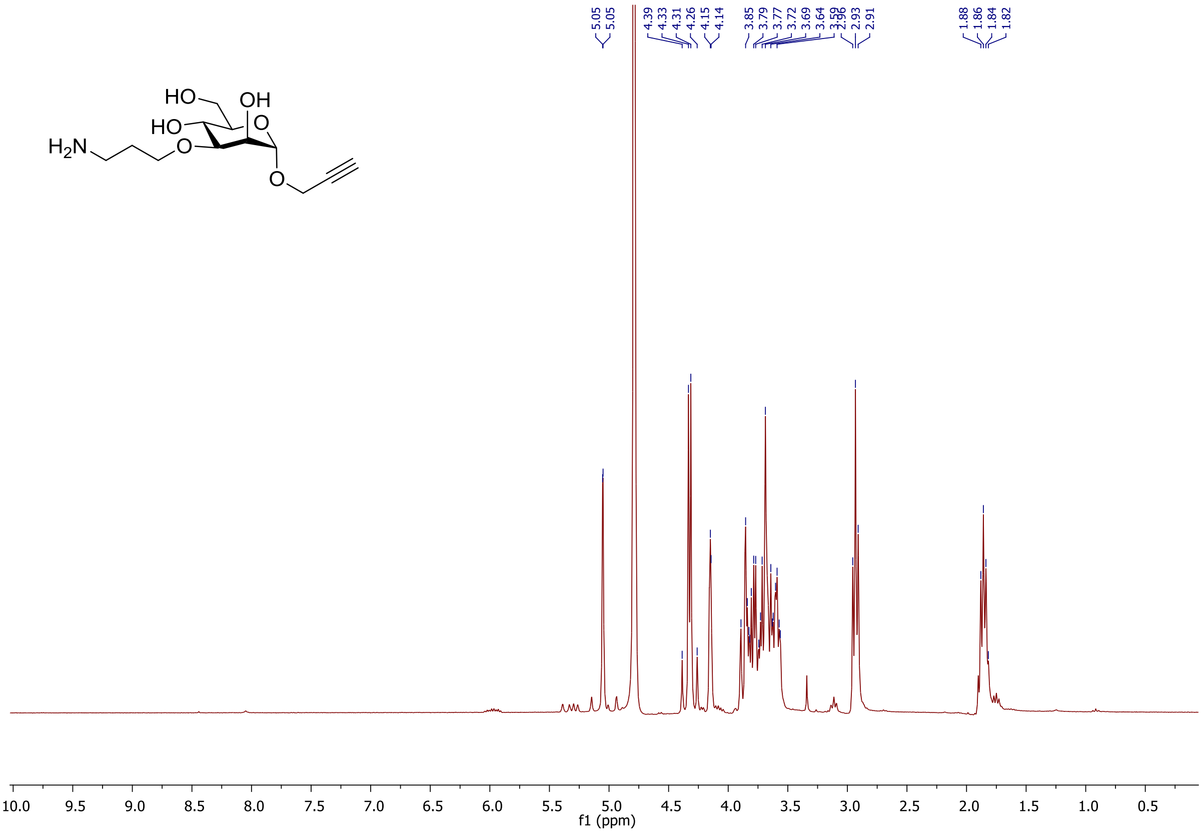

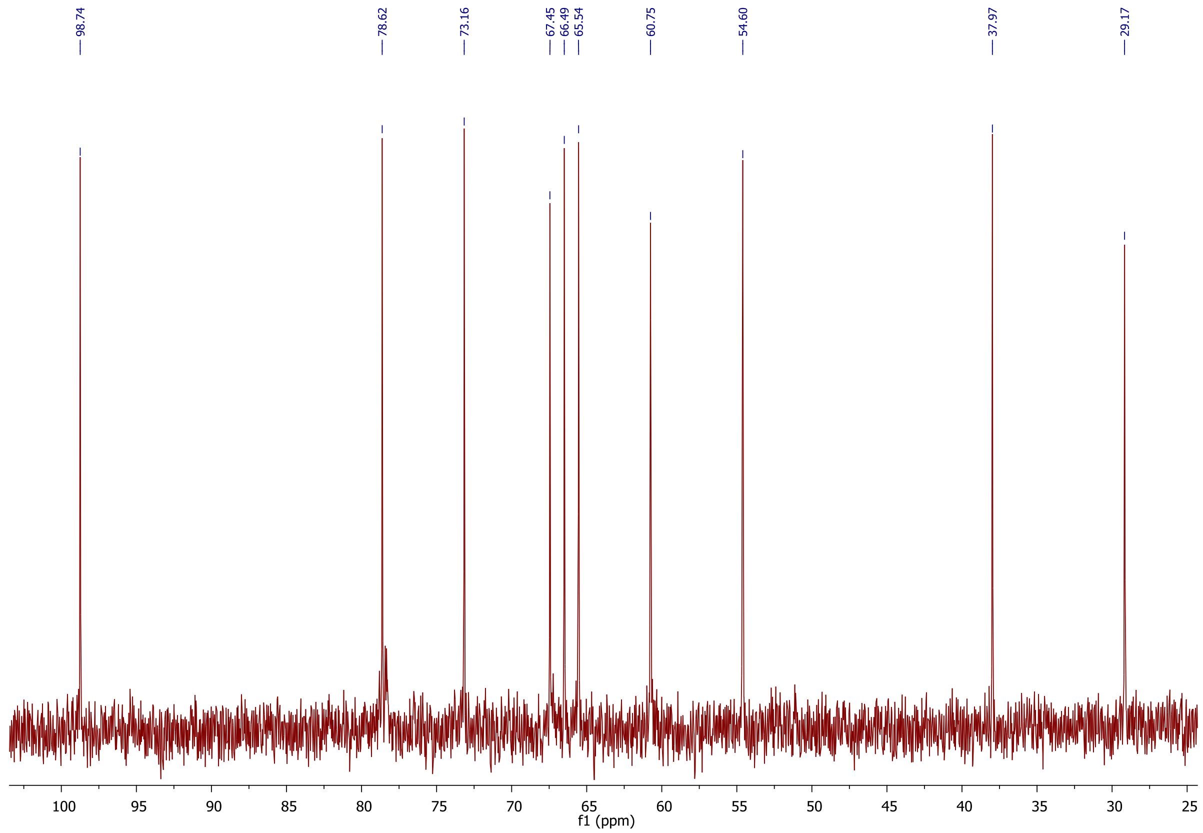

^{1}H and ^{13}C NMR spectra (**25**)

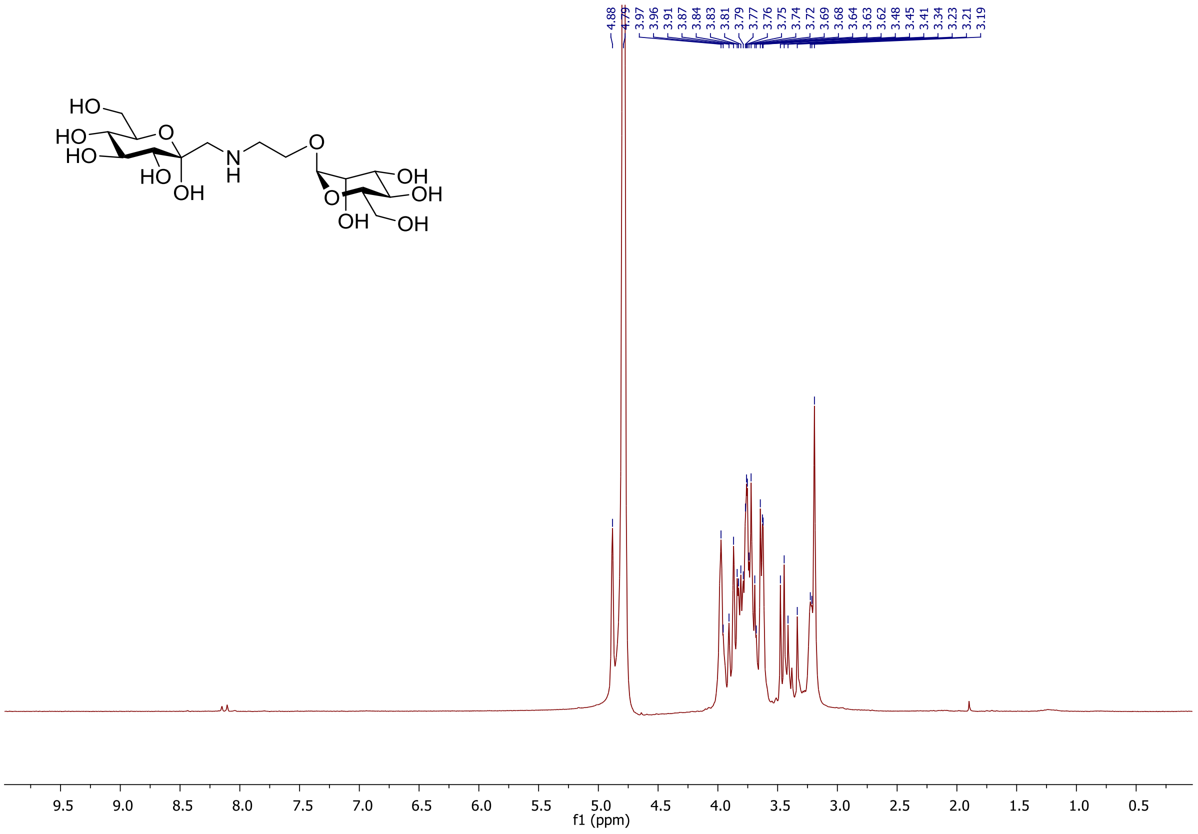

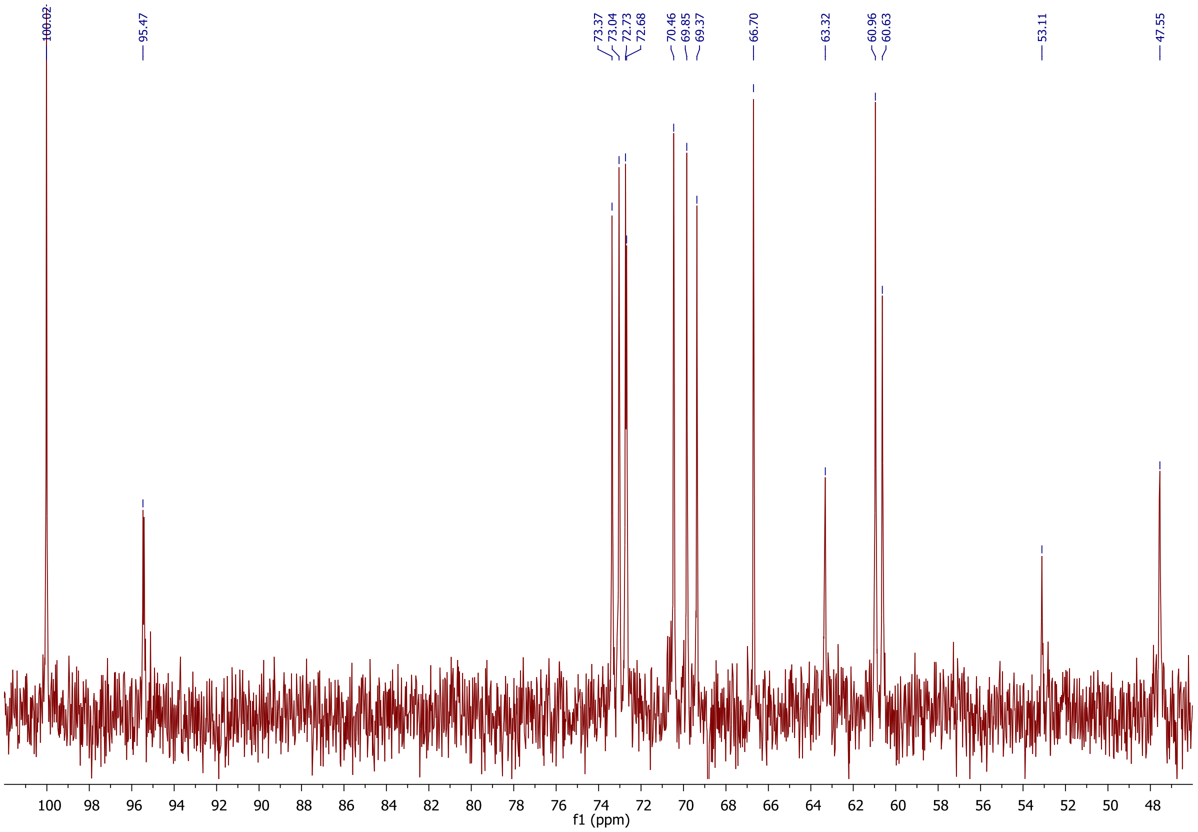

HRMS (MALDI): **25**

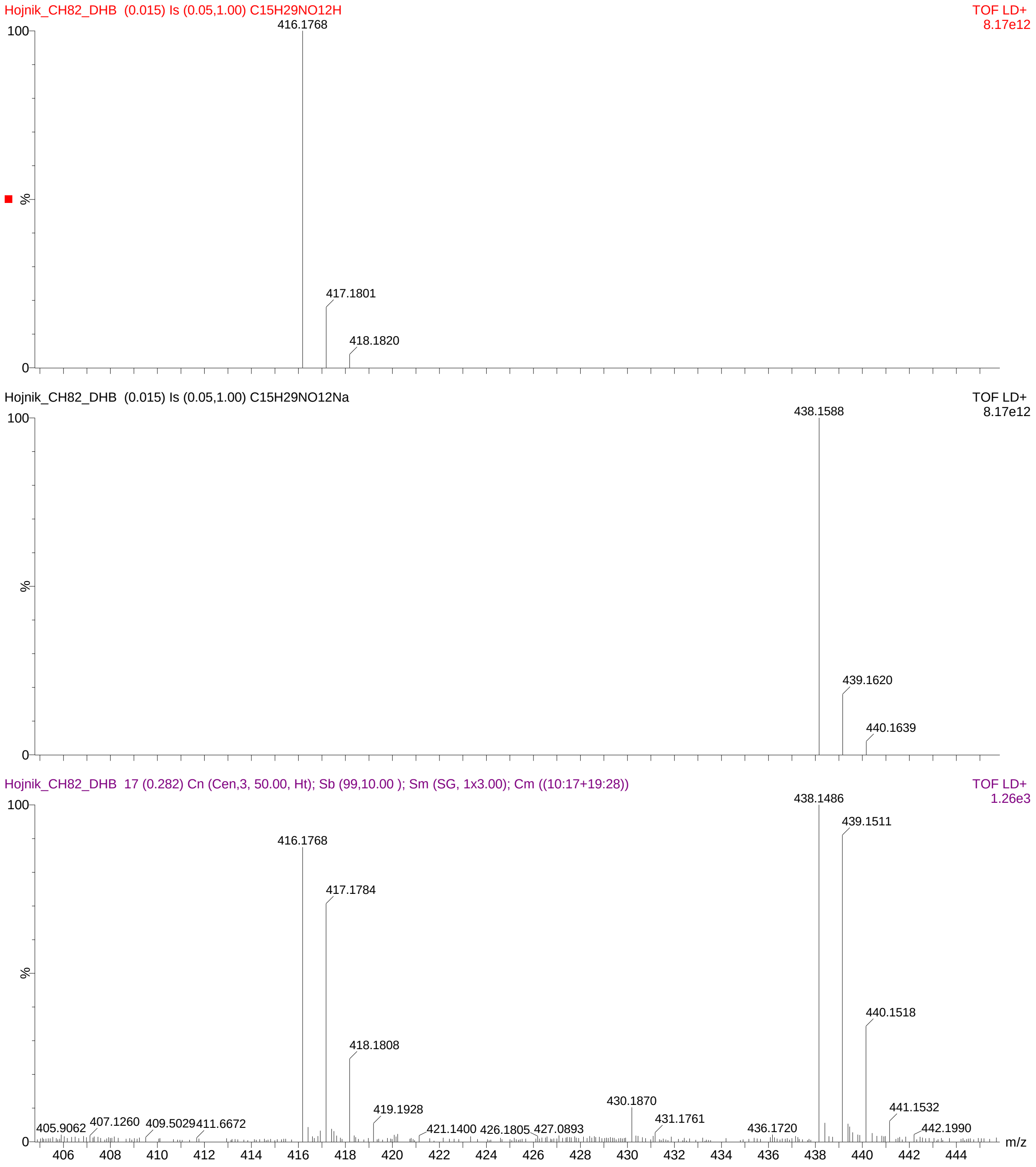

^{1}H and ^{13}C NMR spectra (**26**)

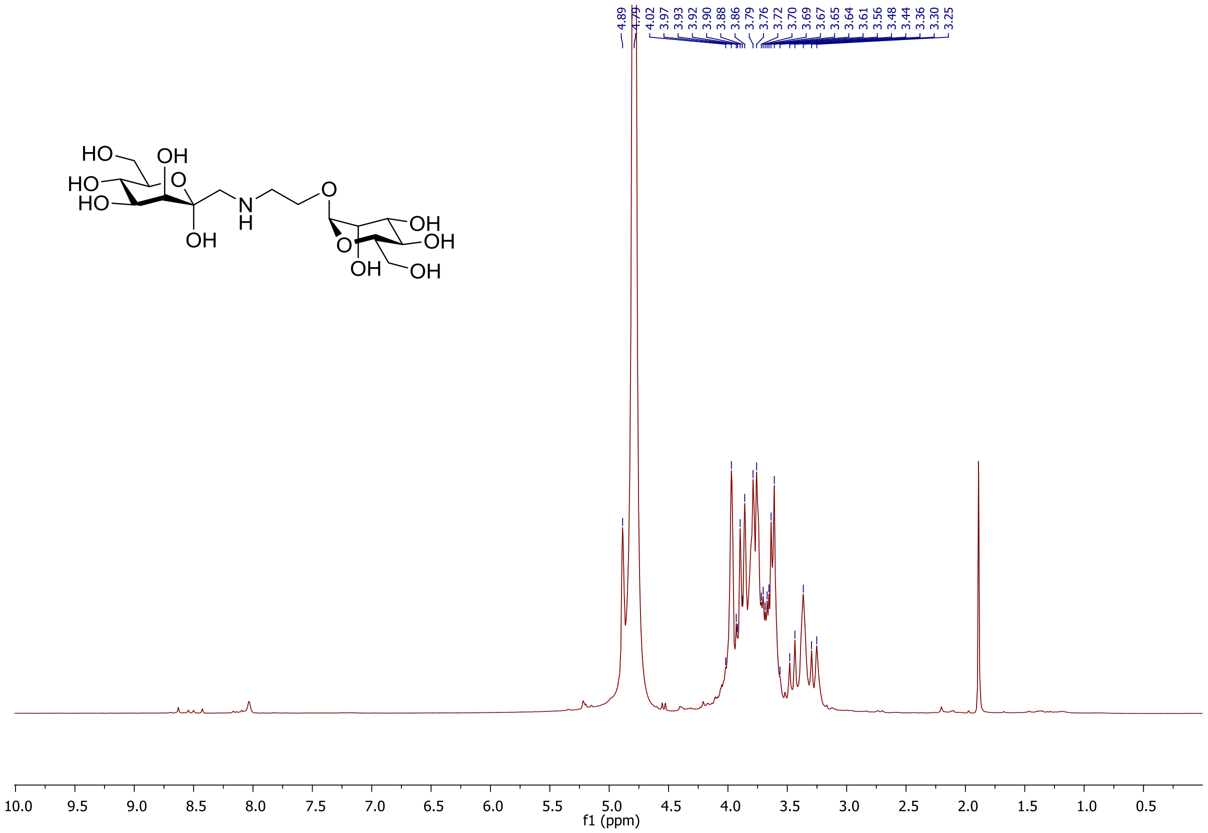

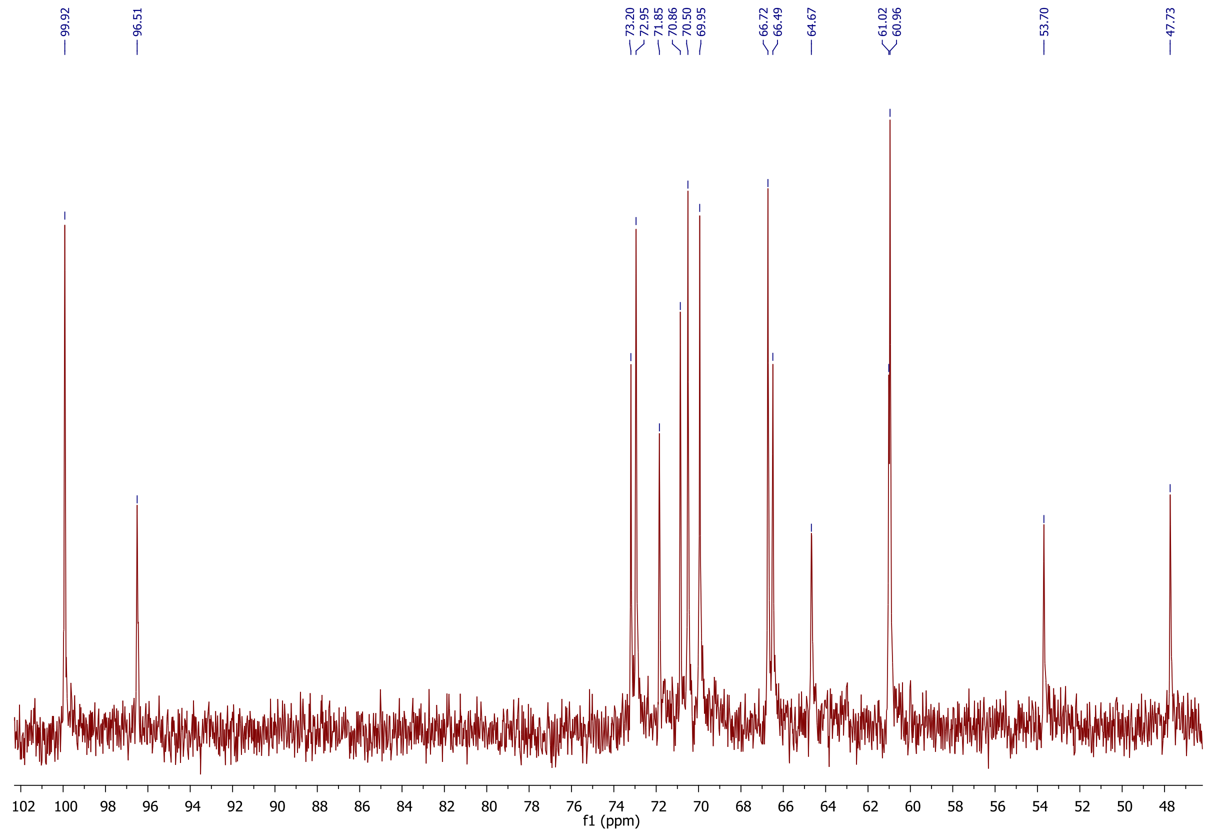

HRMS (MALDI): **26**

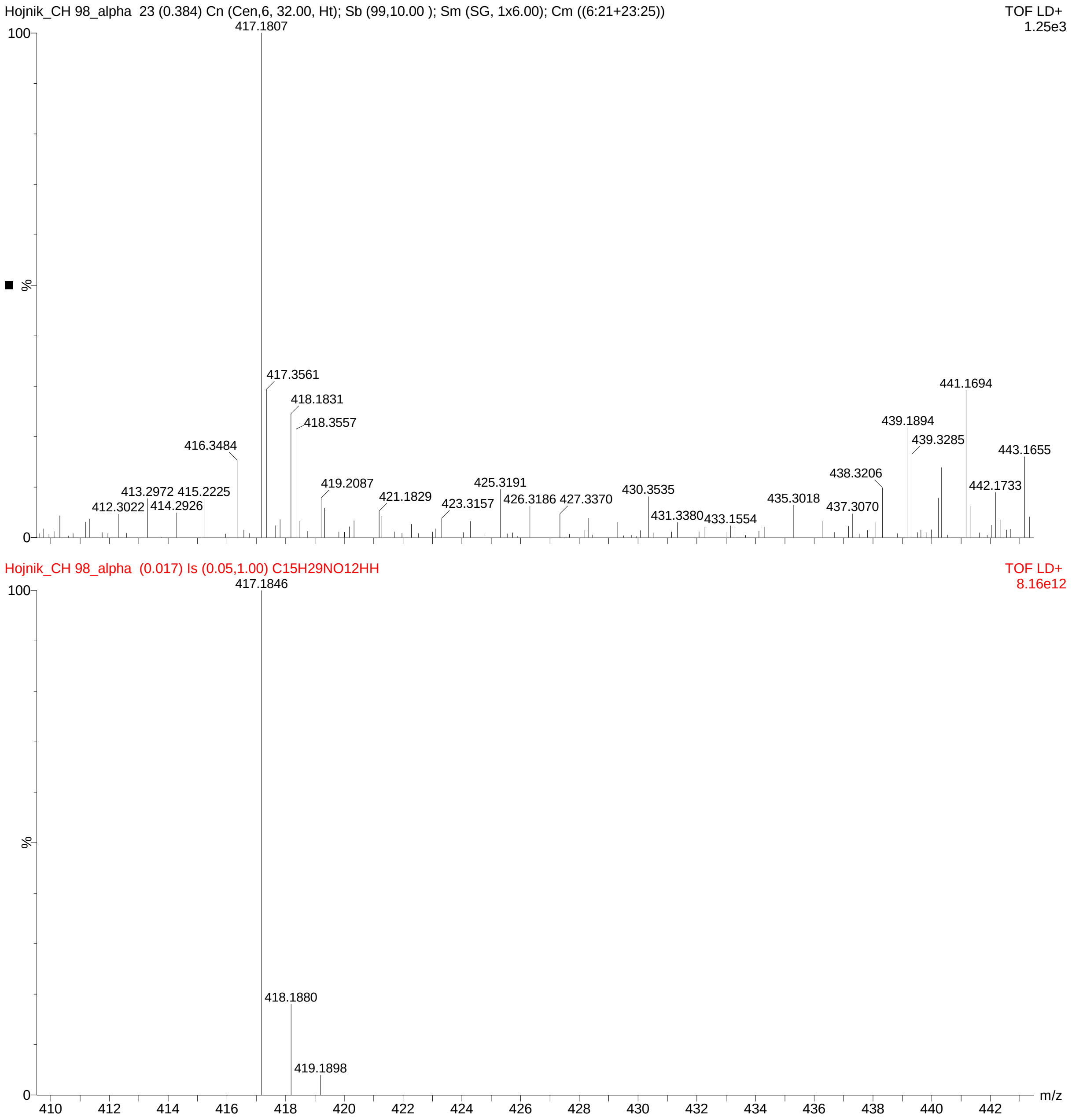

^{1}H and ^{13}C NMR spectra (**27**)

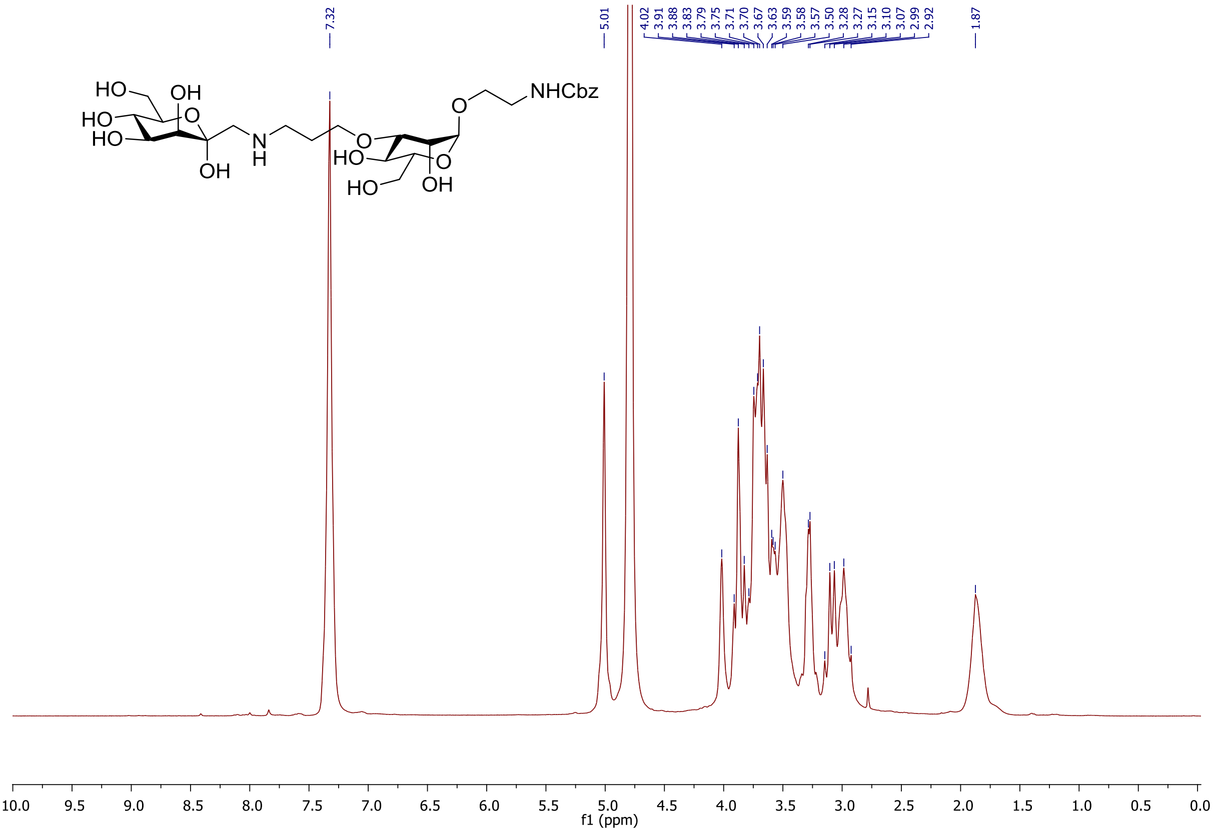

pH=7

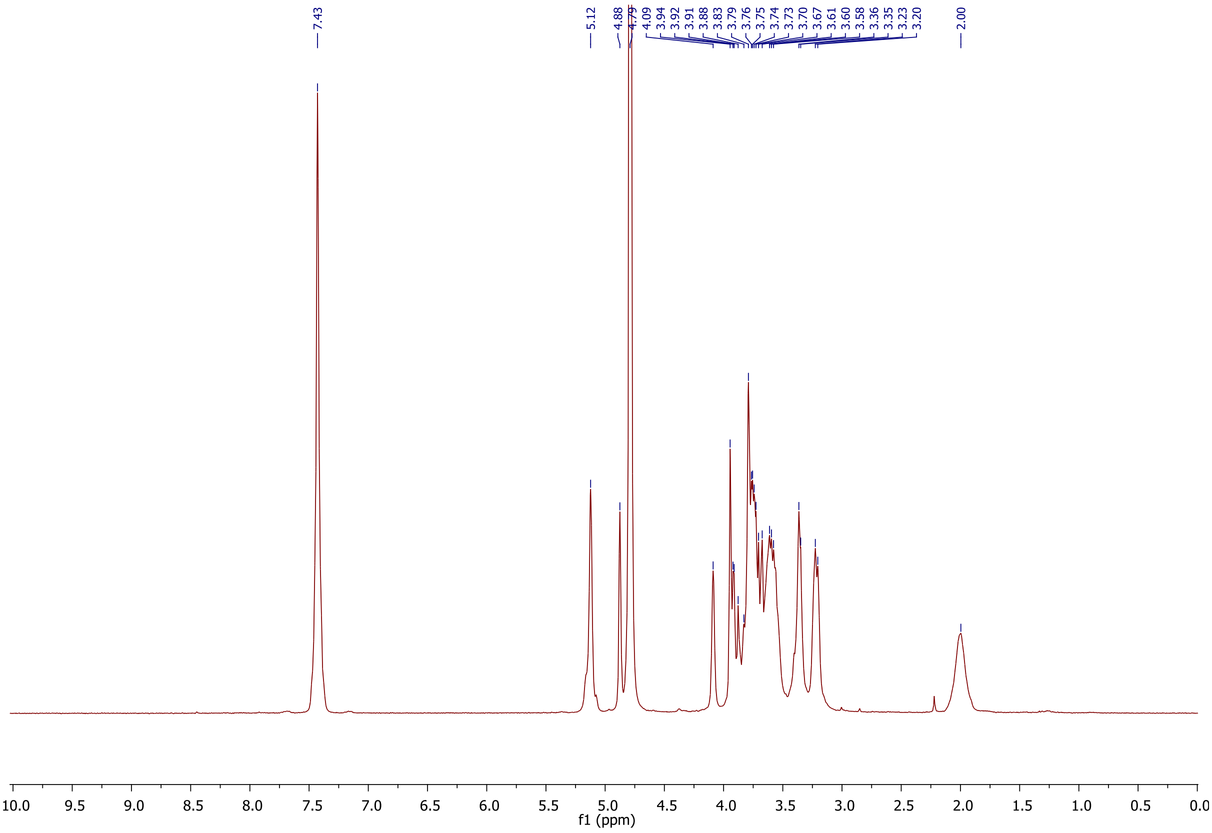

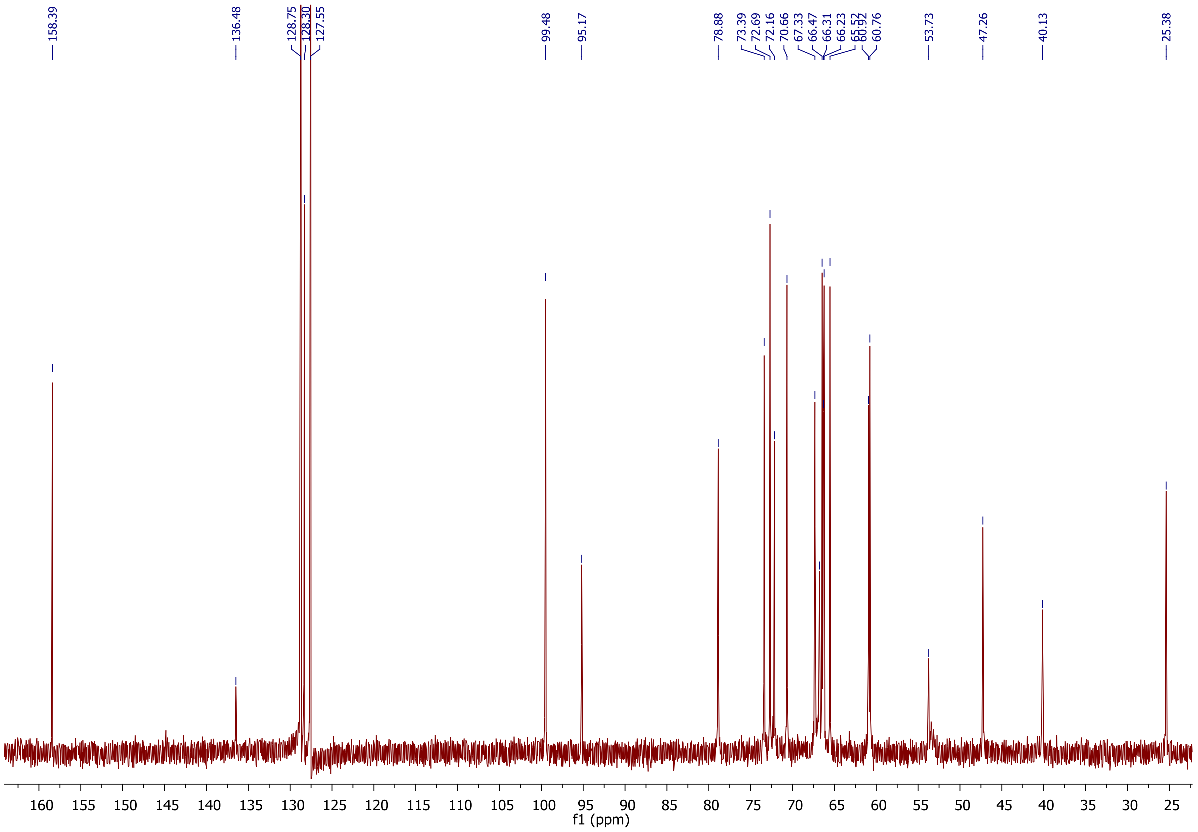
158.39
136.48
128.75
128.30
127.55
99.48
95.17
78.88
73.39
72.69
72.16
70.66
67.33
66.47
66.31
66.23
65.52
60.92
60.76
53.73
47.26
40.13
25.38
f1 (ppm)

HRMS (MALDI): **27**

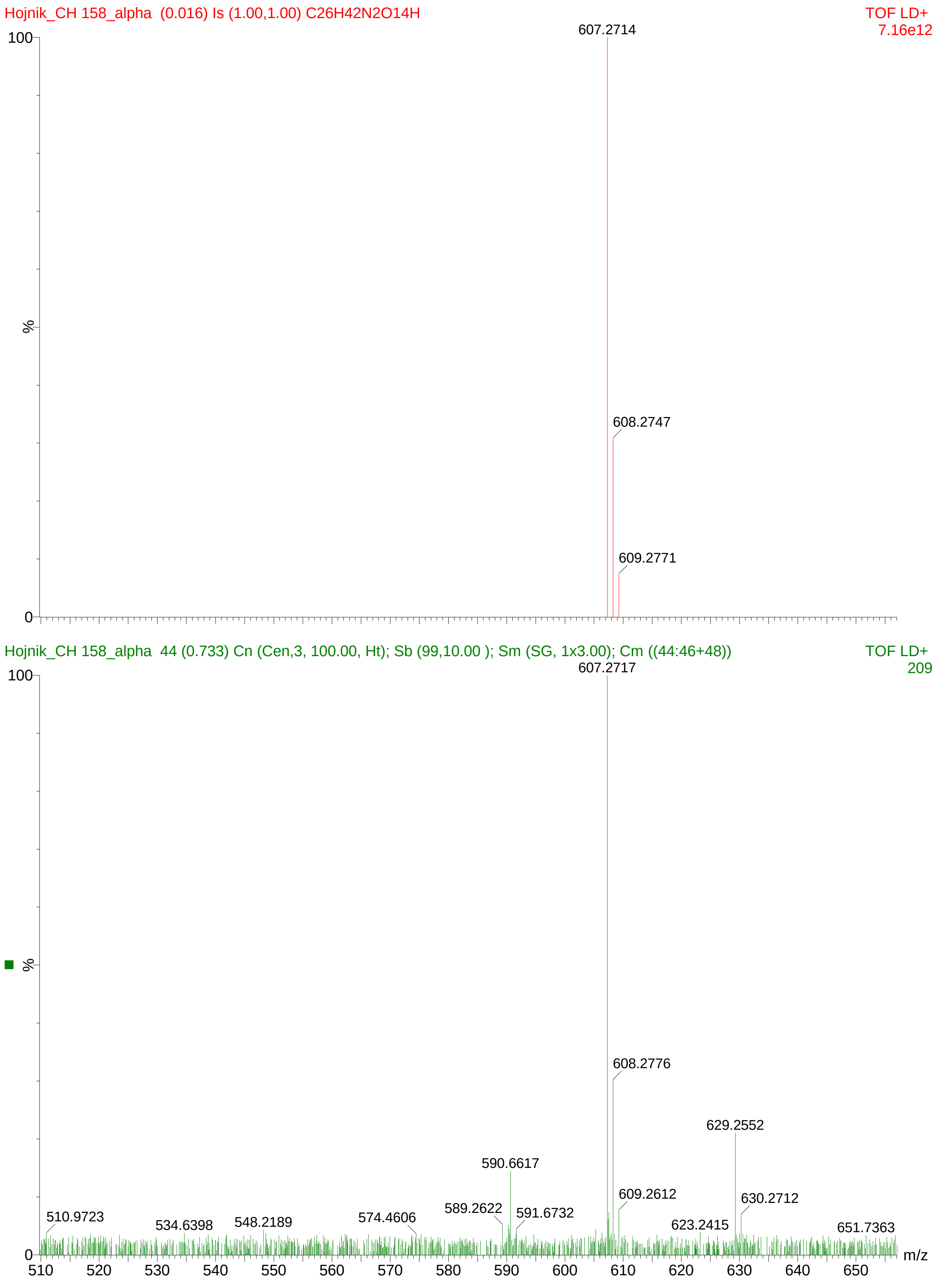

^{1}H and ^{13}C NMR spectra (**28**)

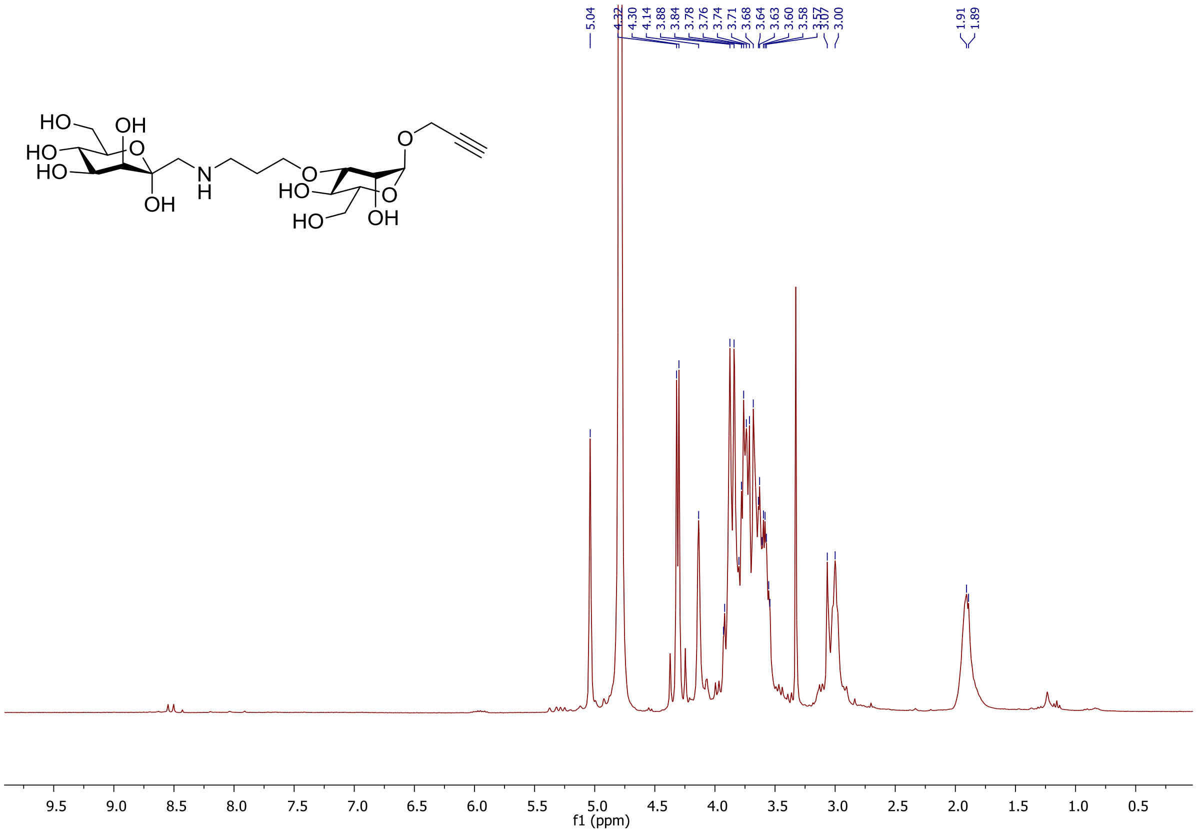

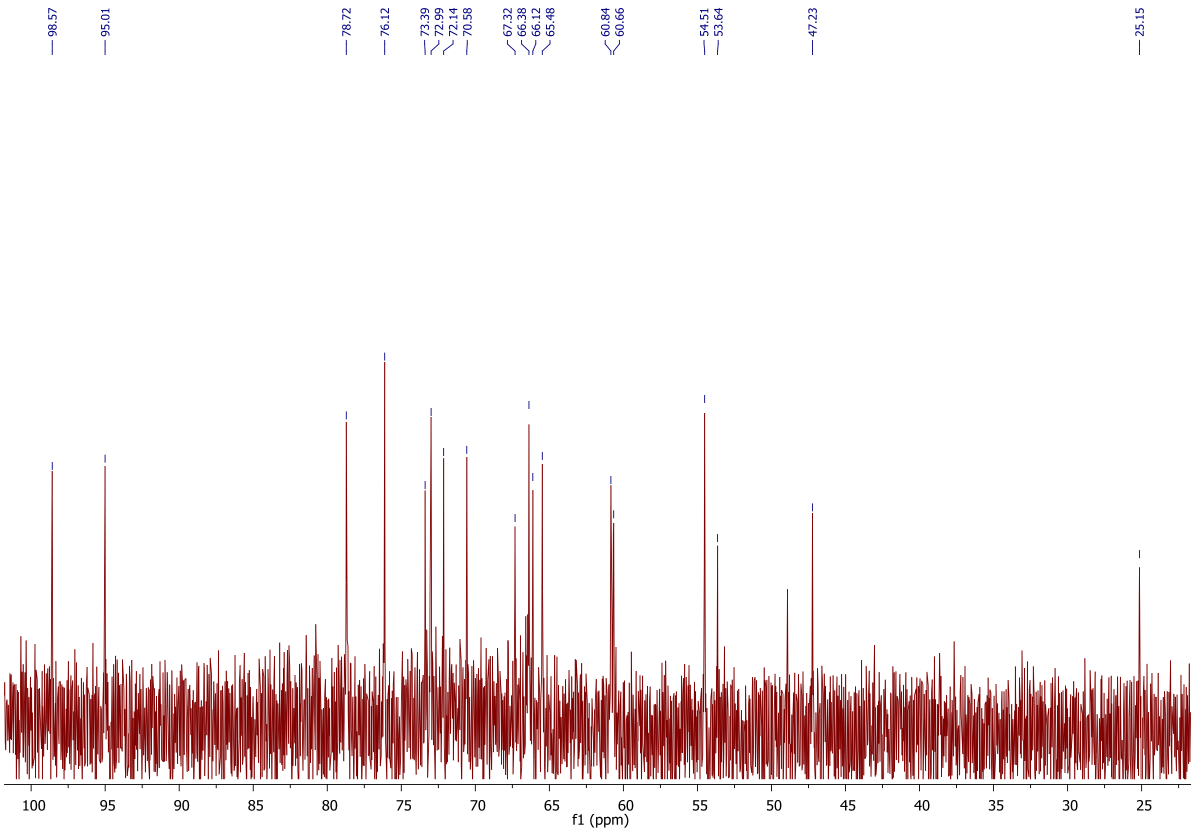

HRMS (MALDI): **28**

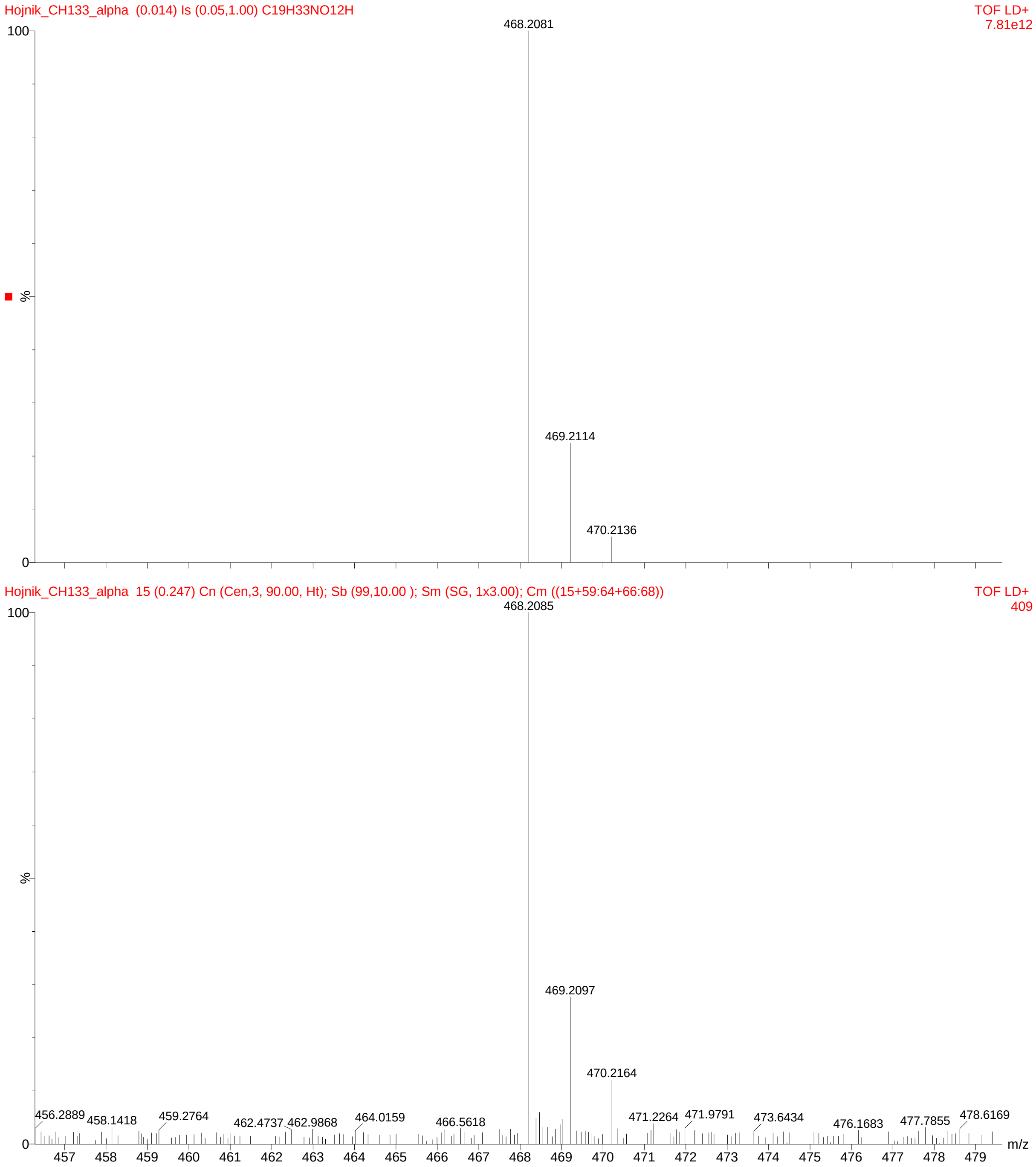

^{1}H and ^{13}C NMR spectra (**30**)

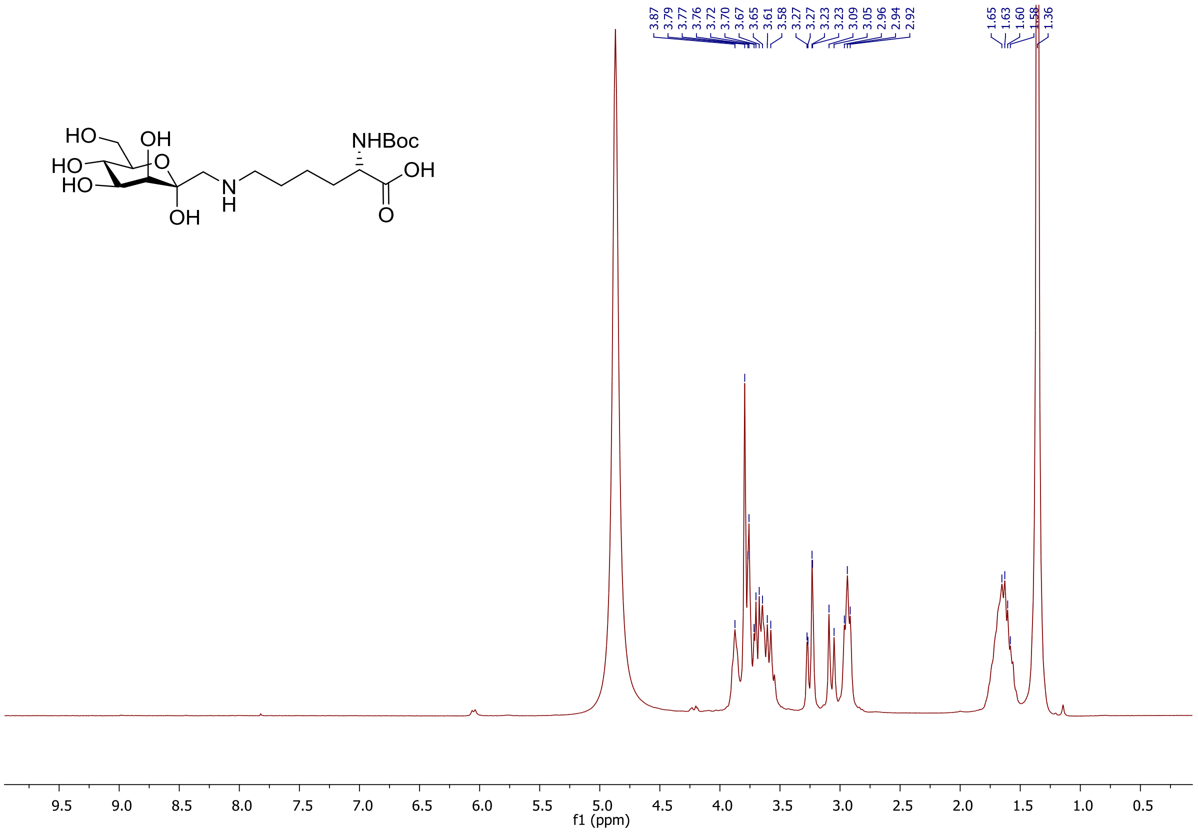

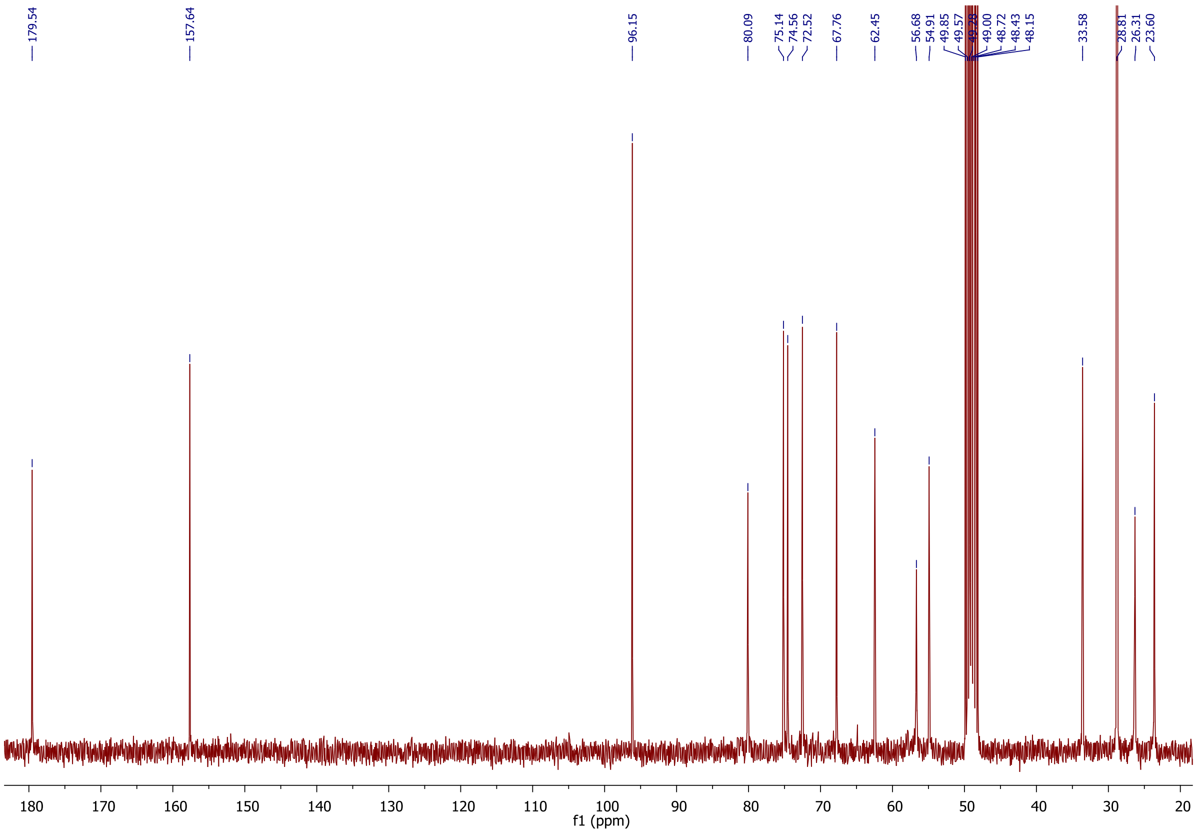

HRMS (MALDI): **30**

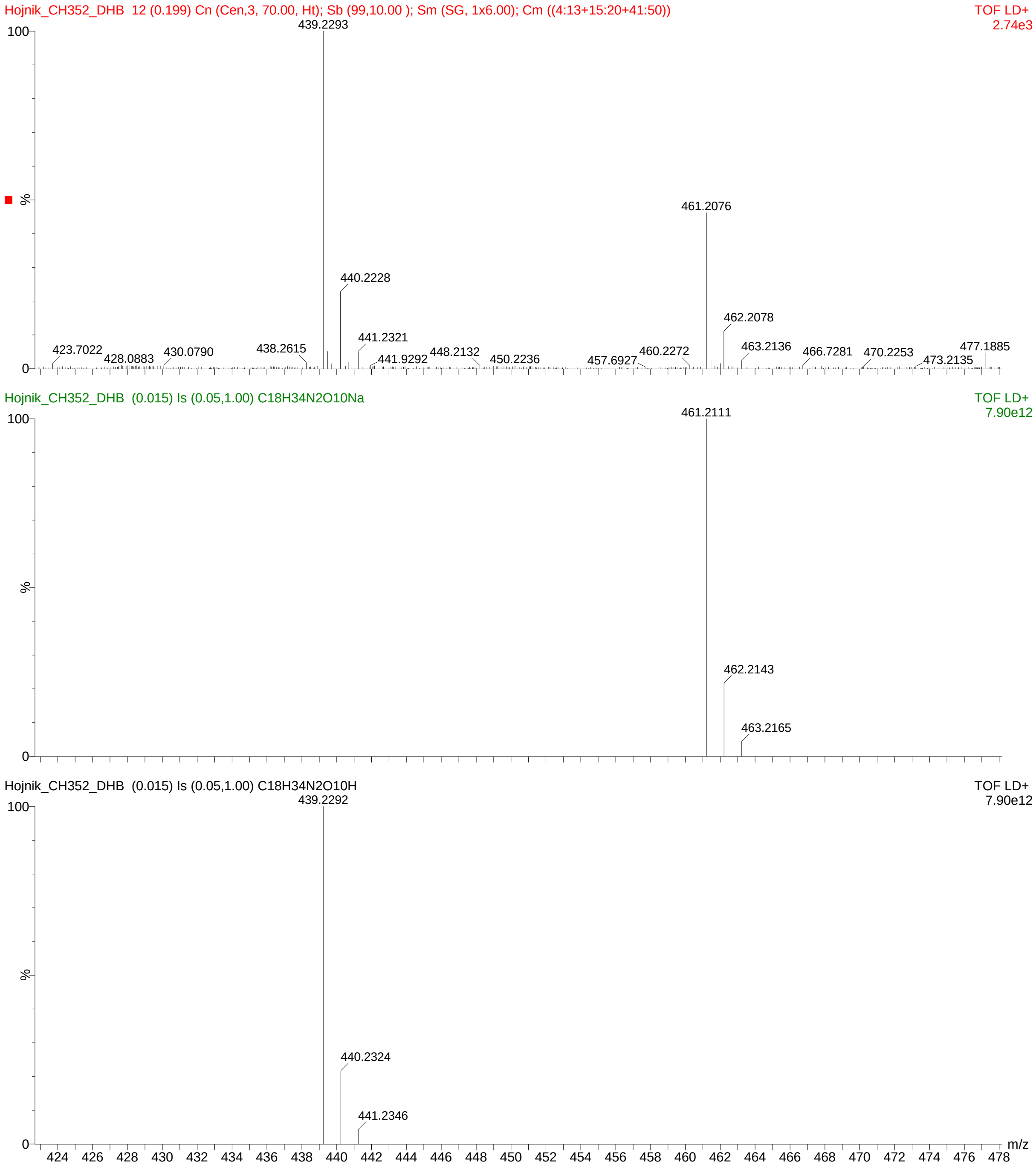

^{1}H and ^{13}C NMR spectra (**32**)

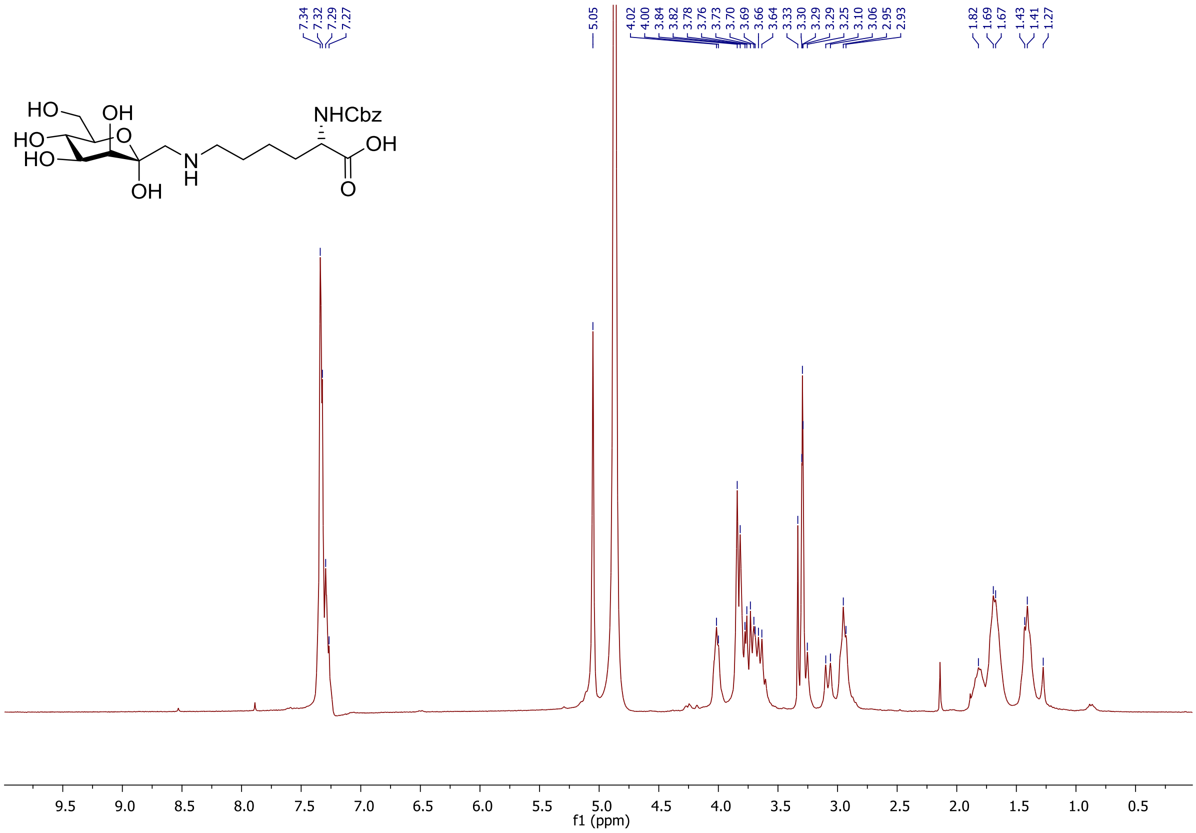

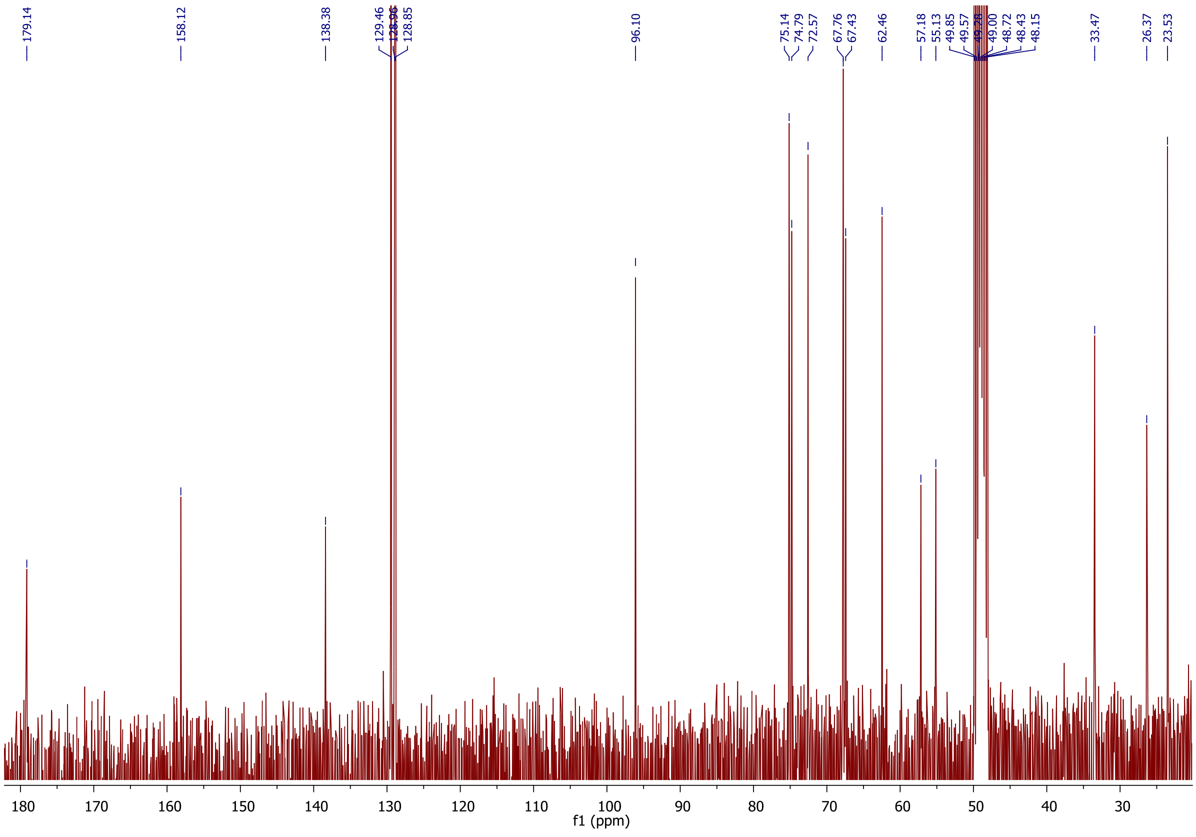

HRMS (MALDI): **32**

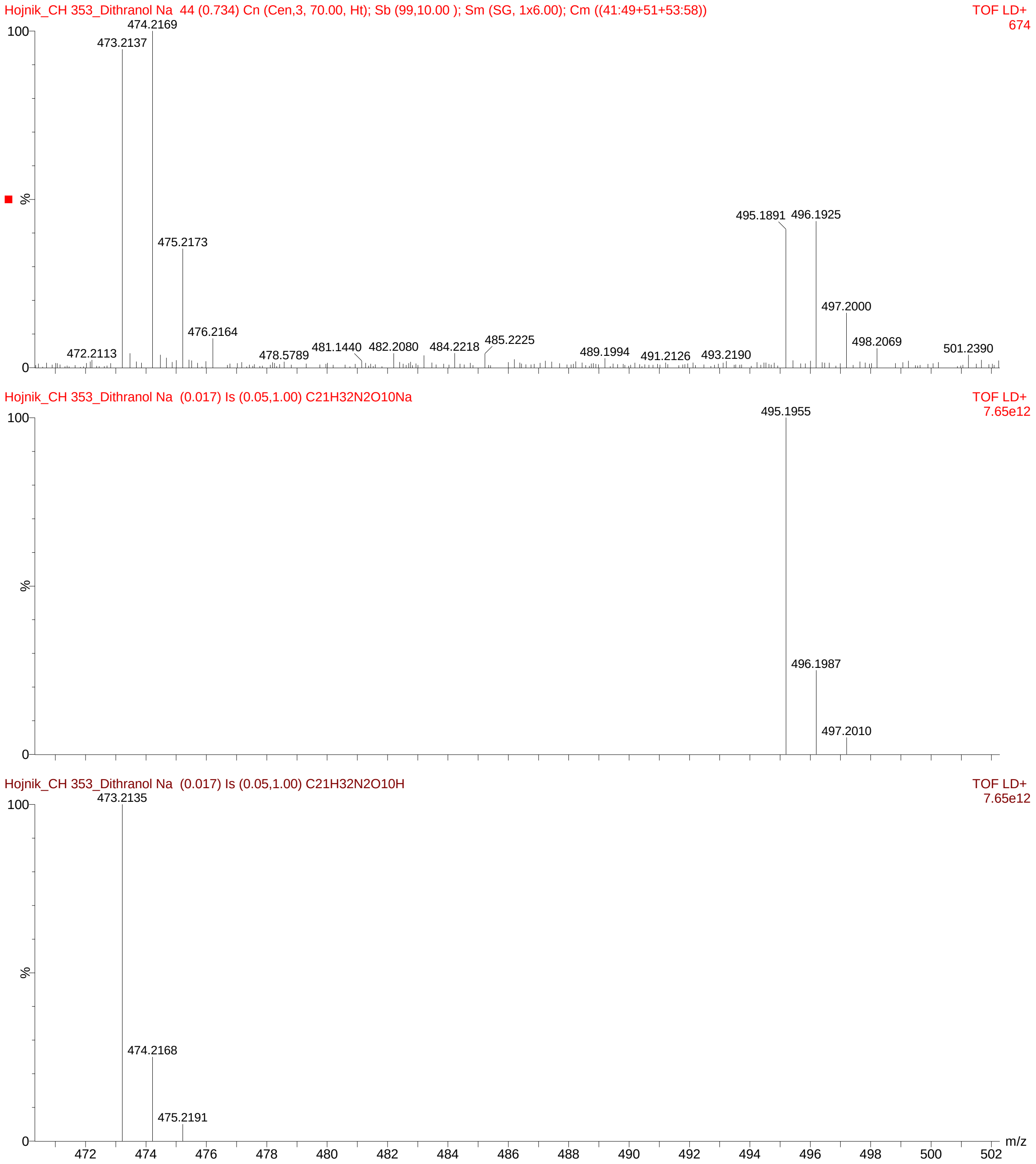

^{1}H and ^{13}C NMR spectra (**34**)

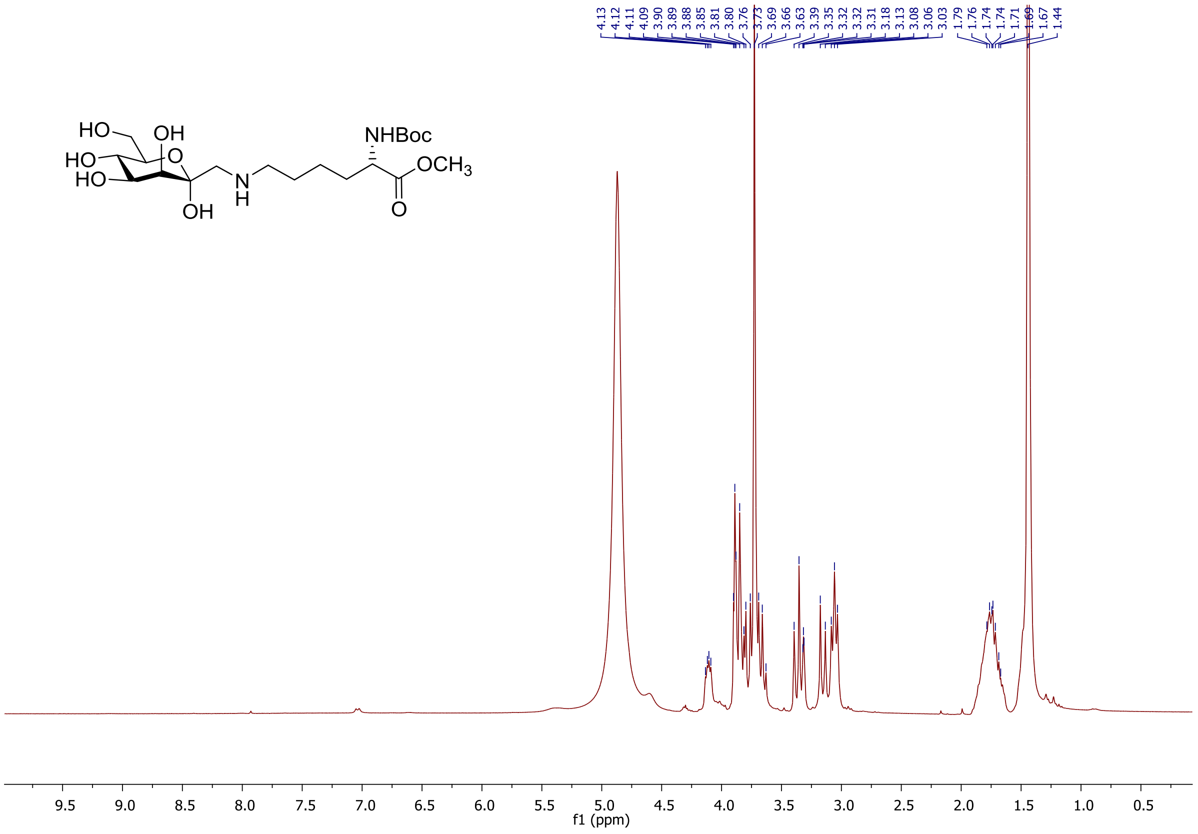

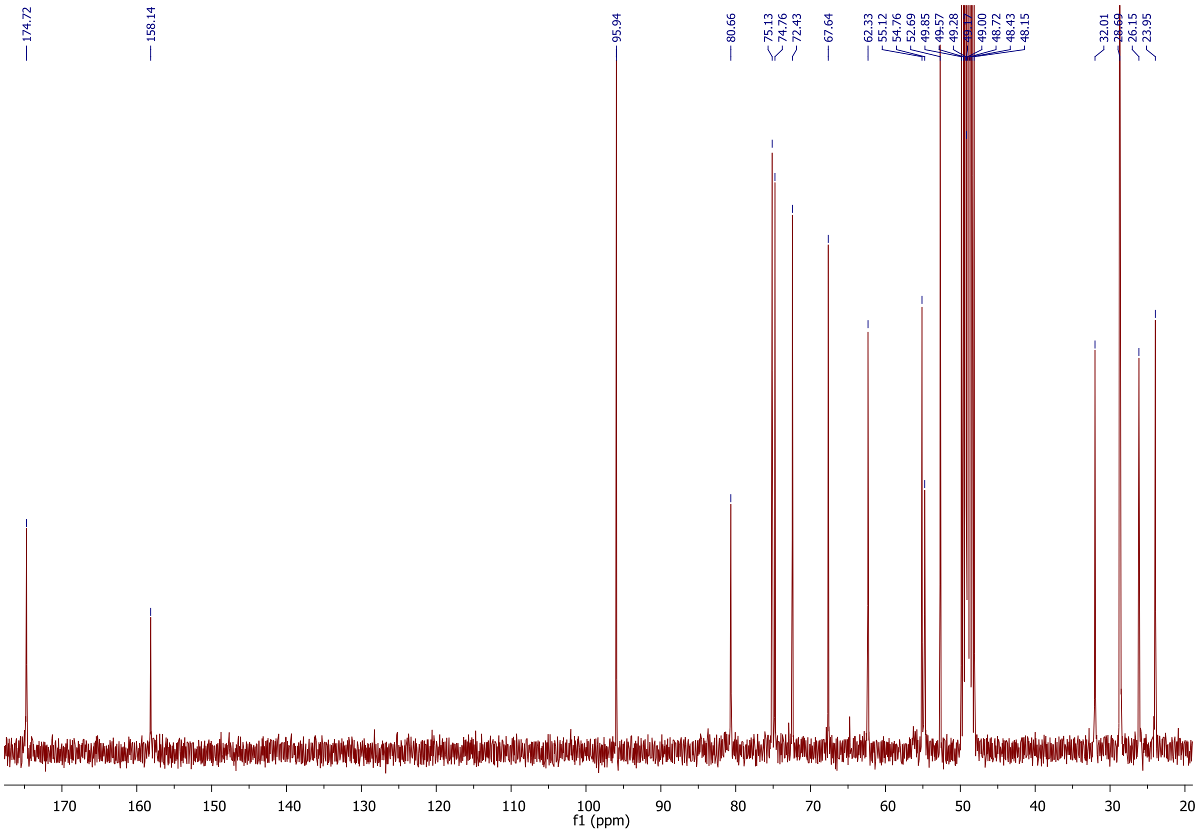

HRMS (MALDI): **34**

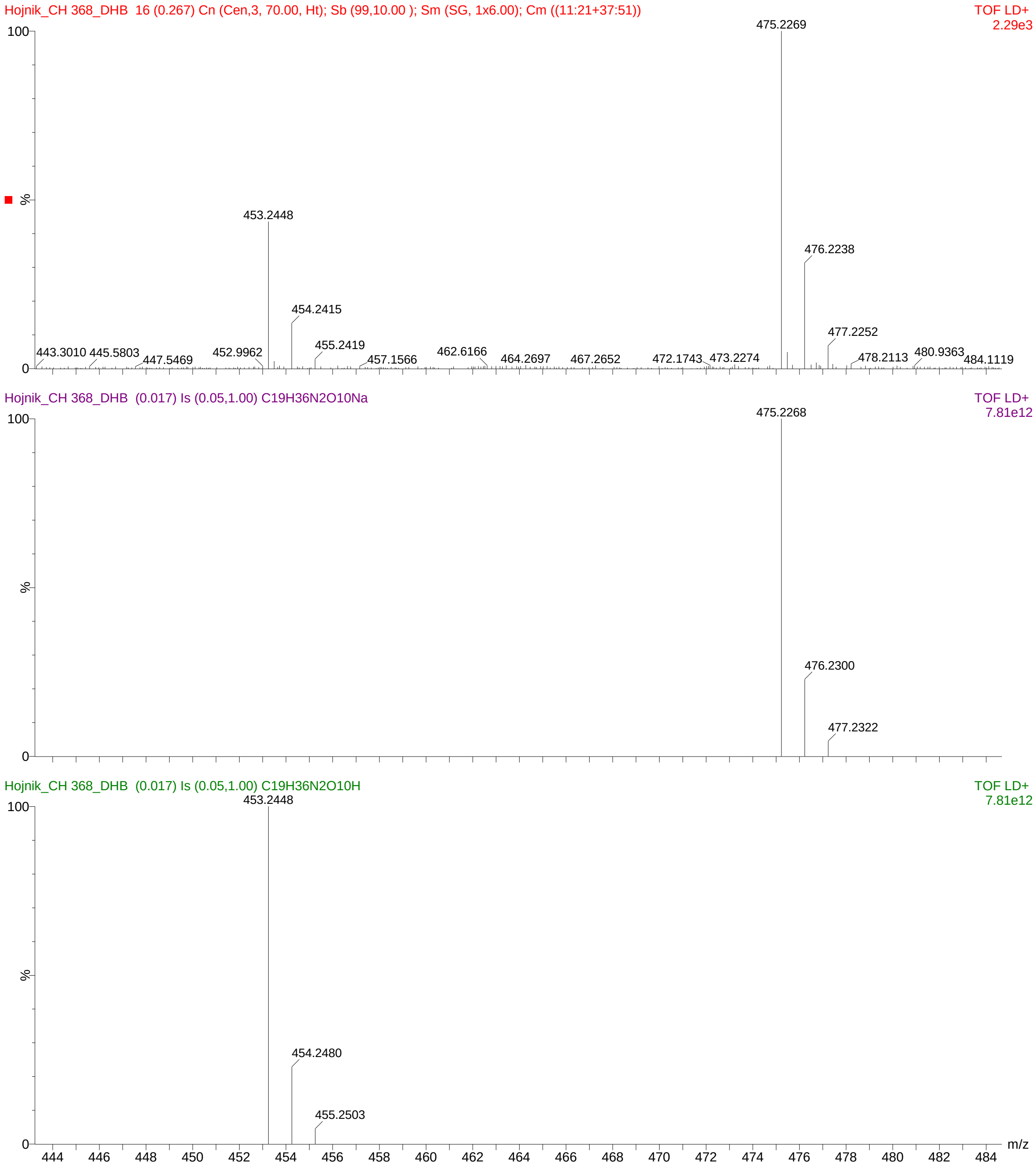

^{1}H and ^{13}C NMR spectra (**37**)

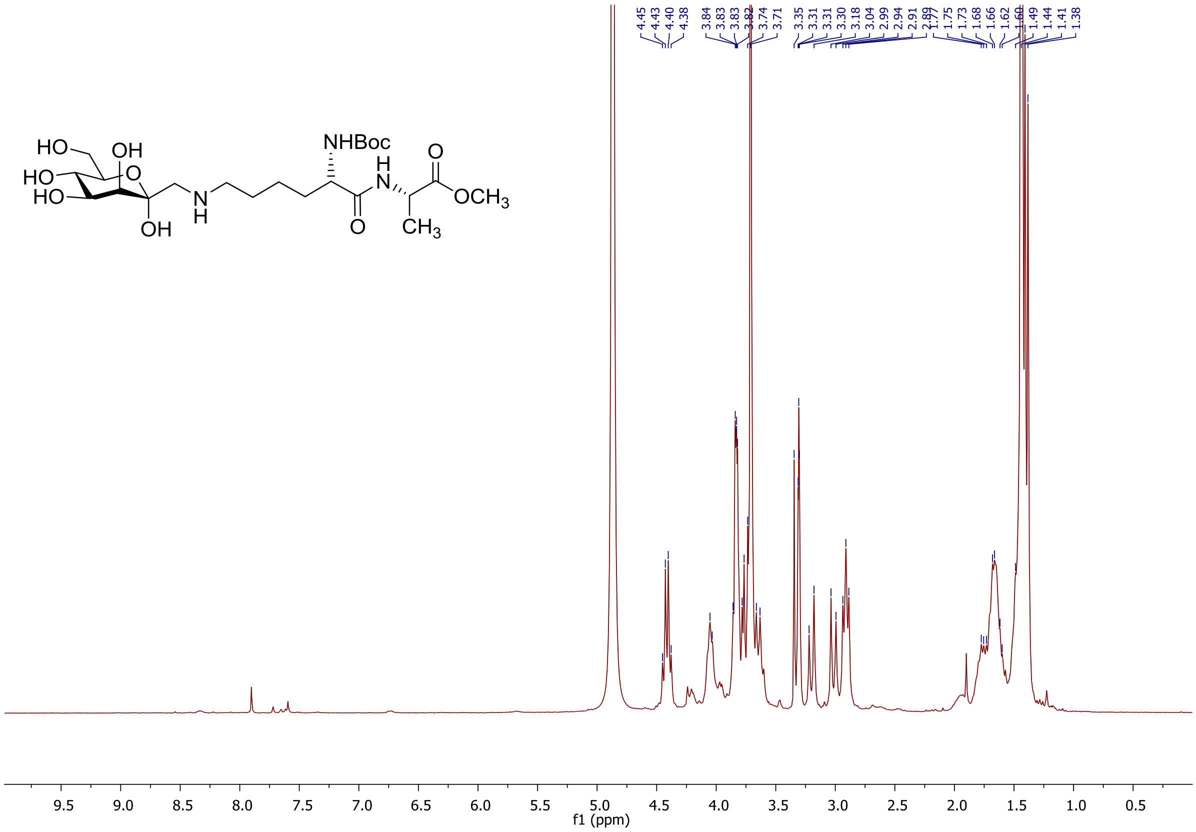

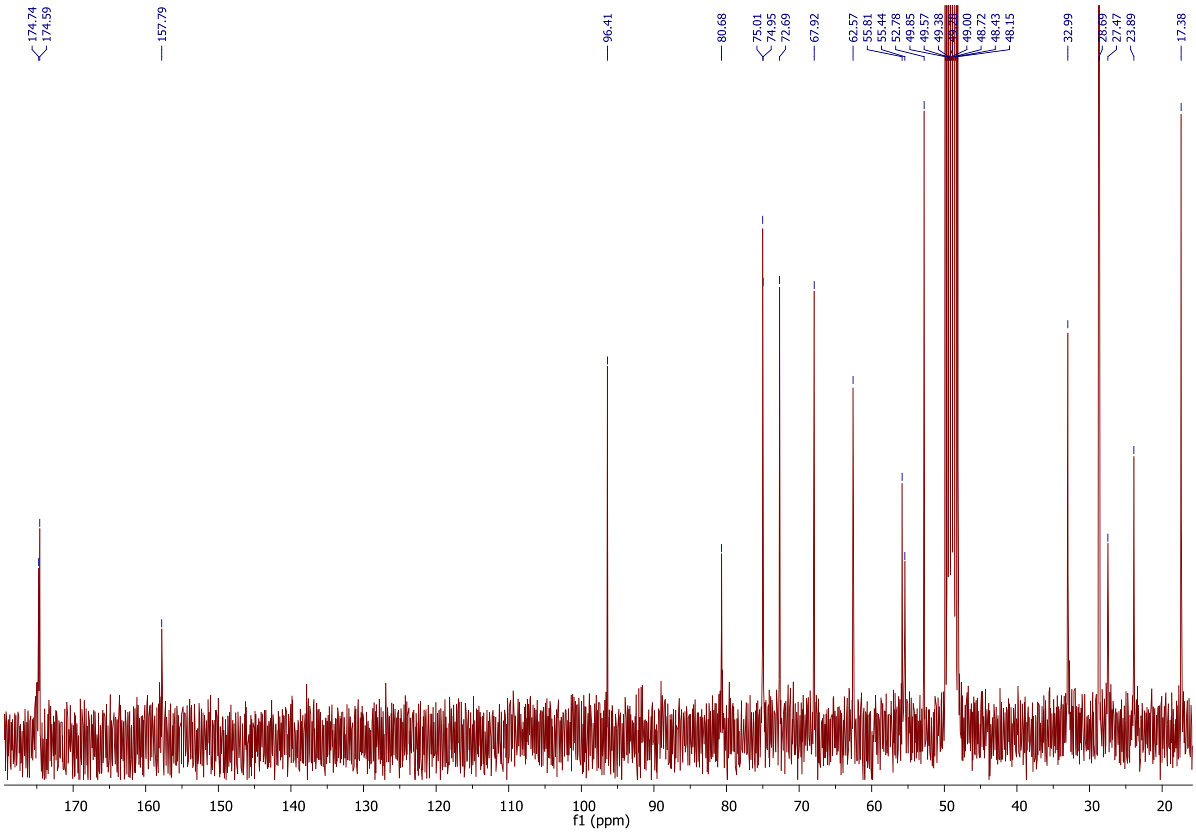

HRMS (MALDI): **37**

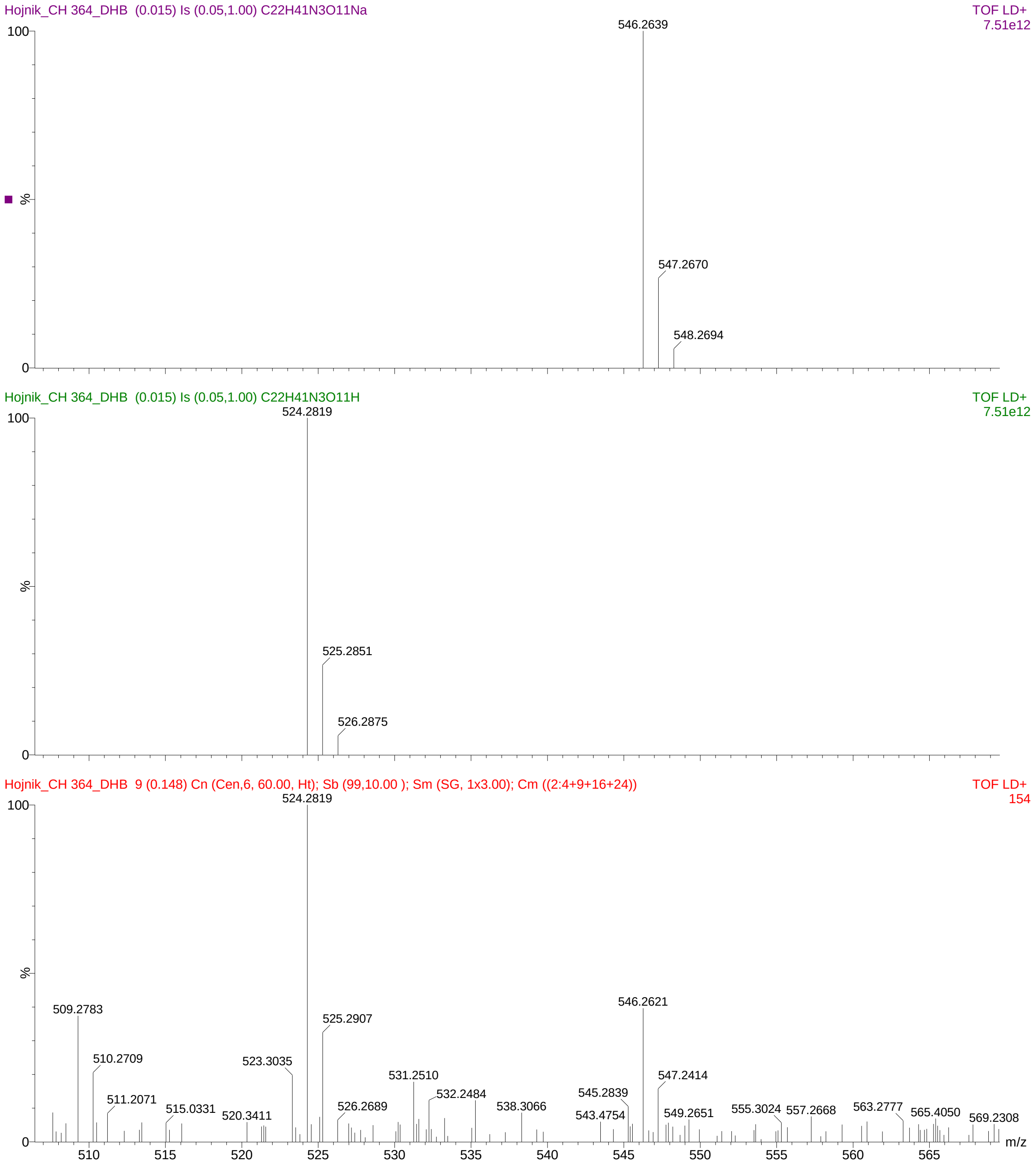

^{1}H and ^{13}C NMR spectra (**39**)

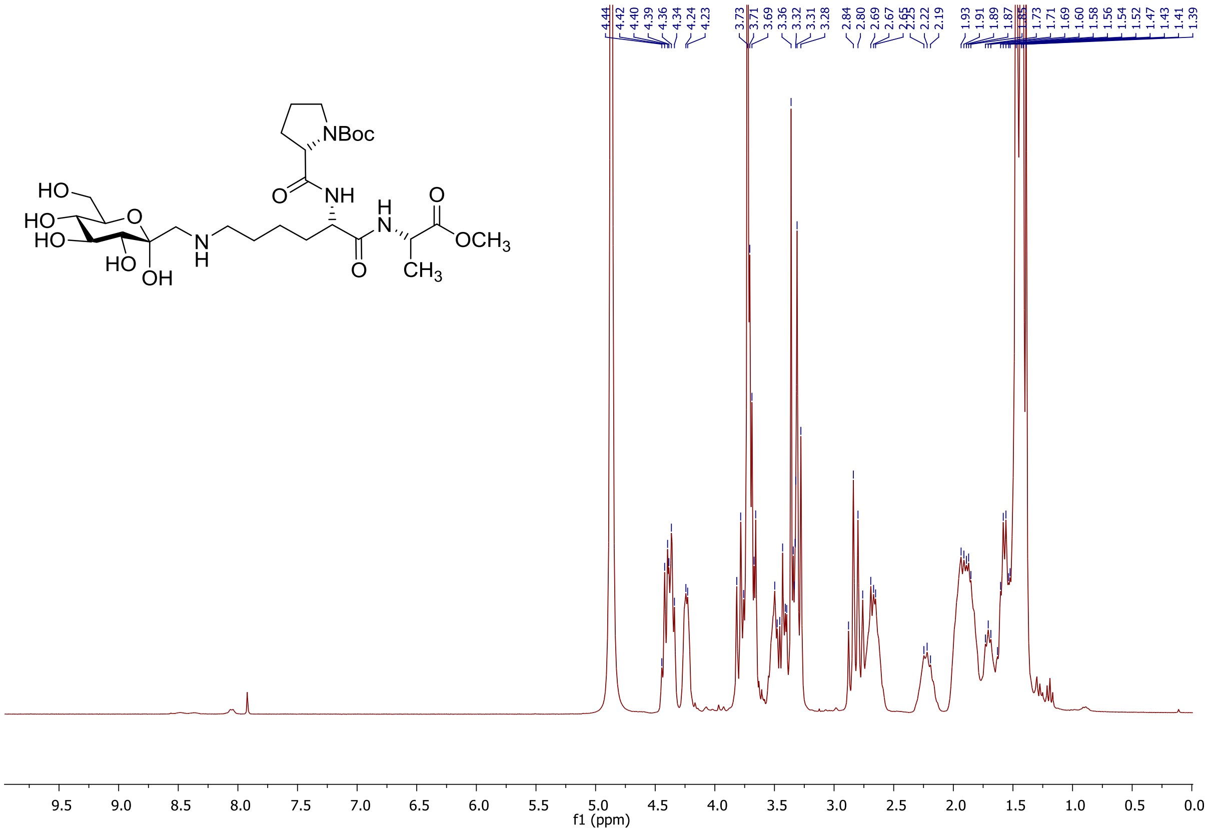

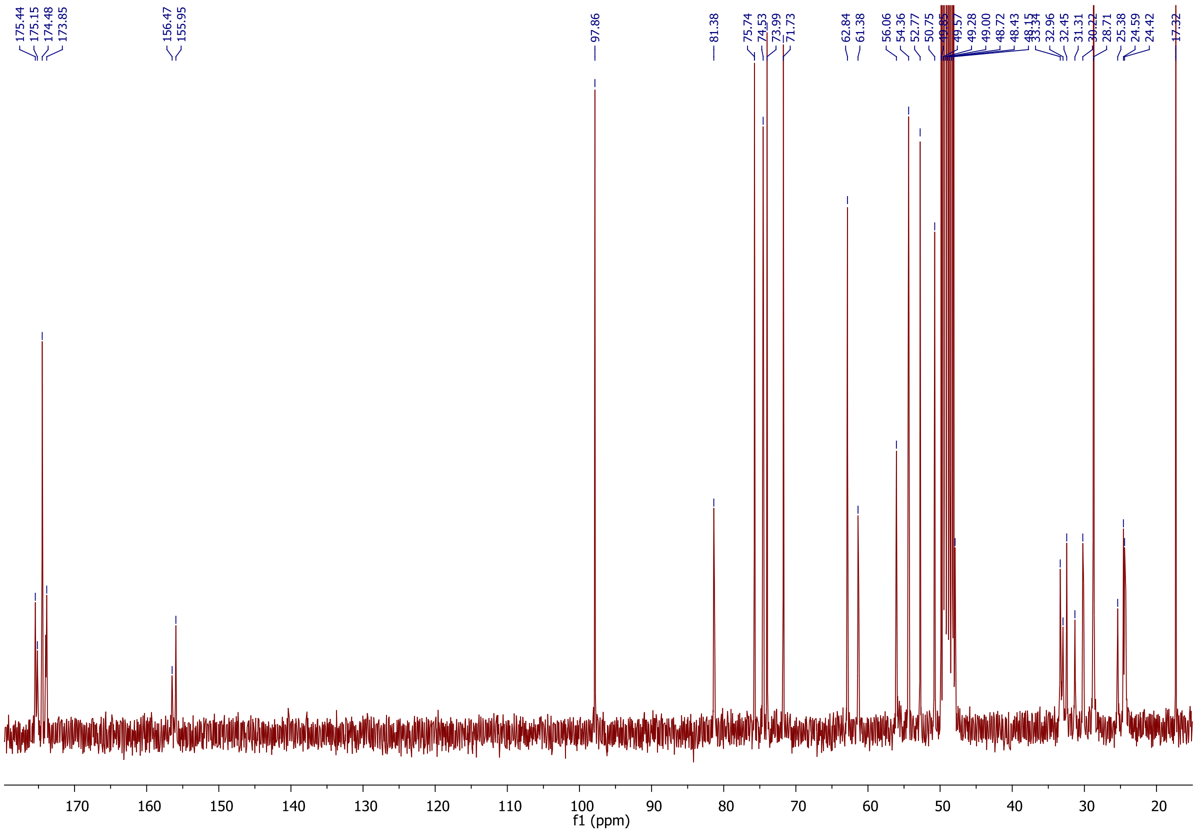

HRMS (MALDI): **39**

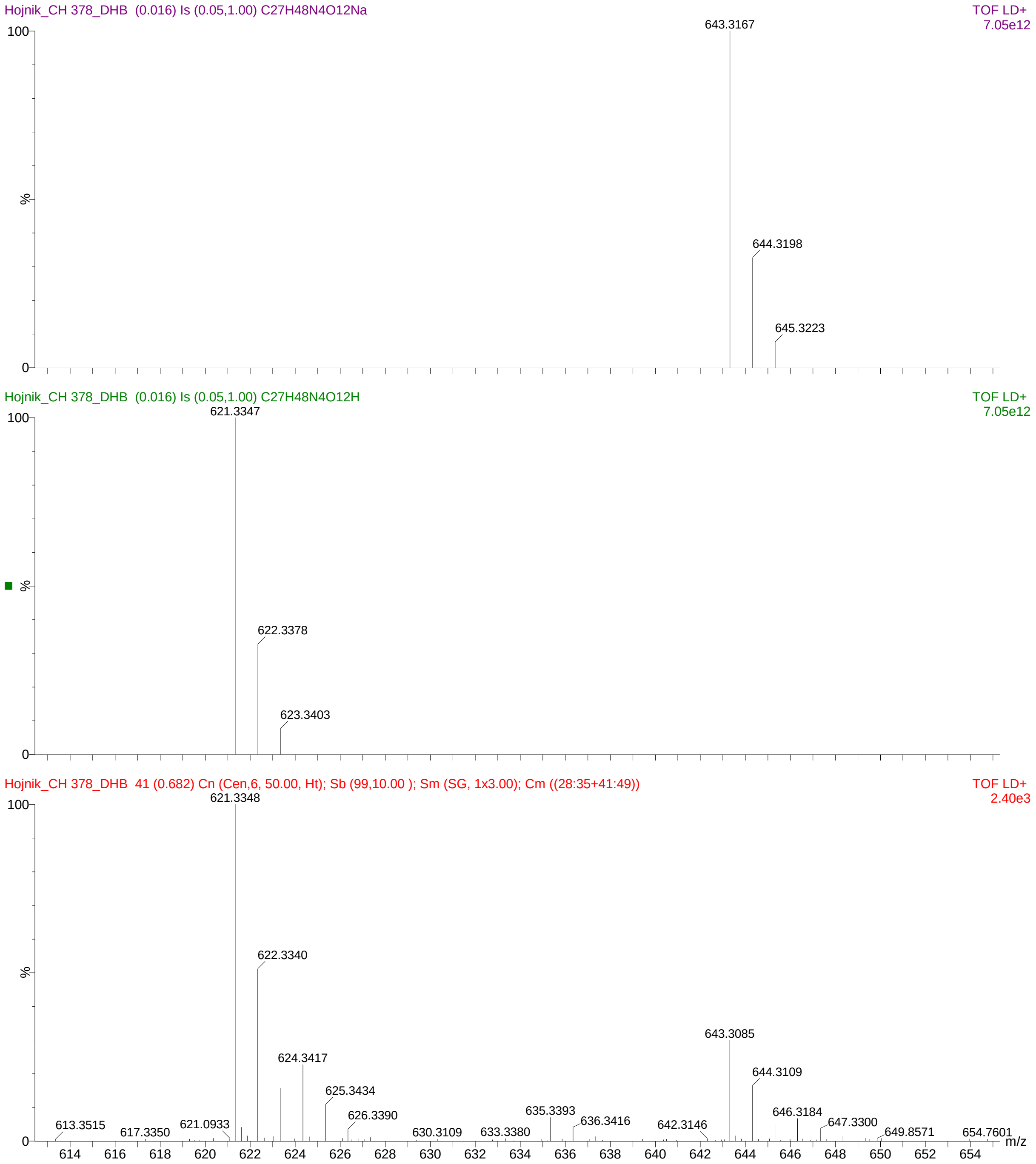

^{1}H and ^{13}C NMR spectra (**40**)

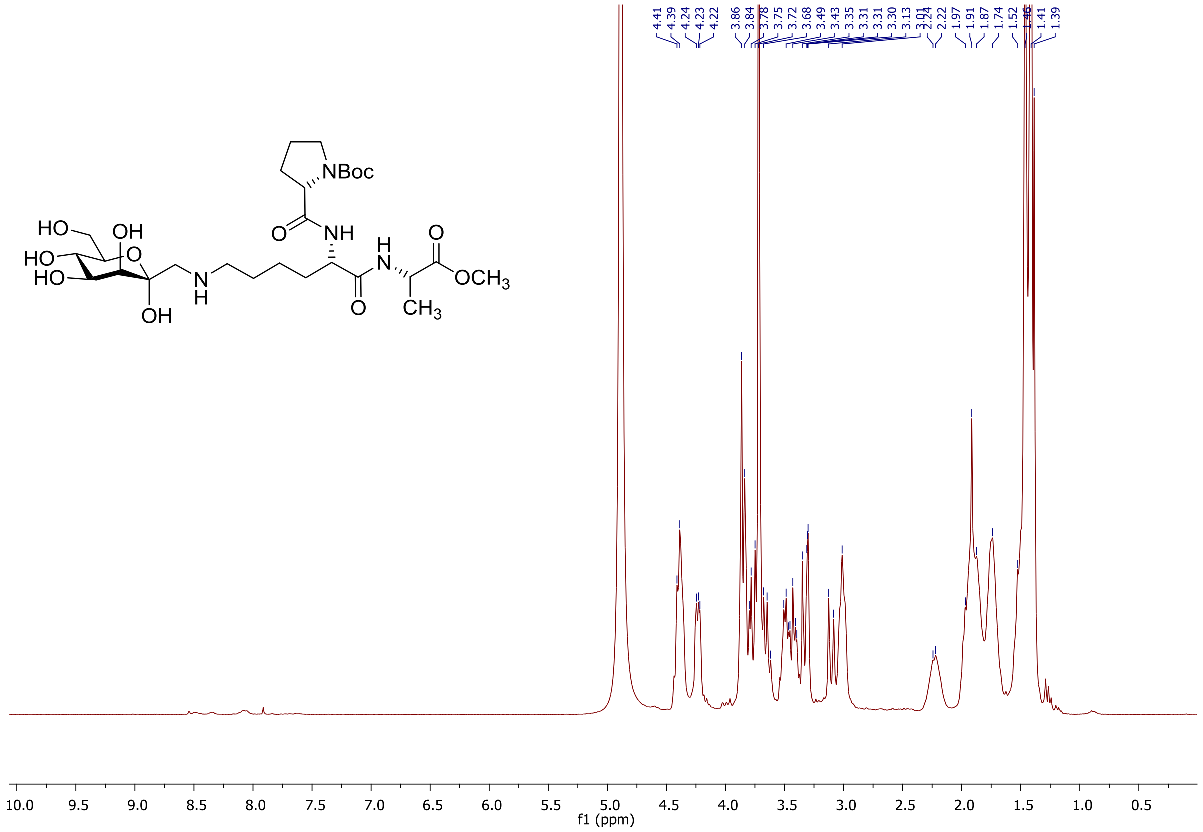

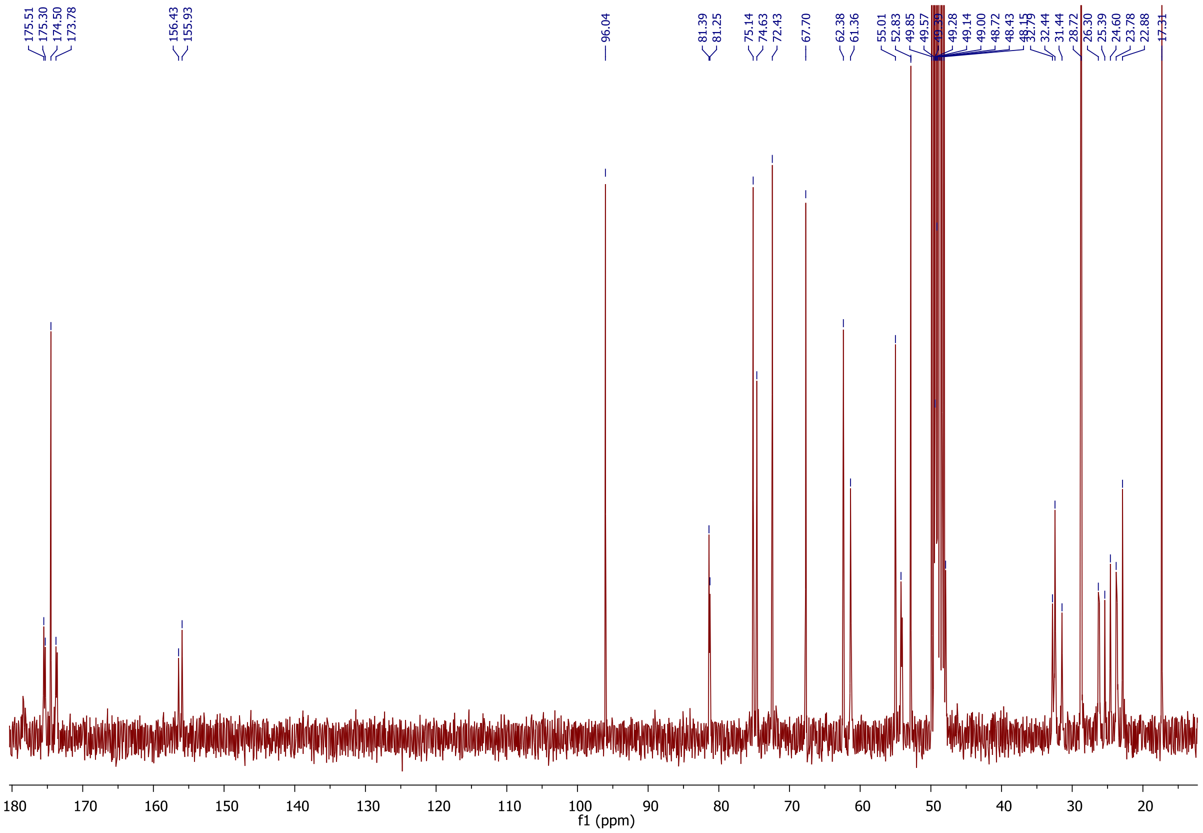

HRMS (MALDI): **40**

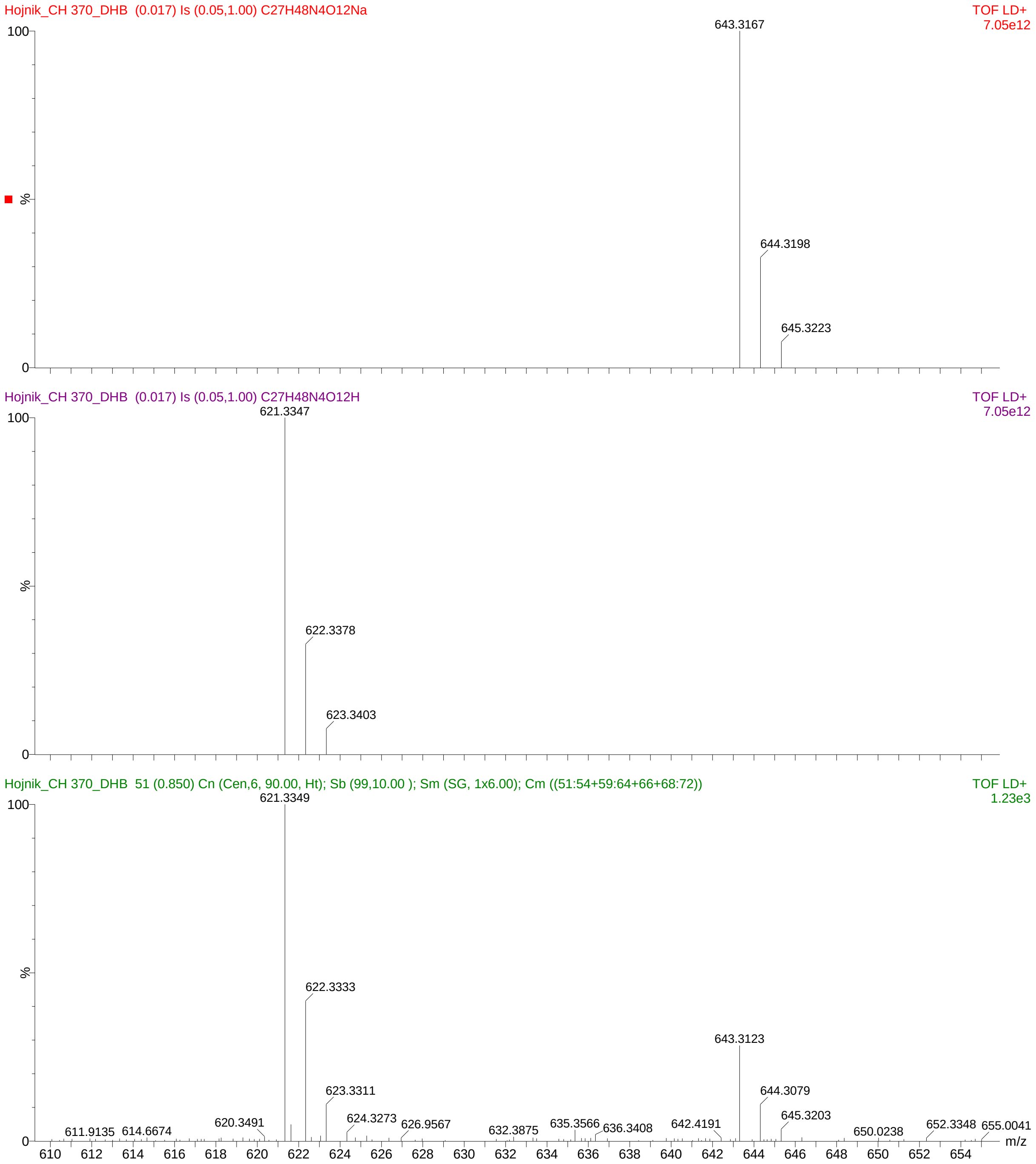

S3. H/D exchange of Amadori products

During our investigation applying various amines in the Amadori rearrangement we observed an H/D exchange at position C-1. This isotopic exchange was previously detected via NMR measurements by Heyns and coworkers[1] who noticed that signals of protons at the position C-1 in amino acid fructose of Amadori rearrangement compounds decreased on prolonged storage of solution in D_2O because of H/D exchange (Scheme 1). Furthermore, this exchange accelerates significantly with increasing basic pH values.[2]

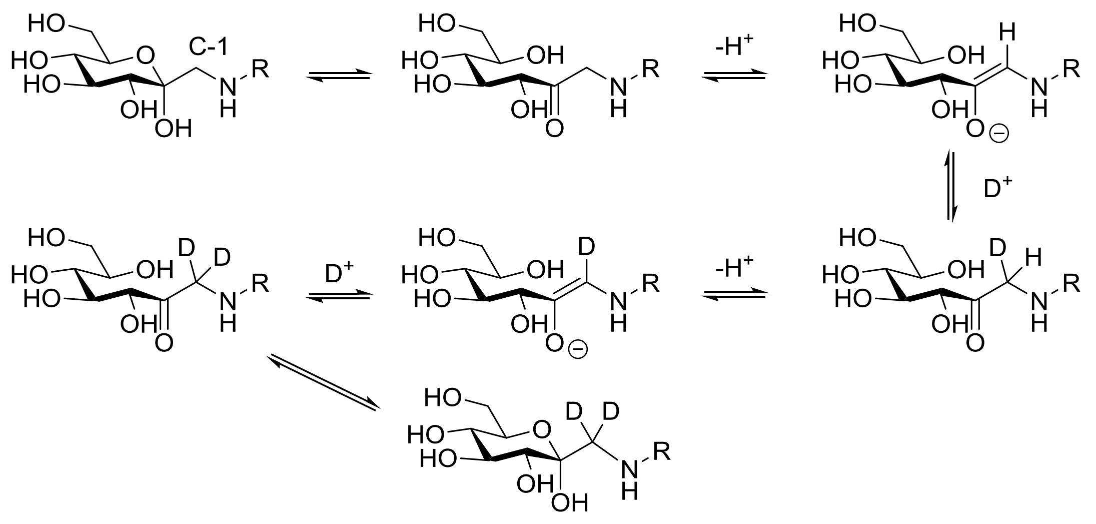

Scheme 1: Proposed mechanism of H/D exchange of Amadori products.[2]

Accordingly, we detected the H/D exchange at position C-1 of the synthesised Amadori rearrangement products in D_2O. In particular, due to the purification method described for compounds (**4-8** and **10-14**) employing ion exchange CG-120-II (Na^+) Amberlite resin column a pH value of about 9 was obtained in the product fraction. This basic pH value increased the rate of the isotopic exchange in D_2O solution significantly, after a few minutes the signals of both protons at position C-1 were not detectable in the proton NMR spectra and likewise the signal according to C-1 in the carbon NMR spectra splits into a pentett signal (Figure 1-5).

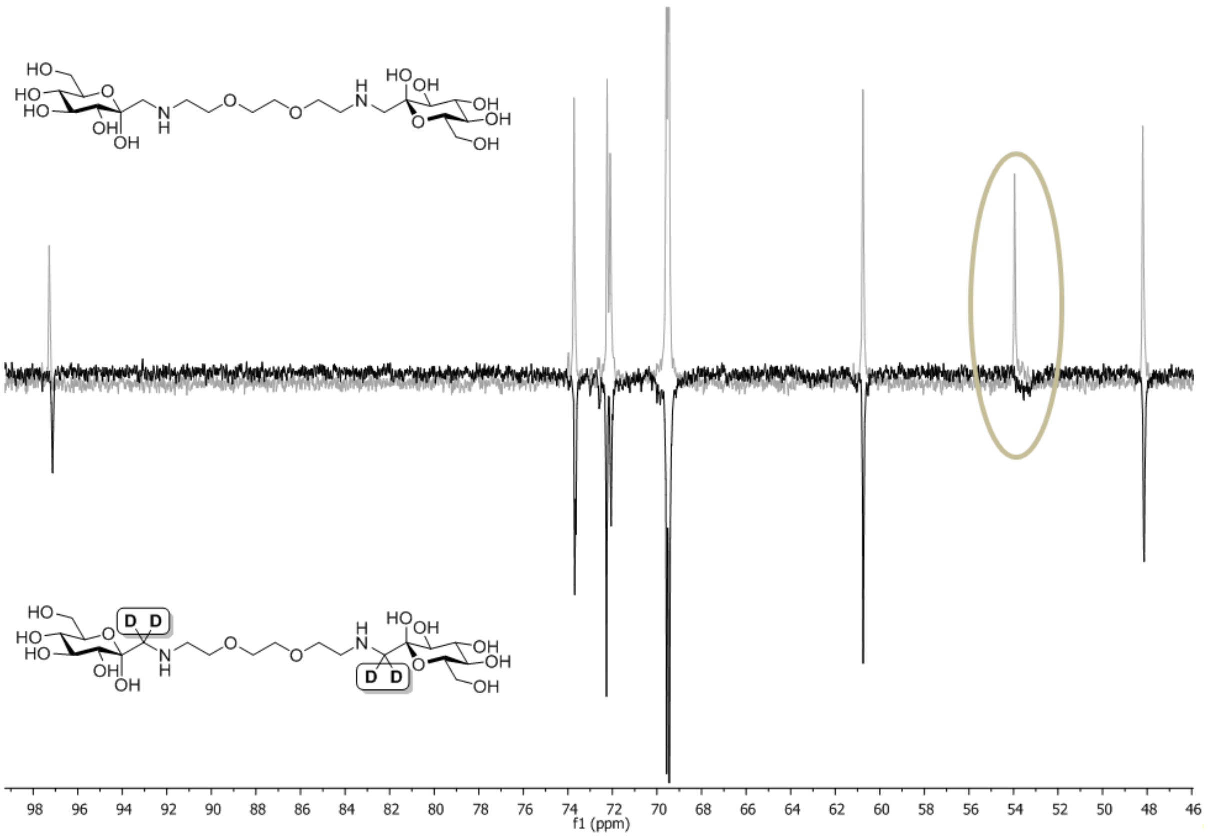

Figure 1: H/D exchange of Amadori product **7**.

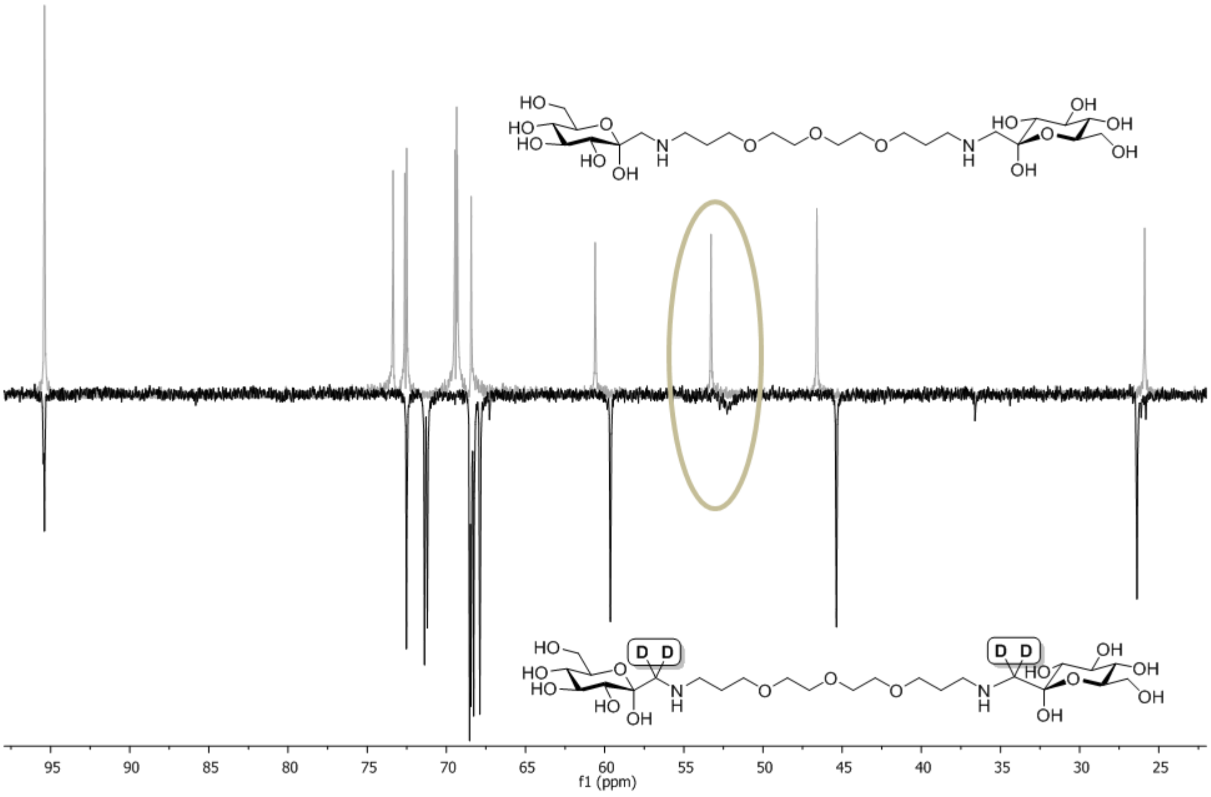

Figure 2: H/D exchange of Amadori product **8**.

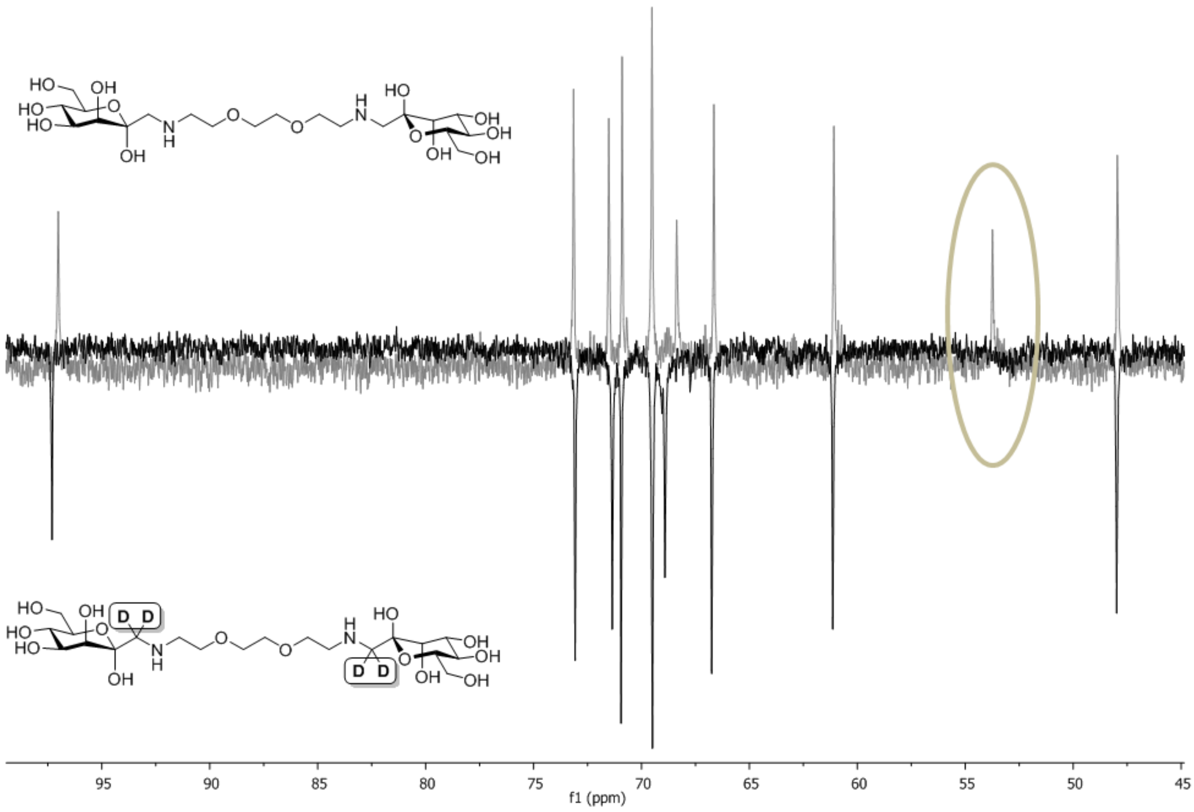

Figure 3: H/D exchange of Amadori product **13**.

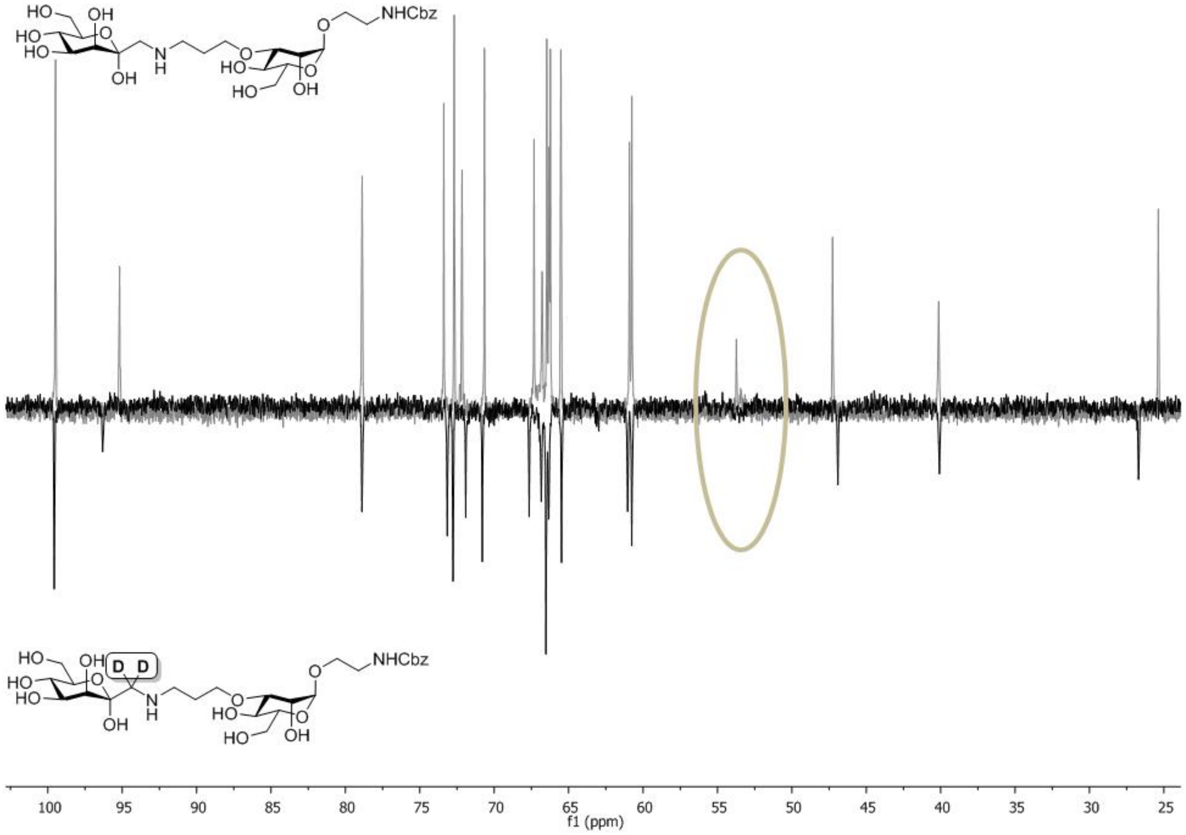

Figure 4: H/D exchange of Amadori product **27**.

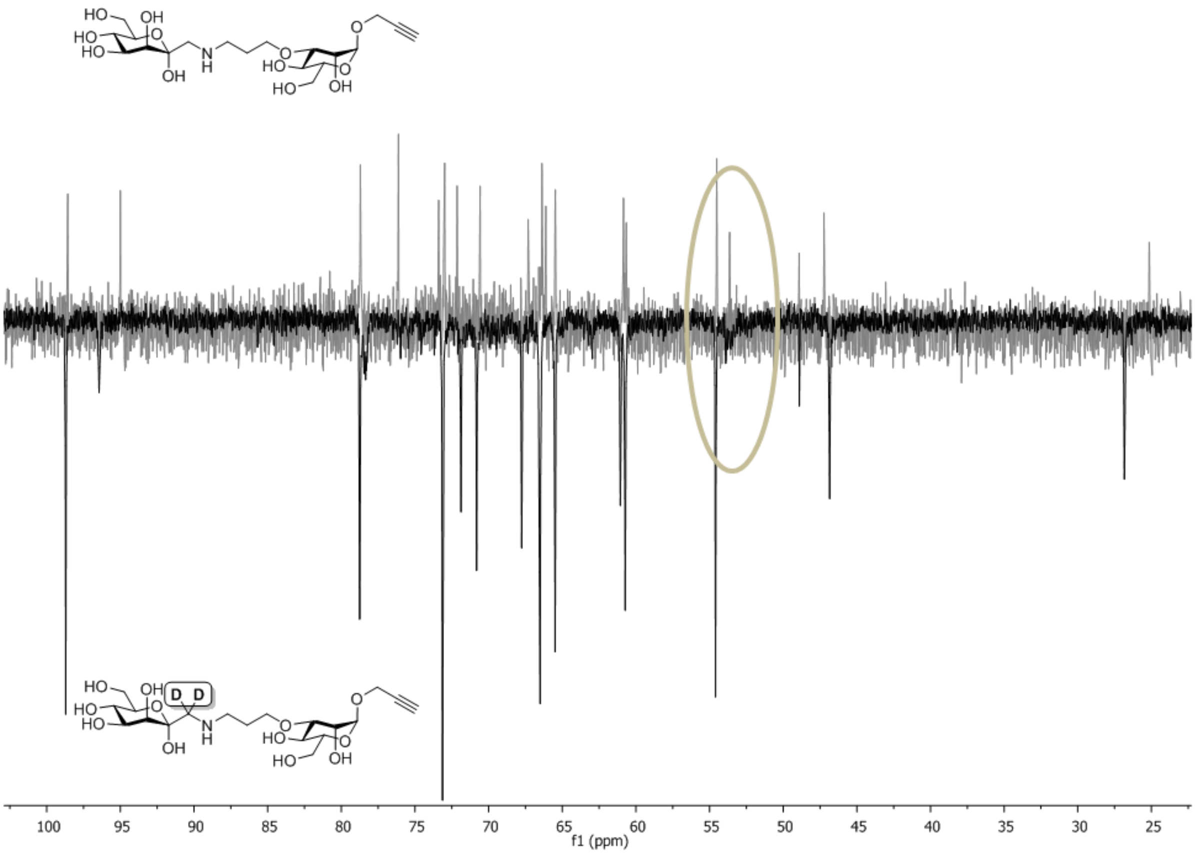

Figure 5: H/D exchange of Amadori product **28**.

We observed this phenomenon at the bivalent rearrangement products (**7**, **8** and **13**) as well as at the rearrangement products (**27** and **28**) using the amino-functionalized carbohydrate moieties when applying D_2O in the NMR measurements. In contrast, when DMSO-d_6 was employed as the solvent system for NMR measurements, as expected, no H/D exchange was observed.

S4. References

[1] H. Röper, S. Röper, K. Heyns, B. Meyer, *Carbohydr. Res.* **1983**, *116*, 183-195.

[2] K. Kapczyńska, P. Stefanowicz, Ł. Jaremko, M. Jaremko, A. Kluczyk, Z. Szewczuk, *Amino Acids* **2011**, *40*, 923-932.
